# ROS and cGMP signaling modulate persistent escape from hypoxia in *Caenorhabditis elegans*

**DOI:** 10.1371/journal.pbio.3001684

**Published:** 2022-06-21

**Authors:** Lina Zhao, Lorenz A. Fenk, Lars Nilsson, Niko Paresh Amin-Wetzel, Nelson Javier Ramirez-Suarez, Mario de Bono, Changchun Chen

**Affiliations:** 1 Umeå Centre for Molecular Medicine, Umeå University, Umeå, Sweden; 2 Wallenberg Centre for Molecular Medicine, Umeå University, Umeå, Sweden; 3 Umeå Centre for Microbial Research, Umeå University, Umeå, Sweden; 4 Max Planck Institute for Brain Research, Frankfurt am Main, Germany; 5 Institute of Science and Technology Austria (IST Austria), Klosterneuburg, Austria; UMass Chan Medical School, UNITED STATES

## Abstract

The ability to detect and respond to acute oxygen (O_2_) shortages is indispensable to aerobic life. The molecular mechanisms and circuits underlying this capacity are poorly understood. Here, we characterize the behavioral responses of feeding *Caenorhabditis elegans* to approximately 1% O_2_. Acute hypoxia triggers a bout of turning maneuvers followed by a persistent switch to rapid forward movement as animals seek to avoid and escape hypoxia. While the behavioral responses to 1% O_2_ closely resemble those evoked by 21% O_2_, they have distinct molecular and circuit underpinnings. Disrupting phosphodiesterases (PDEs), specific G proteins, or BBSome function inhibits escape from 1% O_2_ due to increased cGMP signaling. A primary source of cGMP is GCY-28, the ortholog of the atrial natriuretic peptide (ANP) receptor. cGMP activates the protein kinase G EGL-4 and enhances neuroendocrine secretion to inhibit acute responses to 1% O_2_. Triggering a rise in cGMP optogenetically in multiple neurons, including AIA interneurons, rapidly and reversibly inhibits escape from 1% O_2_. Ca^2+^ imaging reveals that a 7% to 1% O_2_ stimulus evokes a Ca^2+^ decrease in several neurons. Defects in mitochondrial complex I (MCI) and mitochondrial complex I (MCIII), which lead to persistently high reactive oxygen species (ROS), abrogate acute hypoxia responses. In particular, repressing the expression of *isp-1*, which encodes the iron sulfur protein of MCIII, inhibits escape from 1% O_2_ without affecting responses to 21% O_2_. Both genetic and pharmacological up-regulation of mitochondrial ROS increase cGMP levels, which contribute to the reduced hypoxia responses. Our results implicate ROS and precise regulation of intracellular cGMP in the modulation of acute responses to hypoxia by *C*. *elegans*.

## Abstract 2

The ability to detect and respond to acute oxygen shortages is indispensable to aerobic life, but the molecular mechanisms underlying this capacity are poorly understood. This study reveals that high levels of cGMP and reactive oxygen species (ROS) prevent the nematode Caenorhabditis elegans from escaping hypoxia.

## Introduction

Animals have evolved sophisticated mechanisms to maintain cellular homeostasis when encountering changes in oxygen (O_2_) availability. O_2_-sensing mechanisms, including in the carotid body, vascular smooth muscle cells, and chromaffin cells of fetal adrenal medulla, react acutely to hypoxia to ensure sufficient oxygenation of critical organs [1–5]. O_2_-sensitive cells respond to hypoxia by closure of K^+^ channels and subsequent opening of voltage-gated Ca^2+^ channels. A variety of models have been proposed for how a drop in O_2_ levels is detected and transmitted to the downstream K^+^ channels. Accumulating evidence suggests that the rapid decrease of O_2_ is detected by the mitochondria, which rapidly generate reactive oxygen species (ROS) to inhibit K^+^ channels on the plasma membrane [1,6–8]. Perturbation of mitochondrial complex I (MCI) or mitochondrial complex III (MCIII) leads to continuous ROS production, which subsequently prevents acute response to hypoxia [1,6–8]. However, it remains unclear whether mitochondrial ROS directly modifies K^+^ channels on the plasma membrane.

Several studies implicate cGMP signaling in acute hypoxia sensing, with cGMP inhibiting the O_2_ sensitivity of chemosensory organs [9–12]. For example, atrial natriuretic peptide (ANP), which enhances cGMP production, dampens acute vasoconstriction of the pulmonary artery in response to hypoxia [9,12]. Similarly, nitric oxide (NO) stimulated cGMP production represses the sensory activity of the carotid body [11]. How a rise in cGMP blocks the sensitivity of O_2_ sensors is unclear. By contrast, it is well established in the nematode *Caenorhabditis elegans* that cGMP is the primary second messenger mediating responses to acute hyperoxia (21% O_2_). *C*. *elegans* avoids both atmospheric (21%) and hypoxic (1%) O_2_ concentrations [13]. When O_2_ levels rise from 7% to 21%, atypical soluble guanylate cyclases in the O_2_ sensing neurons URX, AQR, and PQR that directly bind O_2_ become progressively activated, elevating cGMP levels, which opens cyclic nucleotide gated (CNG) channels and causes neuron depolarization [13–19].

The remarkable ability of *C*. *elegans* to tolerate extreme O_2_ concentrations, from near anoxia to 100% O_2_ [20], makes it a good model to study O_2_ responses in an intact, behaving, animal. By contrast to our detailed understanding of avoidance and escape from hyperoxia, little is known about how *C*. *elegans* responds to acute hypoxia [20,21]. Here, we characterize changes in *C*. *elegans’* locomotory behavior when O_2_ levels drop abruptly to hypoxic levels and delineate the importance of intracellular cGMP and mitochondrial ROS in the regulation of these responses.

## Results

### Hypoxia evokes immediate and sustained changes in *C*. *elegans* behavior

We set out to examine the effect of acute hypoxia on *C*. *elegans* locomotory behavior. A sudden decrease from 7% to 1% O_2_ elicited a bout of turning maneuvers (omega turns and reversals, S1A and S1B Fig) followed by a switch to rapid forward movement (S1C Fig). Strikingly, while the increased turning behavior was transient, the locomotory arousal elicited by hypoxia was sustained for as long as O_2_ levels remained low and showed no sign of adapting to repeated stimulation (S1D and S1E Fig). Acute hypoxia thus evokes both transient and sustained changes in *C*. *elegans* locomotory behavior, implying the existence of fast and slowly adapting neuronal mechanisms.

To explore behavioral responses to more severe hypoxia, we shifted animals from 7% O_2_ to pure nitrogen. The microfluidic chamber arena we used for our behavioral assays was not completely gastight, so pumping pure nitrogen into the chamber resulted in measured O_2_ levels of approximately 0.2% (henceforth “near-anoxia”), whereas we measured approximately 1.2% O_2_ (hypoxia) when we pumped 1% O_2_ into the chamber. A shift to near-anoxia elicited a sharp increase in speed of locomotion that peaked soon after stimulation and then decreased slowly (S1F Fig). The speeds reached by animals in near-anoxia were lower than those of animals in hypoxia (S1F Fig). When switched back from near-anoxia to 7% O_2_, animals did not reduce speed immediately and often transiently increased their locomotory activity before slowing down (S1F Fig). These observations, together with earlier work [22,23], suggest acute behavioral responses to hypoxia and anoxia differ both quantitatively and qualitatively [20].

**Fig 1:**
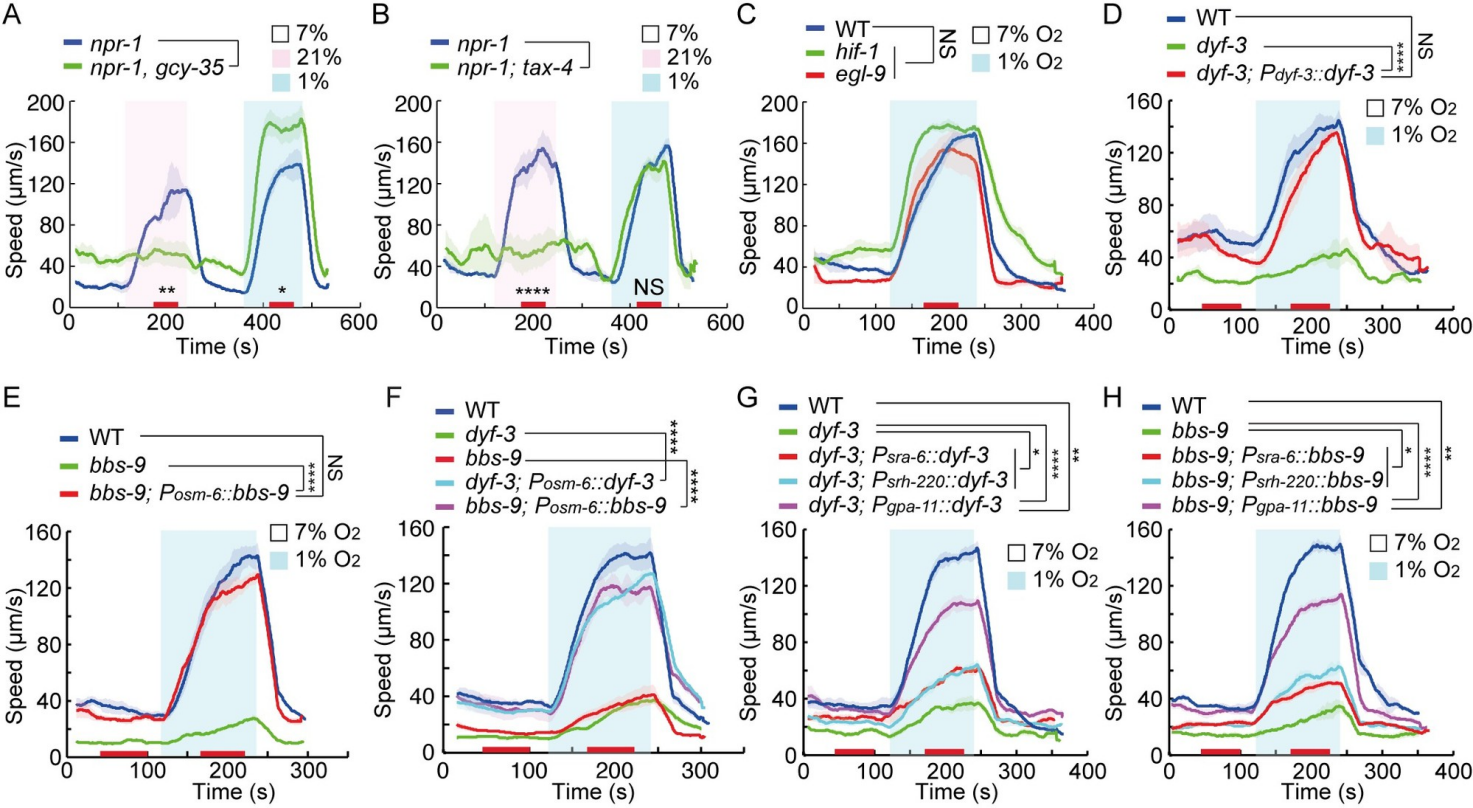
Defects in IFT disrupt escape from hypoxia. **(A and B)** Locomotory responses to switches from 7% to 21% O_2_ and 7% to 1% O_2_ of animals of indicated genotype: *npr-1(ad609)* and *npr-1(ad609); gcy-35(ok769)* in (A) and *npr-1(ad609)* and *npr-1(ad609); tax-4(p678)* in (B). In this and subsequent figures, speed was recorded for 2 minutes at each O_2_ interval. Red bars on the x-axis indicate time intervals used for statistical analysis. Data for each genotype are from 3 to 4 assays, >80 animals. **** = *p* < 0.0001, ** = *p* < 0.01, * = *p* < 0.05, NS = not significant, Mann–Whitney U test. **(C)** Locomotory responses to 7% to 1% O_2_ stimuli for animals of the indicated genotype: WT (N2), *hif-1(ia4)*, and *egl-9(sa307)* animals. NS = not significant. ANOVA, Tukey multiple comparison. **(D)** Locomotory responses to 7% to 1% O_2_ stimuli for animals of the indicated genotype: WT, *dyf-3(m185)*, and *dyf-3(m185)* expressing *dyf-3* cDNA from its endogenous promoter. Statistical comparison of the locomotory speed at 7% and 1% O_2_ of each genotype: WT (****), *dyf-3(m185)* (NS), and *dyf-3(m185)* expressing *dyf-3* cDNA from its endogenous promoter (****). **** = *p* < 0.0001, NS = not significant. ANOVA, Tukey multiple comparison. Statistical analysis of the locomotory speed at 1% O_2_ of different genotypes were displayed in the figure. **** = *p* < 0.0001, NS = not significant. ANOVA, Tukey multiple comparison. **(E)** Locomotory responses to 7% to 1% O_2_ stimuli for animals of the indicated genotype: WT, *bbs-9(gk471)*, and *bbs-9(gk471)* expressing *bbs-9* cDNA in ciliated neurons from the *osm-6* promoter. Statistical comparison of the locomotory speed at 7% and 1% O_2_ of each genotype: WT (****), *bbs-9(gk471)* (NS), and *bbs-9(gk471)* expressing *bbs-9* cDNA in ciliated neurons from the *osm-6* promoter (****). **** = *p* < 0.0001, NS = not significant. ANOVA, Tukey multiple comparison. Statistical analysis of the locomotory speed at 1% O_2_ of different genotypes were displayed in the figure. **** = *p* < 0.0001, NS = not significant. ANOVA, Tukey multiple comparison. **(F)** Locomotory responses to 7% to 1% O_2_ stimuli for animals of the indicated genotype: WT, *dyf-3(m185)*, and *dyf-3(m185)* expressing *dyf-3* cDNA in ciliated neurons from the *osm-6* promoter, *bbs-9(gk471)*, and *bbs-9(gk471)* expressing *bbs-9* cDNA in ciliated neurons from the *osm-6* promoter. Statistical comparison of the locomotory speed at 7% and 1% O_2_ of each genotype: WT (****), *dyf-3(m185)* (NS), and *dyf-3(m185)* expressing *dyf-3* cDNA in ciliated neurons from the *osm-6* promoter (****), *bbs-9(gk471)* (NS), and *bbs-9(gk471)* expressing *bbs-9* cDNA in ciliated neurons from the *osm-6* promoter (****). **** = *p* < 0.0001, NS = not significant. ANOVA, Tukey multiple comparison. Statistical analysis of the locomotory speed at 1% O_2_ of different genotypes were displayed in the figure. **** = *p* < 0.0001. ANOVA, Tukey multiple comparison. **(G)** Locomotory responses to 7% to 1% O_2_ stimuli for animals of the indicated genotype: WT, *dyf-3(m185)*, and *dyf-3(m185)* mutants expressing *dyf-3* cDNA from the *sra-6* (ASH), *srh-220* (ADL), and *gpa-11* (ASH and ADL) promoters. Statistical comparison of the locomotory speed at 7% and 1% O_2_ of each genotype: WT (****), *dyf-3(m185)* (*), and *dyf-3(m185)* mutants expressing *dyf-3* cDNA from the *sra-6* promoter (***), *srh-220* promoter (****), and *gpa-11* promoter (****). **** = *p* < 0.0001, *** = *p* < 0.001, * = *p* < 0.05. ANOVA, Tukey multiple comparison. Statistical analysis of the locomotory speed at 1% O_2_ of different genotypes were displayed in the figure. **** = *p* < 0.0001, ** = *p* < 0.01, * = *p* < 0.05. ANOVA, Tukey multiple comparison. **(H)** Locomotory responses to 7% to 1% O_2_ stimuli for animals of the indicated genotype: WT, *bbs-9(gk471)*, and *bbs-9(gk471)* mutants expressing *bbs-9* cDNA from the *sra-6* (ASH), *srh-220* (ADL), and *gpa-11* (ASH and ADL) promoters. Statistical comparison of the locomotory speed at 7% and 1% O_2_ of each genotype: WT (****), *bbs-9(gk471)* (NS), and *bbs-9(gk471)* mutants expressing *bbs-9* cDNA from the *sra-6* promoter (***), *srh-220* promoter (****), and *gpa-11* promoter (****). **** = *p* < 0.0001, *** = *p* < 0.001, NS = not significant. ANOVA, Tukey multiple comparison. Statistical analysis of the locomotory speed at 1% O_2_ of different genotypes were displayed in the figure. **** = *p* < 0.0001, ** = *p* < 0.01, * = *p* < 0.05. ANOVA, Tukey multiple comparison. The source code underlying behavioral data can be found at https://github.com/wormtracker/zentracker. IFT, intraflagellar transport; O_2_, oxygen; WT, wild-type.

The behavioral responses to rapid up- and downshifts in O_2_, from 7% to 21% and from 7% to 1% O_2_, were remarkably similar [14,16], prompting us to ask if they share sensory pathways. Avoidance and escape from 21% O_2_ are driven principally by AQR, PQR, and URX neurons and requires the heteromeric O_2_-binding soluble guanylate cyclase GCY-35/GCY-36 that acts upstream of the TAX-4/TAX-2 cGMP-gated ion channels [13–18]. Since the standard *C*. *elegans* lab strain, N2, responds weakly to 21% O_2_, due to a gain-of-function mutation in the *npr-1* neuropeptide gene [13,24], we used an *npr-1* null background to examine acute responses to 21% and 1% O_2_ in the same animals. *npr-1* mutants responded normally to 1% O_2_ (S1G Fig). While *gcy-35; npr-1* and *tax-4; npr-1* null mutants showed defective responses to 21% O_2_, they reacted robustly to 1% O_2_ (Fig 1A and 1B). Thus, distinct mechanisms underlie acute detection of hyperoxia and hypoxia in *C*. *elegans*. Responses to chronic hypoxia require the transcription factor HIF-1 and its negative regulator EGL-9 [25]. Both *hif-1* and *egl-9* mutants dramatically increased their locomotory speed in response to 1% O_2_, suggesting that responses to acute hypoxia do not require the core machinery mediating chronic hypoxia responses (Fig 1C).

### Mutants lacking the BBSome show disrupted responses to acute hypoxia

To identify molecules that mediate or regulate acute responses to hypoxia, we performed a candidate screen for mutants that failed to increase their locomotory speed when O_2_ levels acutely dropped from 7% to 1%. Our screen included mutants defective in guanylate cyclases, globins, neuropeptides, potassium channels, G protein signaling, cilia intraflagellar transport (IFT) and mitochondrial electron transport (S1 Table). This screen yielded multiple strains with strong defects in their responses to acute hypoxia. A subset of these strains contain loss-of-function mutations in *bbs* genes, encoding the subunits of Bardet–Biedel syndrome (BBS) protein complex BBSome [26], and in the *dyf-3* gene, whose product plays a role in IFT and is the ortholog of vertebrate qilin/cluap1 [27–30] (S1 Table). CLUAP1 is the only member of the IFT-B complex that reliably co-immunoprecipitates with BBSome [26], which traffics structural and signaling components into and out of cilia [31–34]. *dyf-3* animals moved more slowly at 7% O_2_ and showed a greatly reduced behavioral response to hypoxia (Fig 1D). This phenotype could be rescued by a wild-type copy of *dyf-3* (Fig 1D). *bbs* mutants, including *bbs-1*, *bbs-2*, *bbs-4*, *bbs-7*, *bbs-8*, and *bbs-9*, each displayed low basal movement at 7% O_2_ and strong suppression of hypoxia-evoked behavioral responses (Figs 1E, S2A and S2B and S1 Table). Expressing *bbs-9* cDNA in ciliated neurons fully restored the hypoxia response to *bbs-9* mutants (Fig 1E). Mutants defective in other components of the IFT machinery, or in ciliogenesis, also showed defects in response to acute hypoxia, to varying degrees (S1 Table). These data implicate cilia in the hypoxia response.

Attenuated behavioral responses to 1% O_2_ in cilia-defective mutants could reflect more general deficiencies and reduced locomotion in mutant animals, rather than a specific defect in the ability to sense hypoxia. To probe this, we examined whether mutations in *bbs-9* and *dyf-3* impaired the very similar behavioral responses to 21% O_2_, using an *npr-1* null background. Both *dyf-3; npr-1* and *bbs-9; npr-1* mutants were strongly aroused by 21% O_2_ but not by 1% O_2_ (S2C and S2D Fig). Thus, the reduced behavioral response of *dyf-3* and *bbs-9* mutants to hypoxia cannot be explained by a general locomotory defect, but reflects an inability of *C*. *elegans* to respond appropriately to a drop in ambient O_2_.

We next sought to identify the neurons where DYF-3 and the BBSome act to promote acute hypoxia responses. Expressing *bbs-9* or *dyf-3* cDNAs in all ciliated neurons, using the *osm-6* promoter, fully restored acute hypoxia responses to the respective mutants (Fig 1F). Selective expression in the ADL and ASH neuron pairs using the *gpa-11* promoter gave partial albeit significant rescue (Fig 1G and 1H). Selective expression in either ADL (*sra-6p*) or ASH (*srh-220p*) neurons alone gave weak but still significant rescue (Fig 1G and 1H). Cell-specific inactivation of *bbs-9* by sense and antisense RNA interference (RNAi) constructs [35] in ADL and ASH neurons partially inhibited acute response to hypoxia (S2E Fig). These data suggest that DYF-3 and BBSome act in ADL and ASH, as well as other ciliated neurons, to promote acute responses to hypoxia.

### Increased cGMP signaling inhibits acute hypoxia responses

**Fig 2:**
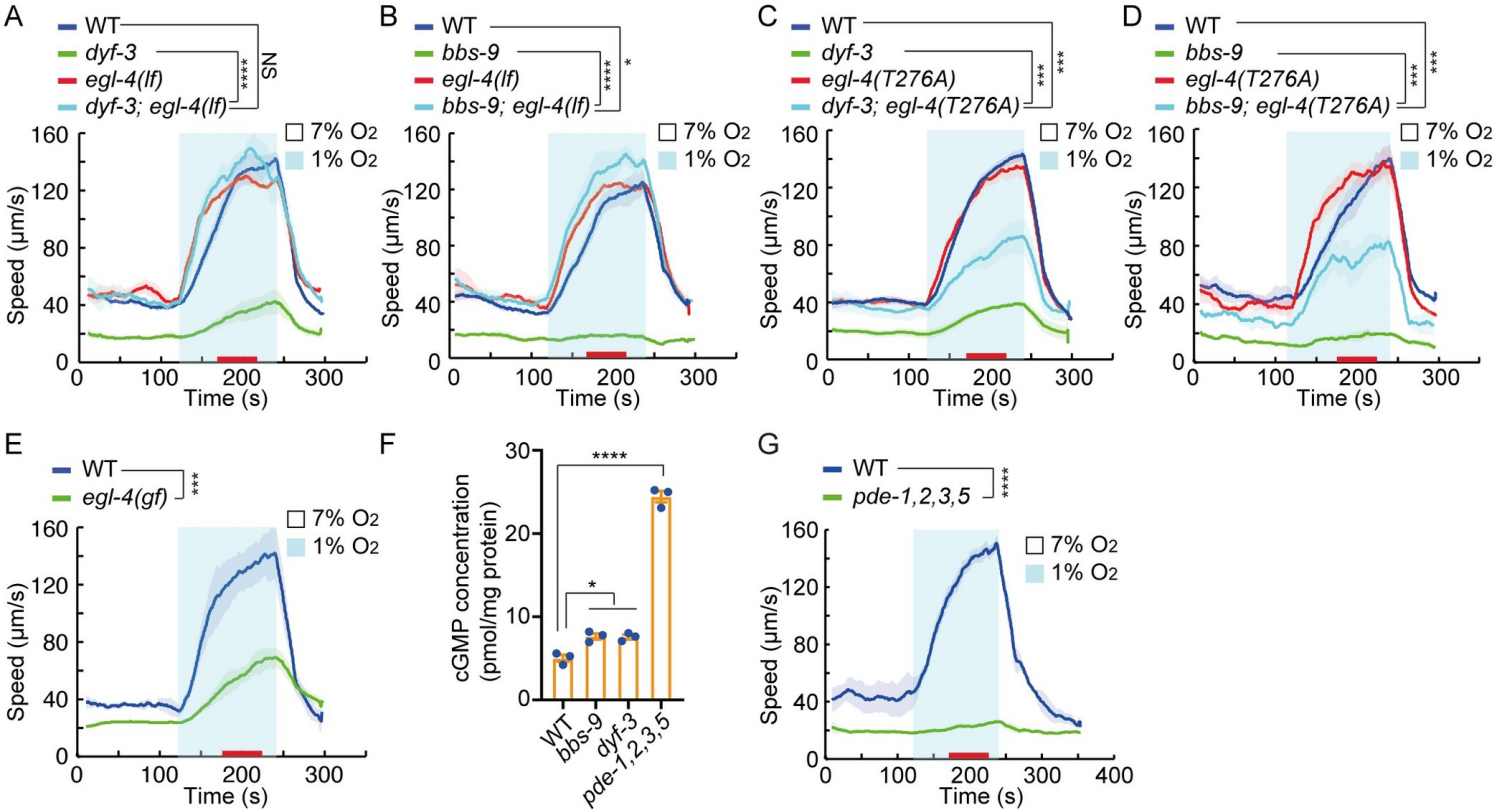
High EGL-4/PKG activity inhibits the acute hypoxia response. **(A–E)** Locomotory responses to 7% to 1% O_2_ stimuli for animals of the indicated genotypes: WT, *dyf-3(m185)*, *egl-4(n478)*, and *dyf-3(m185)*; *egl-4(n478)* double mutants (A); WT, *bbs-9(gk471)*, *egl-4(n478)*, and *bbs-9(gk471)*; *egl-4(n478)* double mutants (B); WT, *dyf-3(m185)*, *egl-4(T276A)*, and *dyf-3(m185)*; *egl-4(T276A)* double mutants (C); WT, *bbs-9(gk471)*, *egl-4(T276A)*, and *bbs-9(gk471)*; *egl-4(T276A)* double mutants (D). T276A in C and D stands for a threonine to alanine substitution at residue 276, which disrupts the low cGMP binding site of EGL-4. WT and *egl-4(ad450)* (E); *egl-4(ad450)* is a gain-of-function allele of *egl-4*. **** = *p* < 0.0001, *** = *p* < 0.001, * = *p* < 0.05, NS = not significant. ANOVA, Tukey multiple comparison (A–D), and Mann–Whitney U test (E). **(F)** Total cGMP levels in animal lysates of indicated genotype measured by a cGMP enzyme immunoassay (*n* = 3). Error bars = SEM. * = *p < 0*.*05*, **** = *p* < 0.0001. ANOVA, Tukey multiple comparison. **(G)** Locomotory responses to 7% to 1% O_2_ stimuli for animals of the indicated genotype: WT and *pde-1(nj57); pde-2(tm3098); pde-3(nj59); pde-5(nj49)* quadruple mutants (*pde-1*, *2*, *3*, *5*). **** = *p* < 0.0001, Mann–Whitney U test. The underlying data can be found in S1 Data, and the source code can be found at https://github.com/wormtracker/zentracker. O_2_, oxygen; WT, wild-type.

Earlier studies have shown that defects in IFT lead to increased cGMP/PKG signaling [36,37] and that loss-of-function mutations in genes encoding guanylate cyclases, or in the cGMP-dependent protein kinase (PKG) *egl-4*, can suppress a subset of phenotypes associated with IFT defects [36–39]. We therefore tested whether disrupting *egl-4* suppressed the hypoxia response defects of *dyf-3* and *bbs-9* mutants. A loss-of-function mutation in *egl-4* restored the hypoxia responses of both *dyf-3* and *bbs-9* mutants to wild-type levels (Fig 2A and 2B). One possible mechanism underlying suppression of the *bbs-9* hypoxia phenotype is restoration of cilium function to *egl-4; bbs-9* double mutants; however, the dye filling defects of *bbs-9* mutants were not suppressed by loss of *egl-4* (S3A and S3B Fig). EGL-4 contains a low and a high affinity cGMP binding site. Mutating either site abolishes EGL-4 nuclear localization [40,41]. The hypoxia response defect of *dyf-3* and *bbs-9* mutants was partially suppressed when the low affinity cGMP binding site of the endogenous *egl-4* locus was edited using CRISPR/Cas-9 (Fig 2C and 2D). In addition, an *egl-4* gain-of-function mutation, which enhances PKG signaling [42], potently inhibited acute responses to 1% O_2_ (Fig 2E). We conclude that constitutive high PKG activity inhibits acute hypoxia responses.

Our data are consistent with *dyf-3* and *bbs* mutants having elevated intracellular cGMP, leading to high EGL-4/PKG activity. To quantify total cGMP levels in these animals, we used ELISA of whole worm lysates [43]. As a positive control, we measured cGMP in mutants defective in the 4 *C*. *elegans* phosphodiesterases (PDEs) that hydrolyse cGMP, PDE-1, 2, 3, and 5. As expected, we observed a substantial increase in cGMP in *pde-1*,*2*,*3*,*5* quadruple mutants. We also observed a small but significant cGMP rise in *bbs-9* and *dyf-3* mutants (Fig 2F). *pde-1*,*2*,*3*,*5* quadruple mutants were completely defective in hypoxia-evoked locomotory responses, supporting the notion that high intracellular cGMP levels inhibit arousal in response to hypoxia (Fig 2G). To analyze this further, we assessed *pde* single and all possible double mutants and found that *pde-2*; *pde-3* animals were as defective as *pde* quadruple mutants (S4A–S4D Fig). By contrast, mutants lacking the 2 cAMP-specific PDEs, *pde-4* and *pde-6*, responded robustly to acute hypoxia (S4E Fig).

CNG channels are another major effector of cGMP besides PKG. However, mutations in the *C*. *elegans* CNG channel genes *tax-4*, *cng-1*, *cng-3*, and *cng-4* did not significantly alter acute hypoxia responses, and only *cng-1; cng-3* weakly suppressed the hypoxia response defects of *bbs-9* mutants (S4F–S4H Fig). Activation of CNG channels is therefore unlikely to be required either for acute hypoxia sensing or for suppressing acute hypoxia responses in *bbs-9* mutants.

### Disrupting GCY-28 suppresses the hypoxia response defects of *bbs* mutants

**Fig 3:**
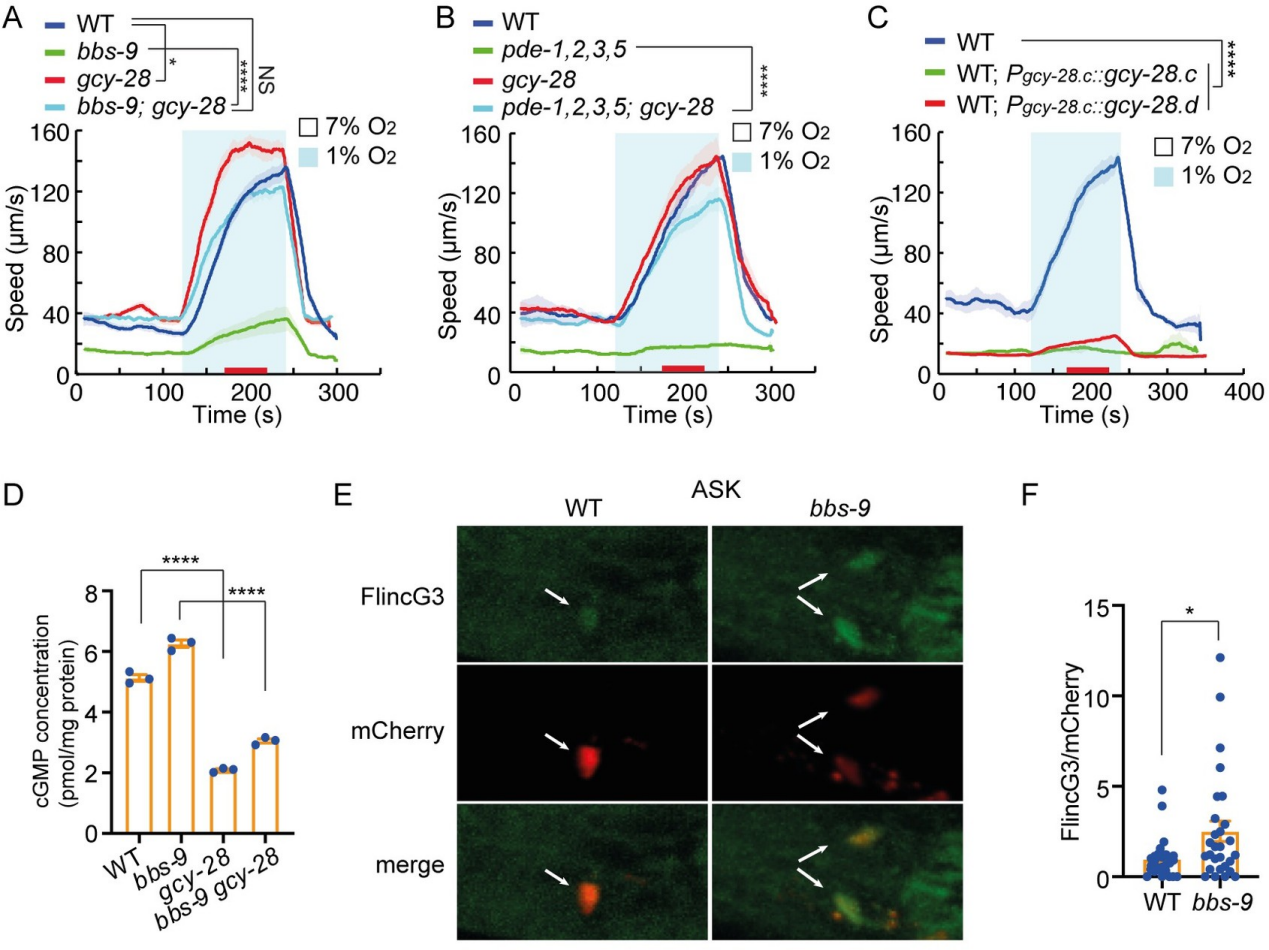
Disrupting GCY-28 suppresses the hypoxia response defects of *bbs-9* mutants. **(A–C)** Locomotory responses to 7% to 1% O_2_ stimuli for animals of the indicated genotypes: WT, *bbs-9(gk471)*, *gcy-28(yum32)*, and *bbs-9(gk471); gcy-28(yum32)* double mutants (A); WT, *pde-1(nj57); pde-2(tm3098); pde-3(nj59); pde-5(nj49)* quadruple mutants (*pde-1*, *2*, *3*, *5*), *gcy-28(yum39)*, and *gcy-28(yum39)*; *pde-1*, *2*, *3*, *5* (B); and *gcy-28*.*c* and *gcy-28*.*d* cDNA overexpressed from the *gcy-28*.*c* promoter in the WT background (C). **** = *p* < 0.0001, * = *p* < 0.05, NS = not significant. ANOVA, Tukey multiple comparison. **(D)** Total cGMP in worm lysates determined by cGMP enzyme immunoassay of indicated genotypes (*n* = 3): WT, *bbs-9(gk471)*, *gcy-28(yum32)*, and *bbs-9(gk471); gcy-28(yum32)*. **** = *p* < 0.0001. ANOVA, Tukey multiple comparison. **(E)** Representative images of the genetically encoded cGMP sensor FlincG3 expressed together with a mCherry marker in ASK neurons using the *sra-9* promoter and imaged in WT and *bbs-9(gk471)* mutants. Arrows point to neuronal cell bodies. **(F)** Quantitation of FlincG3 fluorescent signal intensity in ASK neurons normalized to mCherry fluorescence intensity. * = *p* < 0.05, *t* test. The underlying data can be found in S1 Data, and the source code can be found at https://github.com/wormtracker/zentracker. O_2_, oxygen; WT, wild-type.

cGMP is generated by guanylate cyclases. To identify guanylate cyclases whose activity suppresses hypoxia responses in *bbs-9* mutants, we studied hypoxia-evoked locomotory responses in guanylate cyclase mutants. None of the guanylate cyclase mutants was defective in their response to acute hypoxia, suggesting guanylate cyclases are dispensable for hypoxia sensing (S1 Table). Disrupting all 7 soluble guanylate cyclases encoded by the *C*. *elegans* genome increased the baseline speed of *bbs-9* mutants, but failed to restore their hypoxia-evoked behavioral response (S5A Fig). A survey of mutants defective in each of the 27 predicted receptor guanylate cyclases encoded in the *C*. *elegans* genome [44,45] identified several that partially suppressed the defective hypoxia response of *bbs-9* animals (S5B Fig). Strikingly, disrupting *gcy-28* alone was sufficient to restore hypoxia-evoked escape fully to *bbs-9* mutants (Figs 3A and S5B). Deleting *gcy-28* also robustly suppressed the hypoxia response defects of *pde-1*,*2*,*3*,*5* mutant animals (Fig 3B). Conversely, overexpressing either the *gcy-28*.*c* or the *gcy-28*.*d* splice isoforms from the *gcy-28*.*c* promoter effectively eliminated locomotory responses to hypoxia in wild-type animals (Fig 3C). By contrast, we did not observe any inhibitory effects when 2 other guanylate cyclases, *gcy-35* or *gcy-15*, were overexpressed (S5C and S5D Fig). Taken together, these data suggest that GCY-28 has a modulatory role in hypoxia-evoked behavioral responses.

We next asked whether the increased cGMP levels in *bbs-9* mutants were dependent on GCY-28. Total cGMP levels were markedly decreased in *gcy-28* mutants compared to wild type, and the elevated cGMP in *bbs-9* mutants was significantly reduced by removing *gcy-28* (Fig 3D). Our ELISA data suggest that GCY-28 is the major source of total cGMP in *C*. *elegans*, despite the 33 other guanylate cyclases encoded in the genome. This is consistent with the broad expression pattern of GCY-28; other guanylate cyclases are only expressed in a small number of neurons [45]. To visualize how cGMP levels in individual neurons were altered by loss of *bbs-9*, we used the FlincG3 cGMP sensor [46]. To help us avoid movement artifacts, we used pseudo-ratiometric imaging by co-expressing FlincG3 in the ASK, AFD, and AIA neurons with mCherry. The FlincG3 signal in each of these neurons was significantly higher in *bbs-9* mutants compared to wild type, indicating increased cGMP levels (Figs 3E, 3F, and S5E–S5H). These data suggest that *bbs* mutants inhibit the acute hypoxia response by promoting cGMP production.

### cGMP acts partly in AIA neurons to modulate acute responses to hypoxia

**Fig 4:**
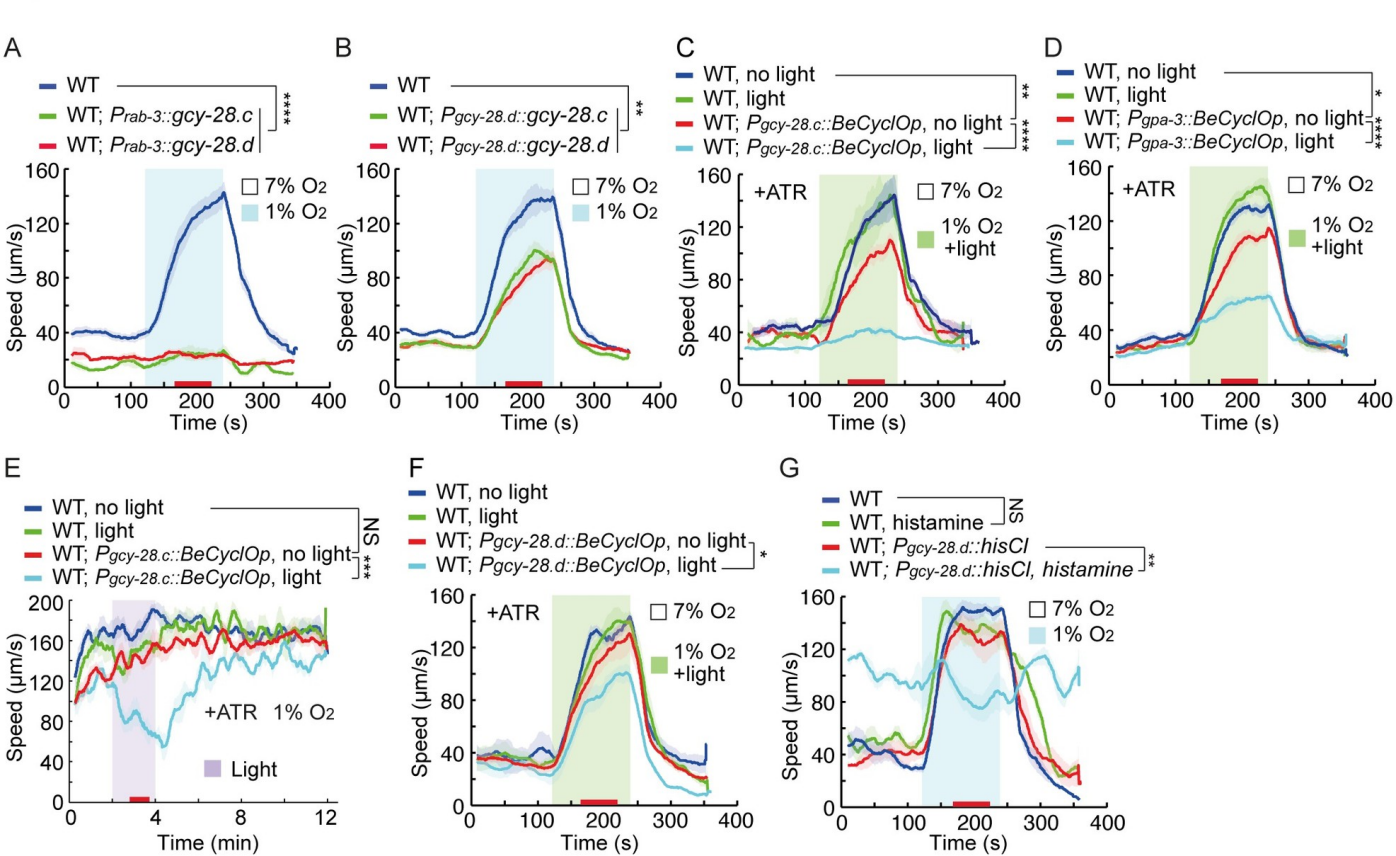
Acute cGMP production robustly inhibits locomotory responses to hypoxia. **(A, B)** Locomotory responses to 7% to 1% O_2_ stimuli of animals of indicated genotype: WT and WT expressing *gcy-28*.*c* or *gcy-28*.*d* cDNA pan-neuronally from the *rab-3* promoter (A); and WT and WT overexpressing *gcy-28*.*c* or *gcy-28*.*d* cDNA from the *gcy-28*.*d* promoter, which drives expression selectively in the AIA neurons (B). **** = *p* < 0.0001, and ** = *p* < 0.01. ANOVA, Tukey multiple comparison. **(C–F)** Optogenetic stimulation of cGMP production using BeCyclOp from the *gcy-28*.*c* (C and E), *gpa-3* (D) or *gcy-28*.*d* (AIA neurons) (F) promoters. L4 animals of each genotype were picked overnight to seeded plates containing ATR and assayed as adults. Blue light was delivered while O_2_ levels were switched from 7% to 1% (C, D, and F). In (E), animals were constantly exposed to 1% O_2_, and blue light was applied in a 2-minute time window, as indicated. **** = *p* < 0.0001, *** = *p* < 0.001, ** = *p* < 0.01, and * = *p* < 0.05, and NS = not significant. ANOVA, Tukey multiple comparison. **(G)** Locomotory responses to 7% to 1% O_2_ stimuli of animals with the indicated genotype: WT with or without 10mM histamine treatment, and WT expressing the *Drosophila* histamine-gated chloride channel HisCl1 in AIA neurons using the *gcy-28*.*d* promoter treated with or without 10 mM histamine. L4 animals of each genotype were transferred to plates containing 10 mM histamine for 10 minutes and assayed as adults on histamine containing plates. ** = *p* < 0.01, and NS = not significant. ANOVA, Tukey multiple comparison. The source code underlying behavioral data can be found at https://github.com/wormtracker/zentracker. O_2_, oxygen; WT, wild-type.

Since GCY-28 appears to be a highly active cyclase, we used it as a tool to explore where cGMP acts to inhibit acute responses to hypoxia. Increased expression of either the *gcy-28*.*c* or the *gcy-28*.*d* isoform from the *gcy-28*.*c* promoter potently inhibited locomotory responses to hypoxia (Fig 3C). However, these isoforms do not appear to be expressed in ASH neurons, and *gcy-28*.*c* displayed variable and inconsistent expression in ADL neurons (S6A Fig). The *gcy-28*.*c* isoform is widely expressed in neurons and other tissues, whereas the *gcy-28*.*d* isoform is expressed in a small subset of neurons including AIA interneurons [45,47]. Overexpressing *gcy-28*.*c* or *gcy-28*.*d* in neurons, but not in other tissues, eliminated acute responses to hypoxia (Figs 4A and S6B). To investigate further the anatomical focus of *gcy-28* action, we expressed *gcy-28*.*c* from various promoters, including *gpa-3p* (ADF, ADL, ASE, ASG, ASH, ASI, ASJ, ASK, AWA, AWC, and more broad expression was observed), *odr-3p* (AWA, AWB, AWC, ASH, and ADF), *ocr-2p* (ASH, ADL AWA, ADF, PHA, and PHB), *gpa-11p* (ADL and ASH), *odr-1p* (AWC, ASI, AWB, ASK, and ASJ), *sra-6p* (ASH and ASI), *srh-220p* (ADL), *ops-1p* (ASG), *flp-6p* (ASE), *flp-21p* (ASH, ADL, ASE, ASI, FLP, URA, RMG, MC, M2, and M4), *glr-1p* (AIB, AVA, AVD, AVE, AVJ, AVL, RIA, SMD, and many others), *npr-1p* (ASH, ASE, ASG, URX, AQR, RMG, IL2, ILQ, and many others), *nmr-1p* (AVA, AVD, AVE, AVG, PVC, RIM, and others), and *unc-25p* (DD, VD, RME, and others). Only overexpressing *gcy-28*.*c* from *gpa-3p* strongly inhibited acute hypoxia responses (S6C–S6I Fig). These observations suggested that multiple neurons are involved in responses to acute hypoxia, and the hypoxia sensor could be widely distributed. Notably, overexpressing *gcy-28*.*c* from the *gpa-11* promoter did not affect hypoxia-evoked locomotory responses, suggesting that cGMP overproduction in ADL and ASH, where BBS-9 and DYF-3 act, was not sufficient to inhibit rapid behavioral responses to hypoxia (S6E Fig). Interestingly, overexpressing *gcy-28*.*c* or *gcy-28*.*d* only in AIA neurons, using the *gcy-28*.*d* promoter, partially inhibited locomotory responses to acute hypoxia (Fig 4B), indicating that elevating cGMP production in AIA can inihibit responses to 1% O_2_ stimulation.

To further investigate critical sites of cGMP signaling, we optogenetically manipulated intracellular cGMP levels using a guanylate cyclase rhodopsin from *Blastocladiella emersonii*, BeCyclOp, that enables rapid light-triggered increases in cGMP production [43]. Without blue light, and in the presence of the cofactor all *trans*-retinal (ATR), transgenic animals expressing BeCyclOp from the *gcy-28*.*c* (Fig 4C), or *gpa-3* (Fig 4D) promoters responded strongly to 1% O_2_, although a weak suppression was observed. By contrast, exposing these animals to blue light and 1% O_2_ in the presence of ATR effectively suppressed acute hypoxia responses (Fig 4C and 4D). To probe the temporal dynamics and reversibility of this manipulation, we kept animals in 1% O_2_ and waited until the animals’ speed plateaued, at which point light stimulation of BeCyclOp triggered an abrupt slowing followed by resumption of rapid movement when light was switched off again (Fig 4E). In the absence of ATR, responses to 1% O_2_ were unaffected in animals expressing BeCyclOp (S7A and S7B Fig), supporting the notion that heightened intracellular cGMP inhibits acute hypoxia responses. Intriguingly, BeCyclOp-mediated cGMP production in AIA neurons, but not in ADL and ASH neurons, significantly inhibited locomotory responses to acute hypoxia (Figs 4F and S7C), consistent with the *gcy-28* overexpression data (Fig 4B). To confirm the role of AIA neurons in acute hypoxia responses, we inhibited AIA neurons using a histamine-gated chloride channel (HisCl1) [48]. Inhibition of AIA neurons increased the basal speed at 7% O_2_ while completely eliminating responses to 1% O_2_ (Fig 4G), suggesting that AIA neurons are an important element of the circuit mediating responses to acute hypoxia.

### BBSome acts in ciliated neurons to indirectly modulate cGMP production in AIA interneurons

The fact that cGMP levels are elevated in *bbs-9* mutants, and that GCY-28 is a major contributor to cGMP production, prompted us to speculate that GCY-28 expression or localization might be altered in *bbs-9* mutants. However, we observed reduced instead of increased expression of GFP-tagged GCY-28.C and GCY-28.D in *bbs-9* mutants, and no obvious change in the subcellular localization of GCY-28-GFP when expressed either from a single copy MosSCI insertion (GCY-28.C) or from extrachromosomal arrays (GCY-28.D) (S8A and S8B Fig). It is possible that GCY-28 activity, not expression, is affected by loss of the BBSome. However, since GCY-28 is not localized to the cilia [47] and its main action site does not overlap with BBSome expressing neurons, its activity is unlikely to be directly targeted by the BBSome. Moreover, *bbs-9* mutants showed increased cGMP production even in the absence of GCY-28 (Fig 3D, columns 3 and 4), suggesting that other guanylate cyclases may be activated in a *bbs-9* mutant background. Supporting this, mutations in several other guanylate cyclase genes besides *gcy-28* partially suppressed the hypoxia response defects of *bbs-9* mutants (S5B Fig). Taken together, we suspect that GCY-28 may not be directly involved in abnormal cGMP generation in *bbs-9* mutants. However, because GCY-28 is a major guanylate cyclase in cGMP production (Fig 3D), loss of GCY-28 could effectively neutralize any increases in cGMP generated by other guanylate cyclases in *bbs-9* mutants, thereby reversing their inhibition of acute responses to hypoxia.

**Fig 5:**
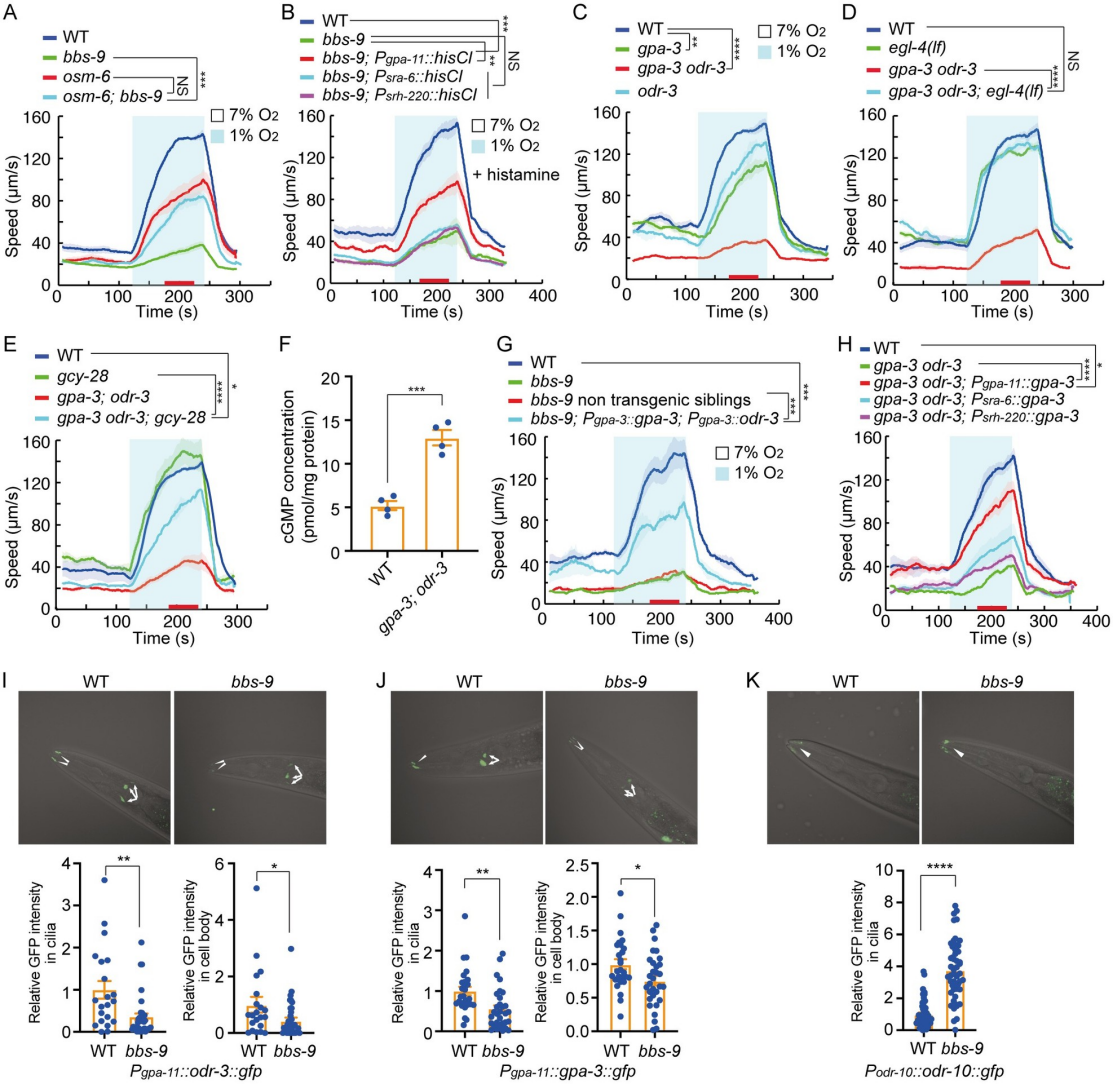
G protein signaling regulates acute responses to hypoxia. **(A)** Locomotory responses to 7% to 1% O_2_ stimuli of animals with indicated genotypes: WT, *bbs-9(gk471)*, *osm-6(p811)*, and *bbs-9(gk471)*; *osm-6(p811)* double mutants. *** = *p < 0*.*001*, NS = not significant. ANOVA, Tukey multiple comparison. **(B)** Locomotory responses to 7% to 1% O_2_ stimuli of animals treated with 10mM histamine: WT, *bbs-9(gk471)*, *bbs-9(gk471)* expressing HisCl1 from *sra-6* (ASH), *srh-220* (ADL) and *gpa-11* (ASH and ADL) promoters. *** = *p* < 0.001, ** = *p* < 0.01, and NS = not significant. ANOVA, Tukey multiple comparison. **(C–E)** Locomotory responses to 7% to 1% O_2_ stimuli of animals with indicated genotypes: WT, *gpa-3(pk35)*, *odr-3(n1605)*, and *gpa-3(pk35) odr-3(n1605)* (C); WT, *egl-4(n478)*, *gpa-3(pk35) odr-3(n1605)*, and *gpa-3(pk35) odr-3(n1605)*; *egl-4(n478)* triple mutants (D); and WT, *gcy-28(yum32)*, *gpa-3(pk35) odr-3(n1605)*, and *gpa-3(pk35) odr-3(n1605)*; *gcy-28(yum32)* triple mutants (E). **** = *p* < 0.0001, ** = *p* < 0.01, and NS = not significant. ANOVA, Tukey multiple comparison. **(F)** Total cGMP in worm lysates determined by cGMP enzyme immunoassay of indicated genotypes: WT and *gpa-3(pk35) odr-3(n1605)* double mutants. *** = *p* < 0.001. *t* test. **(G and H)** Locomotory responses to 7% to 1% O_2_ stimuli of animals with indicated genotypes: WT, *bbs-9(gk471)*, transgenic *bbs-9(gk471)* expressing *gpa-3* and *odr-3* transgenes from the *gpa-3* promoter and their non-transgenic *bbs-9(gk471)* siblings (G); WT, *gpa-3(pk35) odr-3(n1605)*, *gpa-3(pk35) odr-3(n1605)* expressing *gpa-3* cDNA in ADL and ASH neurons (*gpa-11p*), in ASH (*sra-6p*) or ADL (*srh-220p*) alone (H). **** = *p* < 0.0001, *** = *p* < 0.001, and * = *p* < 0.05. ANOVA, Tukey multiple comparison. **(I and J)** Representative images (upper panel) and quantification (lower panel) of GFP-tagged markers in the cilia and neuronal cell bodies of WT and *bbs-9(gk471)* animals. Arrowheads point to cilia; arrows indicate neuronal cell bodies. The plots quantify GFP in cilia (left) and cell bodies (right). The average values in WT were arbitrarily set to 1, and the GFP signal in *bbs-9(gk471)* mutants normalized to WT. (I), ODR-3-GFP expressed from the *gpa-11* promoter, ** = *p* < 0.01, * = *p* < 0.05, *t* test. (J), GPA-3-GFP expressed from the *gpa-11* promoter, ** = *p* < 0.01, * = *p* < 0.05, *t* test. **(K)** ODR-10-GFP expression in the cilia of AWA neurons. Arrowheads indicate the AWA cilia. The GFP intensity of WT was arbitrarily set to 1, and the GFP signal of *bbs-9(gk471)* was normalized to WT. **** = *p* < 0.0001, *t* test. The underlying data can be found in S1 Data, and the source code can be found at https://github.com/wormtracker/zentracker. O_2_, oxygen; WT, wild-type.

How does the BBSome, which acts in ciliated neurons, modulate cGMP production in downstream circuits such as the AIA neurons? Disrupting *daf-19*, which encodes an RFX transcription factor essential for ciliogenesis [49], attenuated but did not abolish hypoxia responses (S1 Table). This makes it unlikely that loss of acute responses to hypoxia in *bbs-9* mutants reflects loss of cilia function. An alternative hypothesis, supported by previous studies, is that *bbs* mutants have acquired ectopic signaling capability, perhaps because they accumulate signaling components in their cilia due to defective retrograde traffic [50–54]. To test this, we asked if blocking accumulation of signaling molecules in cilia rescues the hypoxia response defects of *bbs* mutants. Indeed, eliminating the core IFT component OSM-6 suppressed the hypoxia response defects of *bbs-9* to the levels found in *osm-6* single mutants (Fig 5A).

Since *bbs-9* hypoxia response defects were rescued by expressing *bbs-9* cDNA in ADL and ASH neurons, we surmised that signals from these 2 neurons could be heightened in *bbs-9* mutants. If this was the case, silencing ADL and ASH neurons should suppress the defects of *bbs-9* mutants. Indeed, simultaneously but not individually inhibiting ADL and ASH neurons using the histamine-gated chloride channel HisCl1 partially restored locomotory responses to *bbs-9* mutants (Fig 5B). By contrast, inhibiting or ablating these 2 neurons in the wild-type background did not affect responses to acute hypoxia (S8C and S8D Fig). These data suggest that abnormal signaling from ADL and ASH in *bbs-9* mutants is at least partially responsible for loss of acute hypoxia responses in these animals.

### BBSome modulates cGMP production through GPCR signaling

We next probed the nature of the ectopic signaling in *bbs* mutants in ADL and ASH neurons. Mutants lacking the Gi/Go-like protein GPA-3, similar to *bbs* mutants, have high cGMP levels [55]. Disrupting *gpa-3* weakly reduced the locomotory response to hypoxia (Fig 5C). By assaying mutants in other Gα subunits, we found that simultaneously disrupting *gpa-3* and *odr-3* strongly attenuated responses to acute hypoxia (Fig 5C). Mutations in *gcy-28* or *egl-4* suppressed this defect (Fig 5D and 5E) and, consistent with this, *gpa-3 odr-3* double mutants showed significantly elevated cGMP (Fig 5F), suggesting that GPA-3 and ODR-3 redundantly inhibit cGMP production. We examined whether the BBSome and G proteins act in parallel or in the same pathway to modulate GCY-28 signaling. We found that simultaneously overexpressing *gpa-3* and *odr-3* restored hypoxia-evoked behavioral responses to *bbs-9* mutants, suggesting that in genetic terms the BBSome acts upstream of G protein signaling to promote acute hypoxia responses (Fig 5G). We next examined if GPA-3 and ODR-3 act in the same neurons as BBSome. The behavioral defects of *gpa-3 odr-3* double mutants were partially rescued by expressing *gpa-3* under the *gpa-11* promoter (Fig 5H). These observations suggest that G proteins, similar to the BBSome, act in ADL and ASH neurons to modulate locomotory responses to hypoxia. Since both GPA-3 and ODR-3 are localized to sensory cilia, we tested whether this localization was affected in *bbs-9* mutants. The expression of both G proteins was significantly decreased in *bbs-9* mutants compared to wild type, both in neuronal cell bodies and in cilia of ADL and ASH neurons (Fig 5I and 5J). ODR-3 expression in the cilia of AWA neurons showed the same tendency (S8E Fig). By contrast, and consistent with previous observations, levels of the ODR-10 olfactory GPCR in cilia increased >2-fold in *bbs-9* mutants (Fig 5K) [53]. Together, these data suggest that disrupting the BBSome alters G protein localization and signaling in ciliated neurons to modulate cGMP production in downstream circuits.

### Abnormal neuroendocrine secretion disrupts acute hypoxia responses

**Fig 6:**
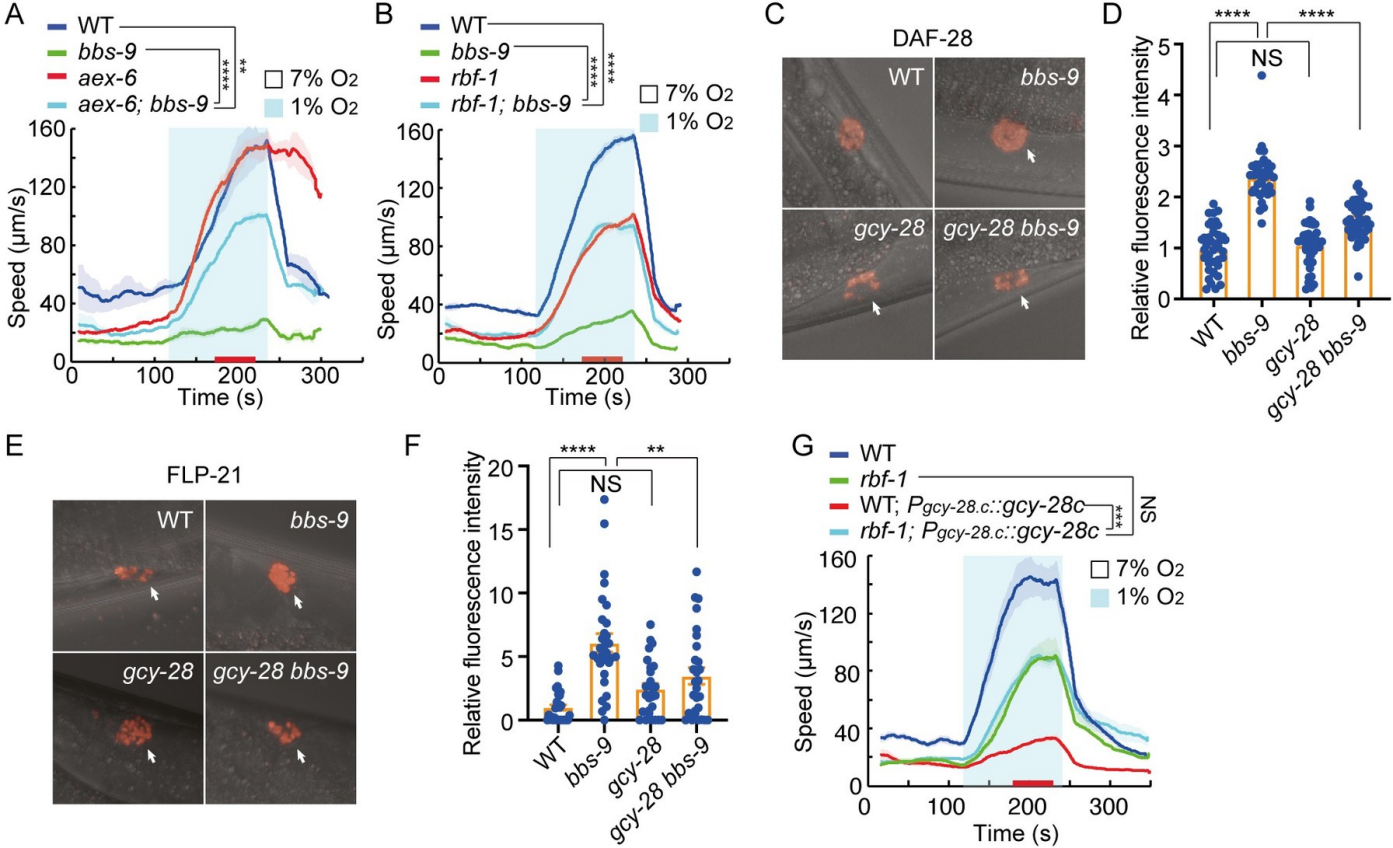
Increased neuropeptide secretion inhibits acute hypoxia responses. **(A and B)** Locomotory responses to 7% to 1% O_2_ stimuli for indicated genotypes: WT, *bbs-9(gk471)*, *aex-6(sa24)*, and *bbs-9(gk471); aex-6(sa24)* double mutants in (A); and WT, *bbs-9(gk471)*, *rbf-1(js232)*, and *bbs-9(gk471); rbf-1(js232)* double mutants in (B). **** = *p* < 0.0001, ** = *p* < 0.01, ANOVA, Tukey multiple comparison. **(C)** Coelomocyte accumulation of DAF-28-mCherry secreted from ADL neurons in WT, *bbs-9(gk471)*, *gcy-28(yum32)*, and *bbs-9(gk471) gcy-28(yum32)* double mutants. Arrows indicate posterior coelomocytes. **(D)** Quantification of DAF-28-mCherry fluorescent intensity in one of the posterior coelomocytes. The mCherry intensity in WT (*n* = 40) was arbitrarily set to 1, and the mCherry signal in *bbs-9(gk471)* (*n* = 36), *gcy-28(yum32)* (*n* = 40), and *bbs-9(gk471) gcy-28(yum32)* (*n* = 40) mutants was normalized to WT. **** = *p* < 0.0001, NS = not significant. ANOVA, Tukey multiple comparison. **(E)** Coelomocyte accumulation of FLP-21-mCherry in WT, *bbs-9(gk471)*, *gcy-28(yum32)*, and *bbs-9(gk471) gcy-28(yum32)* double mutants. Arrows indicate posterior coelomocytes. **(F)** Quantification of FLP-21-mCherry fluorescent intensity in one of the coelomocytes. The mCherry intensity in WT (*n* = 30) was arbitrarily set to 1, and the mCherry signal in *bbs-9(gk471)* (*n* = 30), *gcy-28(yum32)* (*n* = 27), and *bbs-9(gk471) gcy-28(yum32)* (*n* = 28) mutants was normalized to WT. **** = *p* < 0.0001, ** = *p* < 0.01, and NS = not significant. ANOVA, Tukey multiple comparison. **(G)** Locomotory responses to 7% to 1% O_2_ stimuli for animals of the indicated genotype: WT, *rbf-1(js232)* and *gcy-28*.*c* overexpression from the *gcy-28*.*c* promoter in WT and *rbf-1(js232)* background. *** = *p* < 0.001, NS = not significant, ANOVA, Tukey multiple comparison. The underlying data can be found in S1 Data, and the source code can be found at https://github.com/wormtracker/zentracker. O_2_, oxygen; WT, wild-type.

BBSome mutants have been shown to enhance neuropeptide secretion from ADL neurons [51]. This increased neurosecretion can be abrogated by disrupting *aex-6*, the *C*. *elegans* ortholog of human RAB27, or by mutations in the RAB27 regulator RBF-1. These findings prompted us to ask if increased cGMP levels inhibit acute responses to hypoxia by promoting neurotransmission. Consistent with this, deleting *aex-6* or *rbf-1* restored acute hypoxia responses to *bbs-9* mutants (Fig 6A and 6B). Moreover, enhanced secretion of the insulin DAF-28 and the FMRF-like peptide FLP-21 in *bbs-9* mutants was suppressed by a mutation in *gcy-28*, although loss of *gcy-28* did not alter release of these peptides in WT (Fig 6C–6F). In addition, the defective acute hypoxia responses in animals overexpressing *gcy-28*.*c* under its endogenous promoter were suppressed by mutations in *rbf-1* (Fig 6G). These observations suggest that increased cGMP signaling enhances neuroendocrine secretion, which, in turn, inhibits behavioral responses to 1% O_2_.

### Hypoxia-induced neuronal inhibition

**Fig 7:**
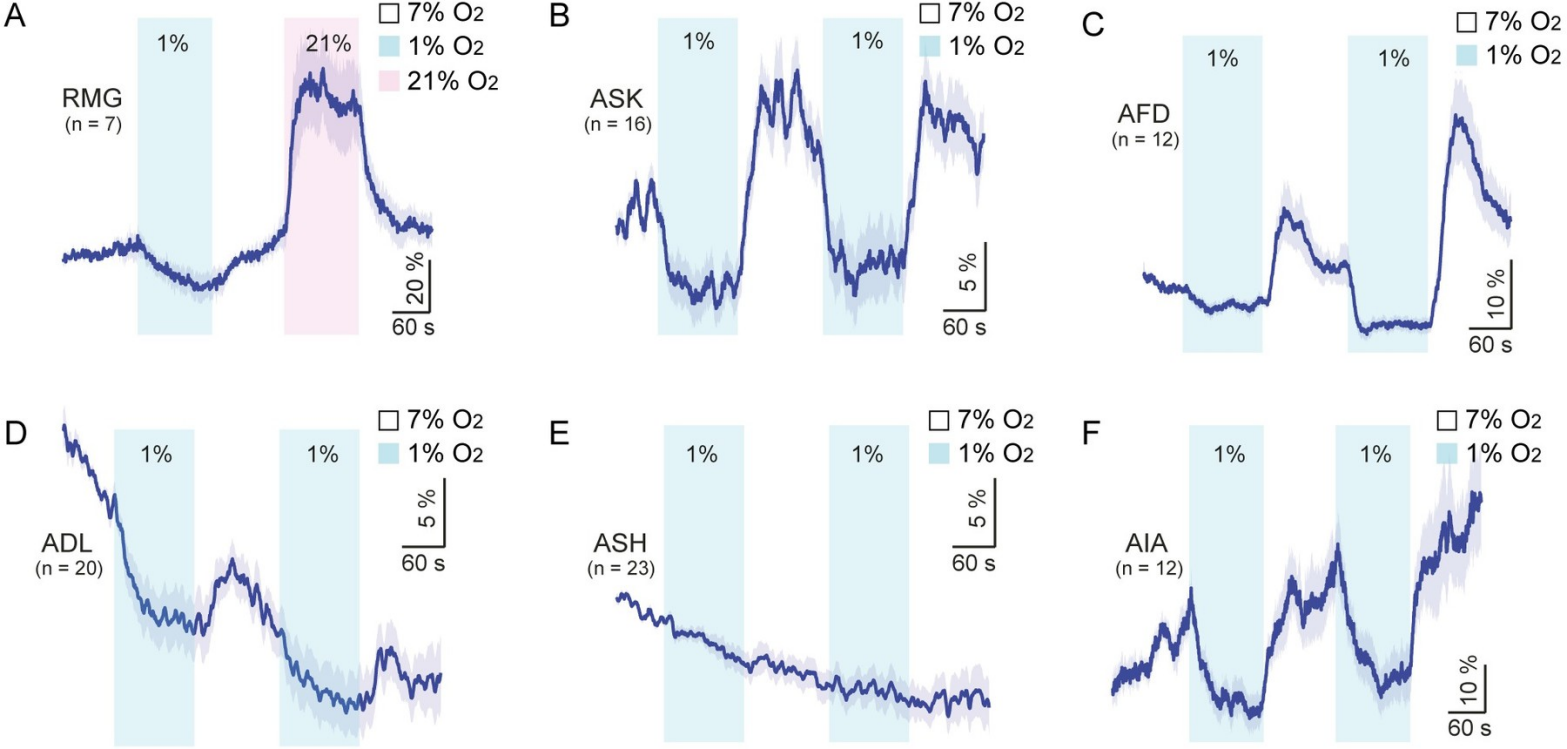
Hypoxia-evoked Ca^2+^ responses in different neurons. **(A)** Ca^2+^ responses evoked by 1% and 21% O_2_ in RMG interneurons. Ca^2+^ responses were measured using YC2.60. **(B–F)** Hypoxia-evoked Ca^2+^ responses in the ASK, AFD, ADL, and ASH sensory neurons, and in the AIA interneurons. Ca^2+^ responses were measured using YC3.60 sensors in these neurons. The source code underlying calcium imaging analysis can be found at https://github.com/neuronanalyser/neuronanalyser. O_2_, oxygen.

Our data suggest that despite the similarity of the behavioral responses, the neural circuits mediating escape from 1% and 21% O_2_ are distinct. Whereas 21% O_2_ evokes a tonic rise in Ca^2+^ in RMG interneurons, which is sufficient to drive rapid movement [14,16], 1% O_2_ evoked a decrease of Ca^2+^ levels in RMG (Fig 7A). We sought neurons that respond to hypoxia, using the ratiometric Ca^2+^ sensor YC3.60. None of the amphid neurons we imaged showed a hypoxia-evoked rise in Ca^2+^. However, we observed stimulus-evoked decreases of Ca^2+^ in ASK, AFD, and ADL neurons, suggesting neuronal hyperpolarization, which was frequently followed by a poststimulus Ca^2+^ “rebound” (Fig 7B–7D). We observed a rapid bleaching of the calcium sensor when imaging ADL neurons, which made the hypoxia-evoked Ca^2+^ decrease less obvious in these neurons. However, a clear rise of Ca^2+^ at 7% O_2_ suggests that there is an intrinsic hypoxia response in ADL neurons. Ca^2+^ transients evoked by hypoxia were not observed in ASH neurons (Fig 7E), suggesting that hypoxia did not trigger a general decrease of Ca^2+^ in neurons. This is consistent with our cell ablation data, suggesting that ASH neurons are not involved in the sensing step of hypoxia. To probe the circuitry underlying hypoxia responses further, we monitored Ca^2+^ responses evoked in the amphid interneurons AIB, AIY, and AIA, by shifting ambient O_2_ from 7% to 1%. We detected clear responses only in the AIA neurons, which also appeared to be inhibited by a 1% O_2_ stimulus (Fig 7F). This observation is consistent with our finding that stimulating cGMP production specifically in AIA neurons inhibits hypoxia responses. However, we do not exclude the possibility that the Ca^2+^ responses are indirect consequences of hypoxia such as altered pH values and ATP production.

### Defects in the mitochondrial electron transport chain disrupt acute hypoxia responses

**Fig 8:**
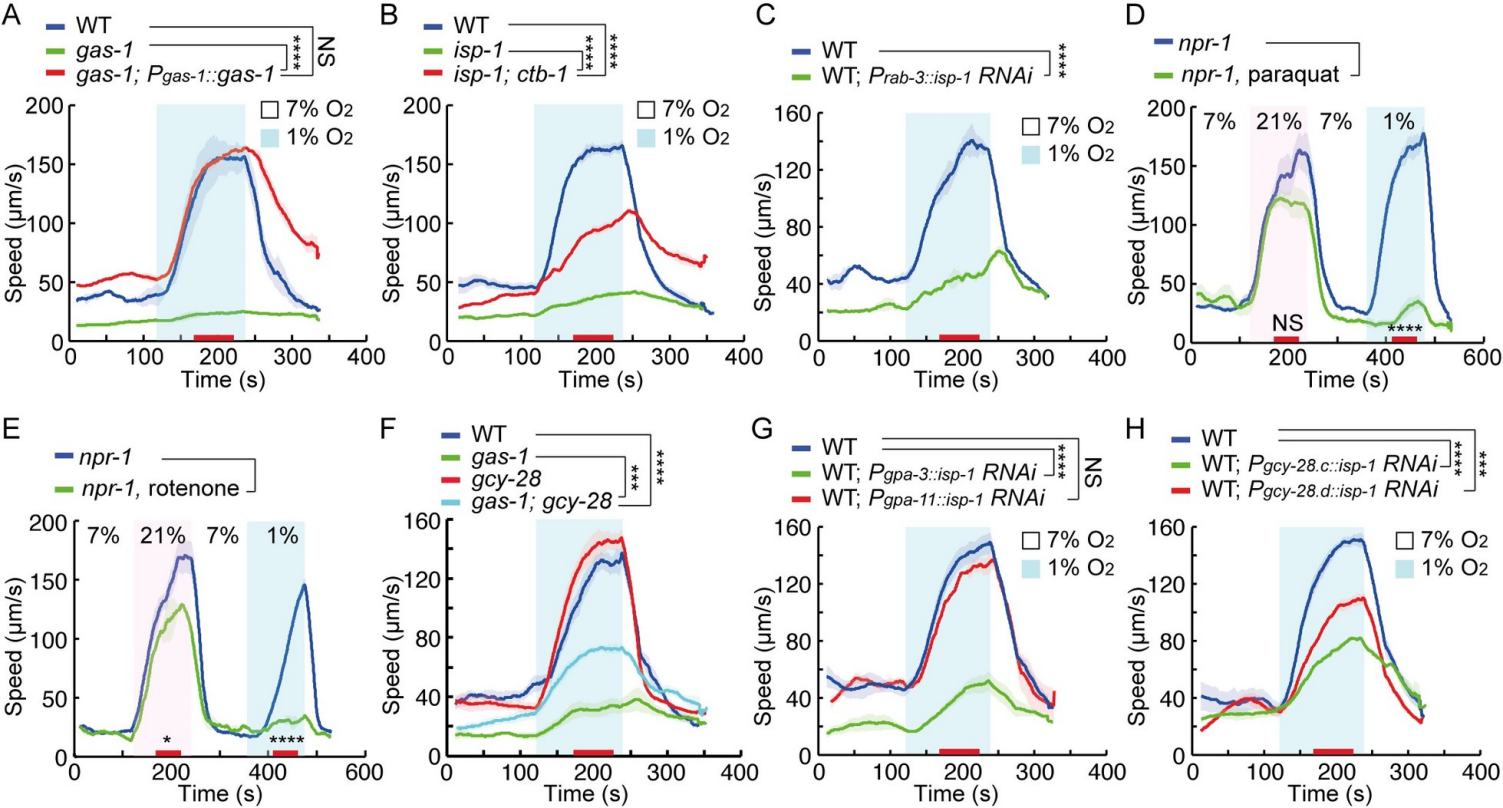
Mitochondrial malfunction leads to defects in acute hypoxia responses. **(A)** Locomotory responses to 7% to 1% O_2_ stimuli of animals of indicated genotypes: WT, *gas-1(fc21)* and transgenic *gas-1(fc21)* mutants expressing *gas-1* cDNA from the *gas-1* promoter. **** = *p* < 0.0001, NS = not significant. ANOVA, Tukey multiple comparison. **(B)** Locomotory responses of animals of indicated genotypes: WT, *isp-1(qm150)* and *isp-1(qm150); ctb-1(qm189)* animals, to 7% to 1% O_2_ switches. **** = *p* < 0.0001, ANOVA, Tukey multiple comparison. **(C)** Locomotory responses to 7% to 1% O_2_ stimuli of animals of indicated genotypes: WT, and WT expressing *isp-1* RNAi constructs in the nervous system from the *rab-3* promoter. **** = *p* < 0.0001. Mann–Whitney U test. **(D and E)** Locomotory responses of *npr-1(ad609)* mutants to 21% (pink) and 1% O_2_ (cyan) after treatment with either 200mM paraquat for 10 minutes (D), or 20μM rotenone for 2 hours (E). NS = not significant (21% O_2_), * = *p* < 0.05 (21% O_2_), **** = *p* < 0.0001 (1% O_2_). Mann–Whitney U test. **(F)** Locomotory responses to 7% to 1% O_2_ stimuli for WT, *gas-1(fc21)*, *gcy-28(yum28)*, and *gas-1(fc21); gcy-28(yum28)* animals. **** = *p* < 0.0001, *** = *p* < 0.001. ANOVA, Tukey multiple comparison. **(G)** Locomotory responses to 7% to 1% O_2_ stimuli of animals of indicated genotypes: WT, WT expressing *isp-1* RNAi constructs from the *gpa-3* (multiple neurons) or *gpa-11* (ADL and ASH) promoters. **** = *p* < 0.0001, NS = not significant. ANOVA, Tukey multiple comparison. **(H)** Locomotory responses to 7% to 1% O_2_ stimuli of animals of indicated genotypes: WT, WT expressing *isp-1* RNAi constructs under *gcy-28*.*c* (multiple neurons) or *gcy-28*.*d* (AIA neurons) promoters. **** = *p* < 0.0001, *** = *p* < 0.001. ANOVA, Tukey multiple comparison. The underlying data can be found in S1 Data, and the source code can be found at https://github.com/wormtracker/zentracker. RNAi, RNA interference; O_2_, oxygen; WT, wild-type.

Assaying mutants with compromised mitochondrial function identified 2 additional components, GAS-1 and ISP-1, required for acute responses to hypoxia (S1 Table). *gas-1* is the *C*. *elegans* ortholog of *Ndufs2*, which contributes to the ubiquinone binding site of MCI. Previous work has suggested that NDUFS2 is required for acute hypoxia sensing in the carotid body [6]. In *C*. *elegans*, mutations in *gas-1* entirely eliminated acute locomotory responses to hypoxia, and this defect could be rescued by expressing *gas-1* cDNA under its endogenous promoter (Fig 8A). *isp-1* encodes *C*. *elegans* ortholog of Rieske protein, the iron sulfur protein of MCIII. Disrupting Rieske protein in pulmonary vascular smooth muscle cells inhibits acute responses to hypoxia [56]. We found that *isp-1* mutants in *C*. *elegans* were strongly defective in locomotory responses to acute hypoxia (Fig 8B). However, repairing the point mutation of *isp-1(qm150)* by CRISPR, or adding fosmid transgenes bearing a wild-type *isp-1* gene, only partially rescued the hypoxia response phenotype, suggesting that a background mutation enhanced the hypoxia response defect in this *isp-1* mutant strain (S9A and S9B Fig). To further evaluate the role of ISP-1 in behavioral responses to hypoxia, we expressed sense and antisense RNAi constructs to silence *isp-1* expression [35]. RNAi knockdown of *isp-1* using the *isp-1* or *myo-2* promoters led to lethality, showing the efficacy of the RNAi strategy. RNAi knockdown of *isp-1* in the nervous system, but not in other tissues, inhibited escape from 1% O_2_ without disrupting responses to 21% O_2_ (Figs 8C and S9C–S9E). Thus, ISP-1 activity in neurons specifically promotes the acute hypoxia response.

MCI and MCIII are major production sites of ROS. The primary ROS generated in mitochondria is superoxide, which is detoxified to H_2_O_2_ by mitochondrial superoxide dismutase (SOD). We measured in vivo ROS production by staining worms with dihydroethidium (DHE). Both *gas-1* and *isp-1* mutants, but not *bbs-9*, *dyf-3* and *pde-1*,*2*,*3*,*5* quadruple mutants, had significantly increased ROS levels (S9F and S9G Fig). Mitochondrial superoxide is selectively increased in *isp-1* mutants [57]. To test whether superoxide levels help signal acute hypoxia, we examined acute hypoxia responses in mitochondrial SOD mutants. Loss of *sod-2* partially inhibited acute responses to hypoxia, and a stronger inhibition was observed when all SOD genes were disrupted (S9H Fig). These data prompted us to test if increasing mitochondrial ROS production using pro-oxidant drugs altered acute hypoxia responses. Treatment with paraquat and juglone, which induce mitochondrial superoxide generation, blocked locomotory speed increase to 1% O_2_ while responses to 21% O_2_ were affected to a much lesser extent (Figs 8D and S9I). As paraquat and juglone may have other effects, we explored other superoxide inducing drugs. Rotenone is a strong inducer of superoxide [58–61] and is known to block acute hypoxia sensing by peripheral chemoreceptors in mammals [62–64]. Treatment with rotenone abolished the hypoxia-evoked increase in speed but had only a mild effect on the response to 21% O_2_ (Fig 8E). These observations suggest persistently high ROS levels inhibit *C*. *elegans’* response to acute hypoxia.

The inhibition of hypoxia-evoked locomotory responses by high cGMP promoted us to ask if high mitochondrial ROS levels stimulate cGMP production. We found that both *gas-1* and *isp-1* mutants showed increased total cGMP (S10A Fig). Moreover, treatment with paraquat rapidly stimulated cGMP production in wild-type animals (S10B Fig). Pro-oxidant induced cGMP production was blocked by including the antioxidant N-acetyl cysteine (NAC) during paraquat exposure and was absent in GCY-28 mutants (S10C Fig), suggesting the ROS induced cGMP generation is GCY-28 dependent. The behavioral defects of *gas-1* mutants were partially suppressed in a double mutant with *gcy-28* (Fig 8F), suggesting that increased cGMP contributes to the phenotype of mitochondrial mutants. To explore if ROS acts in the same neurons as cGMP, we selectively inactivated ISP-1 by RNAi in different neurons. Expressing RNAi constructs from the *gpa-3* promoter robustly inhibited behavioral responses to 1% but not to 21% O_2_ (Figs 8G and S10D). Potent inhibition was also observed by using the *gcy-28*.*c* promoter to drive the RNAi constructs (Fig 8H). In addition, silencing *isp-1* in AIA neurons, but not in ADL and ASH, significantly reduced the locomotory arousal evoked by 1% O_2_, suggesting that ROS and cGMP act in the same neurons (Fig 8G and 8H). These results suggest mitochondrial ROS can inhibit acute hypoxia responses by stimulating cGMP signaling in a GCY-28–dependent manner.

## Discussion

Here, we characterize the acute locomotory responses of *C*. *elegans* to a hypoxic (1% O_2_) environment. A rapid decrease in O_2_ to hypoxic (1%) levels triggers a bout of reorientation events, as animals seek to avoid hypoxia, followed by a large increase in locomotory activity that is sustained as long as animals remain in hypoxia. These behavioral responses to hypoxia occur within seconds and are remarkably similar to those evoked by 21% O_2_, a signal of surface exposure that is also a noxious cue for *C*. *elegans*. Notwithstanding the similar responses, the known O_2_-sensing neurons AQR, PQR, URX, and BAG, and the downstream interneurons RMG, which mediate escape from 21% O_2_, are not required for escape from hypoxia. Moreover, disrupting all 7 *C*. *elegans* soluble guanylate cyclases, GCY-31–37, which are thought to bind O_2_ at their heme co-factor, or the cGMP-gated channels TAX-4/TAX-2 that transduce O_2_-evoked cGMP signals to drive escape from 21% O_2_, does not disrupt hypoxia-evoked escape responses. These results suggest the neuronal and molecular substrates underlying responses to 1% and 21% O_2_ are different. They also suggest that the hypoxia-sensing mechanism mediating *C*. *elegans* escape from 1% O_2_ differs from that used by *Drosophila* larvae, which depends on soluble guanylate cyclases [65].

*C*. *elegans’* acute responses to hypoxia are, however, inhibited by increased cGMP signaling through PKG. Most notably, rapid generation of cGMP using optogenetics is sufficient to prevent *C*. *elegans* arousal in response to 1% O_2_. These observations raise the possibility that *C*. *elegans* responds to acute hypoxia by directly and rapidly inhibiting cGMP production. However, other observations argue against this hypothesis. If the locomotory responses to hypoxia were triggered by a decrease in cGMP, we might expect that mutants defective in cGMP signaling would display constitutive high locomotory activity at preferred O_2_ levels, as animals tonically signal that they are in constant hypoxia. However, both *egl-4* and *gcy-28* mutants show normal locomotory activity at 7% O_2_, suggesting that reduced cGMP signaling is not sufficient to evoke escape behavior. Moreover, the fact that none of the guanylate cyclase mutants show defective responses to hypoxia suggests GCYs are not essential for sensing acute hypoxia, although we cannot rule out redundancy between cyclases. Thus, we favor a model in which cGMP is a modulator rather than the primary signal of acute hypoxia responses. Our observation is consistent with the inhibitory role of cGMP in acute hypoxia responses in mammalian chemosensory organs [9–12]. It also echoes a gasotransmitter model, in which high cGMP and high PKG activity inhibit the synthesis of H_2_S to prevent acute responses to hypoxia [66]. All these suggest a conserved role of cGMP/PKG in modulating acute hypoxia sensing.

In *C*. *elegans* many cGMP signaling molecules, e.g., most guanylate cyclases, are concentrated in sensory cilia. However, the BBSome does not appear to directly regulate traffic of GCY-28, a guanylate cyclase that contributes disproportionately to cGMP production in both wild-type and *bbs* mutants. Unlike many other guanylate cylases, GCY-28 does not localize to cilia [47] and its expression is not increased, nor its localization altered, in *bbs* mutants (S8A and S8B Fig). The defects of *bbs-9* mutants are rescued by expressing *bbs-9* cDNA in ADL and ASH neurons that do not reliably express GCY-28. Moreover, increasing cGMP production selectively in ADL and ASH, either optogenetically or by overexpressing *gcy-28*, is not sufficient to inhibit hypoxia-evoked locomotory responses. Together, these data suggest that GCY-28 and the BBSome act in different neurons to regulate hypoxia responses. The BBSome may instead modulate acute responses to hypoxia by regulating the function of the GPA-3 and ODR-3 G proteins. Supporting this, GPA-3 and ODR-3 act in the same neurons as BBSome components to promote escape from hypoxia. Moreover, the expression of GPA-3 and ODR-3 is down-regulated in ADL and ASH neurons in *bbs-9* mutants (Fig 5I and 5J), and overexpressing GPA-3 and ODR-3 partly restores hypoxia responses to *bbs-9* mutants. The phenotypic suppression of G protein mutants by disrupting cGMP signaling places cGMP further downstream of, or possibly in parallel to, ODR-3 and GPA-3 G protein signaling. Consistent with this, cGMP production in several neurons, including AIA interneurons, can inhibit acute hypoxia responses. How signals from ADL and ASH neurons interact with signals from cGMP producing neurons to regulate hypoxia responses is unclear.

Mitochondria have historically been regarded as good candidates for harboring the mechanism for acute hypoxia sensing. In glomus cells of the carotid body, increased ROS generation is thought to regulate opening of adjacent K^+^ channels in the plasma membrane, thereby signaling drops in O_2_ tension and establishing an integrative mitochondria-to-membrane signaling model of acute hypoxia sensing [67–69]. Precisely how mitochondrial ROS alters the open probability of K^+^ channels in the plasma membrane remains to be elucidated. In both carotid body and smooth muscle cells of pulmonary arteries, high ROS levels in MCI and MCIII mutants prevent further ROS increase upon acute hypoxia, thereby inhibiting the responses to the drop of O_2_ tension [1,6,8]. Our work shows that similar manipulations in *C*. *elegans* eliminate locomotory responses to hypoxia, suggesting that molecular mechanism of acute hypoxia sensing may be conserved from *C*. *elegans* to mammals. We also provide insight into how increased ROS levels inhibit the responses to acute hypoxia. Both genetic and pharmacological perturbations of ROS production increase intracellular cGMP levels, raising the possibility that ROS inhibits acute hypoxia sensing by stimulating cGMP production. This is supported by the fact that ROS act in the same cells as cGMP, and the defects of *gas-1* mutants in acute responses to hypoxia are partially suppressed by *gcy-28* mutants. These data suggest a mechanism by which increased ROS can inhibit acute hypoxia sensing.

**Fig 9:**
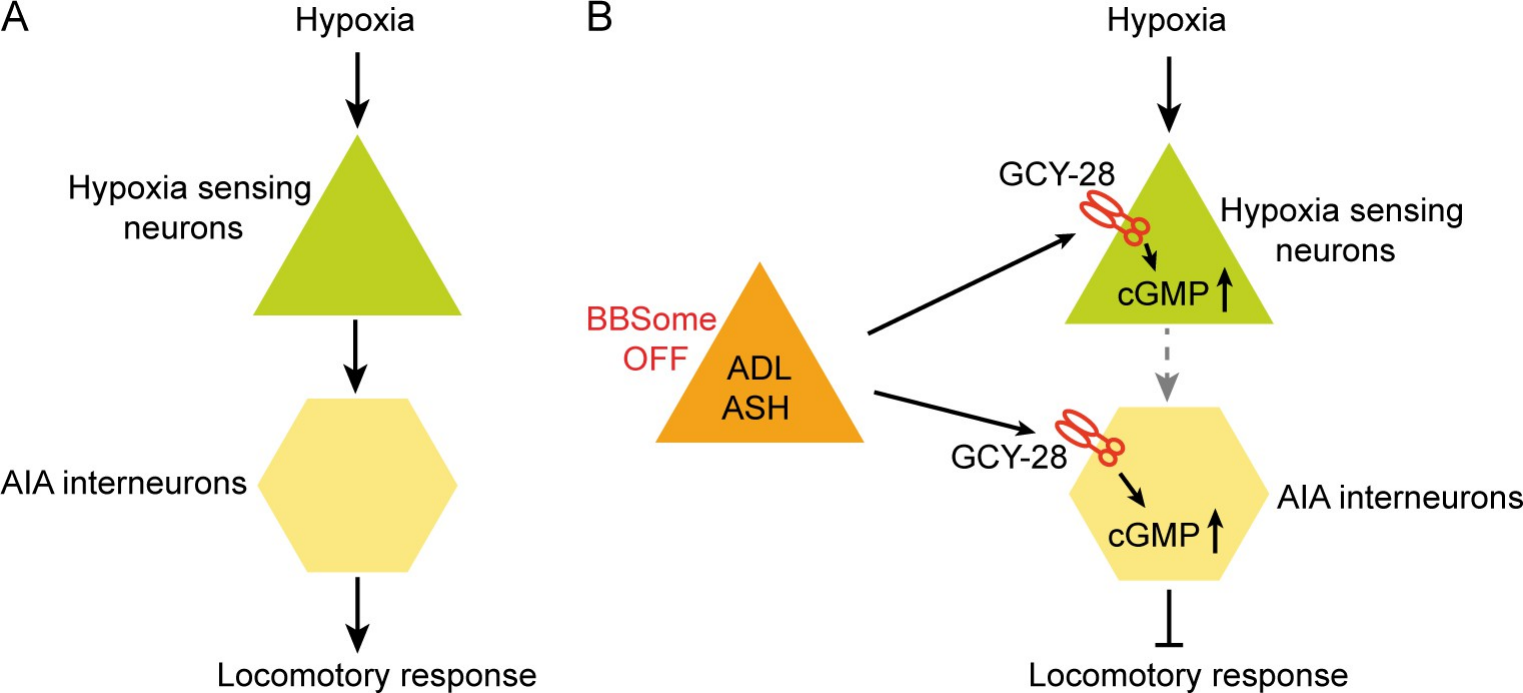
Schematic representation of the main discoveries. **(A)** Multiple sensory neurons are involved in the sensation of acute hypoxia. AIA interneurons are one of the major components in the downstream circuits of acute hypoxia response. **(B)** ADL and ASH neurons are not directly required for acute hypoxia sensing, but they play a modulatory role in locomotory responses to acute hypoxia. When BBSome is disrupted, signals from ADL and ASH neurons overactivates the receptor guanylate cyclase GCY-28 to generate excessive amount of cGMP in the hypoxia sensing circuit and inhibits acute hypoxia responses.

Our work begins to shed light on acute hypoxia responses in *C*. *elegans*. A rapid drop in O_2_ is detected by multiple sensory neurons, which transmit the signals to the downstream circuits including the AIA interneurons to generate escape responses (Fig 9A). The hypoxia sensing circuit can be potently dampened by increased cGMP production (Fig 9B). Even though ADL and ASH neurons are not directly involved in sensing hypoxia, disruption of the BBSome generates or enhances an unknown signal from these 2 neurons to stimulate GCY-28–mediated cGMP production in the hypoxia sensing circuit, thereby inhibiting acute responses to hypoxia (Fig 9B). Many questions still remain to be addressed. We implicate mitochondria and ROS in the sensing mechanism, but have not demonstrated that ROS increases robustly and persistently upon hypoxia exposure, to explain the persistent behavioral arousal evoked in *C*. *elegans* by hypoxia. While we show that hypoxia elicits a decrease in neuronal Ca^2+^ in several neurons, we have not identified the ion channels involved. Nevertheless, the robust behavioral response we describe now allows us to bring to bear the powerful genetics available in *C*. *elegans* to delineate the molecular details of acute hypoxia sensing in this animal.

## Materials and methods

### Strains

*C*. *elegans* were maintained using standard protocols [70]. Strains used are listed in S2 Table.

### Behavioral analysis

*C*. *elegans* locomotion assays were performed as described previously [16]. Briefly, assay plates were seeded with OP50 and grown overnight. To prepare the bacterial lawn for our assays we used a PDMS stamp that had an approximately 0.8 cm × 0.8 cm square cut away from its center. PDMS stamp was made by mixing silicone elastomer base with the curing agent at the ratio of 10:1 (Sylgard 184 silicone elastomer kit) and cured for >1 day in a 15-cm petri dish. Pressing the flat side of this stamp against the bacterial lawn removed the border, leaving an approximately 0.8 cm × 0.8 cm bacterial patch that perfectly fitted our microfluidic chambers. The chamber was placed on top of 25 to 30 young adults and defined gases mixtures delivered into the chamber at a flow rate of 3 ml/min using a PHD 2000 Infusion syringe pump (Harvard Apparatus, United States). Telfon valves (AutoMate Scientific, United States) were used to rapidly switch between 2 gas mixtures (7% and 0%, 7% and 1%, or 7% and 21% O_2_) from the syringe pump. The closing and opening of valve channels were operated by ValveBank Perfusion Controller (AutoMate Scientific). The O_2_ concentration in the chamber was measured using the Fibox sensor system (PreSens, Germany). We placed an oxygen sensor spot (PreSens) inside the chamber and monitored the O_2_ levels as we pumped in gases. To track animals’ locomotory responses to O_2_ stimulation, a Grasshopper camera (FLIR, United States) was mounted on a Zeiss Stemi 508 microscope. Videos were acquired at 2 frames/second for 2 minutes at each O_2_ concentration and analyzed using Zentracker, a custom-written MATLAB program (https://github.com/wormtracker/zentracker). Each data point represents at least 3 assays for each genotype.

To examine the effects of paraquat, rotenone and juglone on acute responses to hypoxia, day 1 adult animals were exposed to 200 mM paraquat for 10 minutes, 10 μM rotenone for 1 hour, and 1 mM juglone for 2 minutes, on drug-containing plates in the presence of food; treated animals were then assayed on regular assay plates.

Optogenetic experiments followed published protocols [16,43]. Transgenic L4 animals were picked to plates either with or without 100 μM ATR (R2500, Sigma, United States), and kept in the dark for 16 to 20 hours. Blue light illumination was from an ultra-high-power LED lamp (UHP-MIC-LED-460, Prizmatix, Israel), attenuated to 70 μW/mm^2^. Light intensity was measured by a PM50 Optical Power Meter (ThorLabs, United States). The transmitted light used for worm picking and video recording was filtered through a long-pass optical filter that blocked light with wavelengths shorter than 595 nm. Videos were captured and analyzed as for regular behavioral assays.

Neuronal inhibition using the histamine gated chloride channel HisCl1 was performed as described [48]. All HisCl1 constructs were injected at 10 ng/μl. To inhibit AIA interneurons, animals at L4 stage were picked to fresh plates containing 10 mM histamine (Sigma, H7250) and grown for 10 minutes. To inhibit the other neurons, animals were grown on histamine containing plates for both 10 minutes and 24 hours. Assays were performed using day 1 adults, on assay plates containing 10 mM histamine. Videos were acquired and analyzed as for regular behavioral assays.

### Molecular biology

The Multisite Gateway system (Thermo Fisher Scientific, United States) was used to make expression vectors. Promoters, including *osm-6* (2.7kb), *gpa-11* (3kb), *srh-220* (2.1kb), *sra-6* (3kb), *gpa-3* (6kb), *gcy-28*.*c* (3.2kb), *gcy-28*.*d* (2.9kb), *gas-1* (1.3kb), *rab-3* (1.2kb), *ges-1* (3.5kb), *myo-3* (2.5kb), *dpy-7* (0.4kb), *odr-3* (2.7kb) *ocr-2* (2.4kb), *odr-1* (2.4kb), *ops-1* (2kb), *flp-21* (4.1kb), *glr-1* (5.3kb), *nmr-1* (2.2kb), *unc-25* (1.8kb), *odr-10* (1.1kb), and *flp-6* (2.7kb), were amplified from N2 genomic DNA and cloned into pDONR P4P1 using BP clonase. The cDNAs for *bbs-9*, *gpa-3*.*a*, *odr-3*, *gcy-28*.*c*, *gcy-28*.*d*, and *gas-1* were amplified by PCR from a first-strand cDNA library made from N2 RNA using M-MuLV reverse transcriptase (Thermo Fisher Scientific), and cloned into pDONR 221 using BP cloning. BeCyclOp and FlincG3 were amplified from plasmids kindly provided by Alexander Gottschalk and Noelle L’Etoile [43,46] and cloned into pDONR 221. Expression vectors were assembled using the LR reaction. The following primers were used to amplify the coding sequences of these genes:

*bbs-9* forward: ggggACAAGTTTGTACAAAAAAGCAGGCTtttcagaaaaATGTCGCTTTTTCGGCTCGTCGAATG

*bbs-9* reverse: ggggACCACTTTGTACAAGAAAGCTGGGTATTAAGCTGTCACTTGTTGCTCTTCCTC

*gpa-3*.*a* forward: ggggACAAGTTTGTACAAAAAAGCAGGCTtttcagaaaaatgggattatgccaatctgcagagg

*gpa-3*.*a* reverse: ggggACCACTTTGTACAAGAAAGCTGGGTAttagaggcctgaaagcaaaagaacgg

*odr-3* forward: ggggACAAGTTTGTACAAAAAAGCAGGCTtttcagaaaaatgggctcatgccagagcaatgaaaattc

*odr-3* reverse: ggggACCACTTTGTACAAGAAAGCTGGGTAttacatcattcctgctttttgtaaattcttctg

*gcy-28*.*c* forward: ggggACAAGTTTGTACAAAAAAGCAGGCTtttcagaaaa ATGCTCAGATGGCTAACTCTACTTTCATG

*gcy-28*.*c* reverse: ggggACCACTTTGTACAAGAAAGCTGGGTACTATGCAAATTCTTCGCCAAAATCGGG

*gcy-28*.*d* forward: ggggACAAGTTTGTACAAAAAAGCAGGCTtttcagaaaa ATGTGGATTAACAGCACGCGAACACTT

*gcy-28*.*d* reverse: ggggACCACTTTGTACAAGAAAGCTGGGTACTATGCAAATTCTTCGCCAAAATCGGG

*gas-1* forward: ggggACAAGTTTGTACAAAAAAGCAGGCTtttcagaaaaATGTTGGGTAGAAAGATCGCCGG

*gas-1* reverse: ggggACCACTTTGTACAAGAAAGCTGGGTATTATCGATCGACCTCTCCGAACAC

BeCyclOp forward: ggggACAAGTTTGTACAAAAAAGCAGGCTtttcagaaaa ATGAAGGACAAGGACAACAACCTCC

BeCyclOp reverse: ggggACCACTTTGTACAAGAAAGCTGGGTATTACTTACGTCCGAGGACCCAGTAGG

Animals transgenic for *bbs-9* and *gpa-3* expression constructs were made by injecting 5 ng/μl of the expression construct, together with 50 ng/μl co-injection marker *unc-122p*::*GFP* and 50 ng/μl DNA ladder (Invitrogen, United States) as carrier. The BeCyclOp and HisCl1 constructs were injected at 10 ng/μl and FlincG3 constructs at 30 ng/μl. To silence the expression of *isp-1* by RNAi, the sense fragment was amplified with the primers (Forward: ggggACAAGTTTGTACAAAAAAGCAGGCTGCTATGGCAGCTGATCAACGTG. Reverse: ggggACCACTTTGTACAAGAAAGCTGGGTACAATTGGGACACATCCAAGATGGG), and the antisense fragment with the primers (Forward: ggggACAAGTTTGTACAAAAAAGCAGGCTCAATTGGGACACATCCAAGATGGG. Reverse: ggggACCACTTTGTACAAGAAAGCTGGGTAGCTATGGCAGCTGATCAACGTG). For *bbs-9* RNAi, the sense fragment was amplified with the primers (Forward: ggggACAAGTTTGTACAAAAAAGCAGGCTCAAGTCCTGATGAAACCCTCGCATC). Reverse: ggggACCACTTTGTACAAGAAAGCTGGGTAAGAGTTTGTGTAGTCTGACAGTAACCG), and the antisense fragment was amplified with the primers (Forward: ggggACAAGTTTGTACAAAAAAGCAGGCTAGAGTTTGTGTAGTCTGACAGTAACCG). Reverse: ggggACCACTTTGTACAAGAAAGCTGGGTACAAGTCCTGATGAAACCCTCGCATC). Sense and antisense fragments were cloned into pDONR 221 using BP cloning and assembled with different promoters into expression vectors using the LR reaction. *isp-1* sense and antisense constructs were co-injected at a concentration of 100ng/μl for the promoters *col-12*, *myo-3*, *gpa-3*, *gpa-11*, *gcy-28*.*c*, and *gcy-28*.*d*, 30 ng/μl for the promoters *dpy-7*, *vha-6*, and *rab-3*, and 10 ng/μl for the promoters *isp-1* and *myo-2*. *bbs-9* RNAi constructs were injected at a concentration of 100 ng/μl. All other expression constructs were injected at 50 ng/μl.

### CRISPR/Cas-9–based genome editing

Genome editing with CRISPR/Cas-9 was performed as described [71]. To generate gene knockouts, we used a single-strand DNA oligo (ssODN) repair template that targeted sequences early in the coding region of the gene. The ssODN contained 2 homology arms, each of 35 bases that flanked either side of the targeted PAM site while deleting 16 bases of coding sequence to generate a frameshift mutation. In addition, we introduced 2 stop codons and a unique restriction site for genotyping between the 2 flanking sequences. To generate point mutations, we chose a PAM site close to the codon of interest and used a ssODN donor that included 35 bases homologous to sequences on either side of the targeted codon. If the PAM site was not close to the codon, the homology arms were extended accordingly. GFP knockins were generated by using double strand DNA repair templates [72]. GFP specific oligos tailed with 35bp homology arms were used to amplify *gfp* coding sequence. The template was melted as described to improve editing efficiency. Moreover, 0.5 μl of Cas-9 protein (IDT) was mixed with 5 μl of 0.4μg/μl tracrRNA (IDT, United States) and 2.8 μl of 0.4 μg/μl crRNA (IDT). The mix was incubated at 37°C for at least 10 minutes before adding 2.2 μl of 1μg/μl ssODN (or 500 ng dsDNA) and 2 μl of 0.6 μg/μl *rol-6* co-injection marker. Nuclease-free water was used to bring the final volume to 20 μl. The injection mixture was centrifuged for at least 2 minutes before use. Large deletions were generated with an injection mix that included 2 crRNA guides targeting the start and the end of the gene. Moreover, 0.5 μl of Cas-9 protein, 5 μl of 0.4μg/μl tracrRNA, and 1.4 μl of 0.4 μg/μl each of 2 crRNA guides were mixed and incubated at 37°C. Furthermore, 2 μl of 0.6 μg/μl *rol-6* co-injection marker and nuclease-free water was added after incubation to bring the final volume to 20 μl.

### Microscopy

FlincG3 was used to report neuronal cGMP levels; in these experiments, we performed pseudoratiometric imaging by collecting both GFP (from FlincG3) and free mCherry signals. Transgenic animals at L4 stage were picked and grown overnight under standard laboratory conditions. Day 1 adult animals were mounted on 2% agarose pads containing 5 mM levamisole. Z-stack confocal Images of 20 to 50 animals were collected using a 63× oil objective N.A 1.3 on a Nikon A1 confocal microscope with Nikon NIS elements software. A similar procedure was followed to quantify the neuropeptide secretion. Only the signals from posterior pair of coelomocytes were collected. The mCherry signal of neuropeptides and GFP signals from *Punc-122*::*gfp* were captured simultaneously for pseudoratiometric comparison. Signals from 20 to 50 animals were collected. For GPCR, G protein and *gcy-28* expression, the absolute GFP intensity was calculated and compared among different genotypes. All images were processed in Fiji by subtracting background, adjusting threshold, and analyzing the particles in the region of interest.

### Dye filling

Synchronized 1-day adults were soaked for 1 hour in 1 ml of DiO or DiI (Molecular Probes, United States), diluted 1:200 in M9 from a 2 mg/ml stock solution in DMF. The worms were washed 3× with 1 ml M9 and destained on seeded plates for > 2 hours. DiO and DiI uptake was monitored by mounting live animals for microscopy and imaging as described above.

### DHE staining

Synchronized 1-day adults were washed 3× in PBS and stained in 3 μM of DHE (diluted from a 30 mM DHE stock solution in DMSO) for 30 minutes. Worms were washed twice with 1ml PBS and immobilized on a 2% agarose pad for confocal imaging. Images were acquired and processed as described above.

### cGMP quantification in worm lysates

Total cGMP in *C*. *elegans* lysates was measured using a direct cyclic GMP enzyme immunoassay kit (Arbor Assays, United States). Moreover, 3 to 4 plates of synchronized young adult animals were harvested and washed 3× with PBS. The worm pellet was washed again with 1 ml 0.1 M HCl. A total of 50 μl of worm pellet was resuspended in 400 μl 0.1M HCl and frozen in liquid nitrogen. For worms treated with paraquat, animals were exposed in the paraquat solution after PBS washing. Paraquat was immediately replaced with PBS, and the worms washed quickly 3× with PBS before resuspension in 0.1 M HCl. Worms were disrupted using a bead-beater with 0.5-mm glass beads. The worm lysate was centrifugated at 2,500 rpm for 2 minutes. Protein concentration was determined using the Quick start Bradford dye reagent (Bio-Rad, United States). cGMP in 20 μl of lysate was quantified by following the acetylation protocol from Arbor Assays. The optical density at 450 nm was read using a ClarioStar 96-well microplate reader (BMG Labtech, Germany). The cGMP concentration was calculated using a curve generated from cGMP standards in the kit and normalized to sample protein concentration.

### Ca^2+^ imaging

Ca^2+^ imaging of immobilized animals was performed using YC2.60 (RMG neurons) and YC3.60 (ASK, AFD, and AIA neurons) on an inverted microscope (Axiovert, Zeiss, Germany), with a 40× 1.2 NA C-Apochromat lens, and MetaMorph acquisition software (Molecular Devices, United States). Worms were glued to agarose pads (2% in M9 buffer, 1 mM CaCl_2_) using Dermabond tissue adhesive with their nose and tail immersed in a mix of OP50 and M9 buffer. The glued animals were sealed in an airtight microfluidic chamber, and the O_2_ was delivered and measured as described in the behavioral analysis. Recordings were carried out at 2 frames/sec with an exposure time of 100 ms using MetaMorph software. Photobleaching was minimized using 2.0 or 1.5 optical density filters. An excitation filter (Chroma, United States) restricted illumination to the cyan channel, and a beam splitter (Optical Insights, UK) was used to separate the cyan and yellow emission light. Neuron Analyzer, a custom-written MATLAB program, was used to analyze the resulting image stacks (https://github.com/neuronanalyser/neuronanalyser) [16].

### Neuron ablation

Cell ablations were performed as described [73]. Briefly, L4 transgenic worms expressing membrane-anchored miniSOG in different neurons were picked to 3-cm plates without bacteria, exposed to 2 mW/mm^2^ blue light from an ultra-high-power LED lamp (UHP-MIC-LED-460, Prizmatix) for 5 minutes, transferred back to the seeded plates and kept in the dark for 12 hours. Videos were acquired and analyzed as regular behavioral assays.

## Supporting information

S1 Fig Acute locomotory responses of *C*. *elegans* to hypoxia. **(A)** Reorientation movements (omega turns) of WT animals to 7% to 1% O_2_ stimuli. **(B)** Changes in reversal frequency in WT animals experiencing a 7% to 1% O_2_ switch. **(C)** Changes in the locomotory activity of WT animals evoked by a switch from 7% to 1% O_2_. **(D)** Locomotory activity of WT was recorded for 2 minutes at 7% O_2_ and 20 minutes at 1% O_2_ followed by 2 additional minutes at 7% O_2_. **(E)** Locomotory responses of WT animals exposed to repeated switches between 7% and 1% O_2_. **(F)** Locomotory responses of WT animals to switches from 7% to approximately 0.2% O_2_ and to switches from 7% to approximately 1% O_2_. **(G)** Hypoxia-evoked locomotory responses of WT (N2) and *npr-1* mutant animals. NS = not significant, Mann–Whitney U test. The source code underlying behavioral data can be found at https://github.com/wormtracker/zentracker. O_2_, oxygen; WT, wild-type.
(TIF)

S2 Fig BBSome mutants are defective in acute responses to 1% but not 21% O_2_. **(A and B)** Locomotory responses to 7% to 1% O_2_ stimuli of WT, *bbs-1(ok1111)*, *bbs-2(ok2053)*, *bbs-4(yum64)*, *bbs-7(ok1351)*, and *bbs-8(nx77)* animals. **** = *p* < 0.0001. ANOVA, Tukey multiple comparison. **(C and D)** Locomotory responses to indicated changes in O_2_ concentration of *npr-1(ad609)* and *npr-1(ad609); dyf-3(m185)* animals (C), and *npr-1(ad609)* and *npr-1(ad609); bbs-9(gk471)* animals (D). NS = not significant (21% O_2_), **** = *p* < 0.0001 (1% O_2_), Mann–Whitney U test. **(E)** Locomotory responses to 7% to 1% O_2_ stimuli of animals of indicated genotypes: WT, and WT expressing *bbs-9* RNAi constructs in ADL and ASH neurons from the *gpa-11* promoter. **** = *p* < 0.0001. Mann–Whitney U test. The source code underlying behavioral data can be found at https://github.com/wormtracker/zentracker. RNAi, RNA interference; O_2_, oxygen; WT, wild-type.
(TIF)

S3 Fig Mutations in *gcy-28* or *egl-4* do not suppress the dye-filling defects of *dyf-3* or *bbs-9* mutants. **(A and B)** DiO dye-filling of WT, *dyf-3(m185)*, *egl-4(n478)*, *gcy-28(yum32)*, *bbs-9(gk471)* animals, and indicated double mutant combinations of these alleles. WT, wild-type.
(TIF)

S4 Fig cGMP signaling modulates acute hypoxia responses. **(A)** Locomotory responses of *pde* single mutants to switches between 7% and 1% O_2_. NS = not significant, * = *p* < 0.05. ANOVA, Tukey multiple comparison. **(B–E)** Locomotory responses of *pde* double mutants to 1% O_2_ stimulation: *pde-1(tm3765); pde-2(tm3098)* and *pde-1(tm3765); pde-3(Q104stop)* (B); *pde-1(tm3765); pde-5(ok3102)* and *pde-2(tm3098); pde-3(Q104stop)* (C); *pde-2(tm3098); pde-5(ok3102)* and *pde-3(Q104stop); pde-5(ok3102)* (D); *pde-4(ok1290); pde-6(ok3410)* (E). NS = not significant, * = *p* < 0.05, ** = *p < 0*.*01*, **** = *p* < 0.0001. ANOVA, Tukey multiple comparison (B–D), and Mann–Whitney U test (E). **(F–H)** Locomotory responses to switches between 7% and 1% O_2_ of indicated genotypes: WT, *bbs-9(gk471)*, *tax-4(p678)*, and *bbs-9(gk471); tax-4(p678)* double mutants (F); WT, *bbs-9(yum38)*, *cng-1(jh113); cng-3(jh111)* double, and *bbs-9(yum38); cng-1(jh113); cng-3(jh111)* triple mutants (G); WT, *bbs-9(gk471)*, *cng-4(e1126)*, and *bbs-9(gk471); cng-4(e1126)* double mutants (H). ** = *p < 0*.*01*, NS = not significant. ANOVA, Tukey multiple comparison. The source code underlying behavioral data can be found at https://github.com/wormtracker/zentracker. O_2_, oxygen; WT, wild-type.
(TIF)

S5 Fig *bbs* mutants have enhanced cGMP signaling. **(A)** Locomotory responses to 1% O_2_ of the mutants defective in all soluble guanylate cyclase genes in either a WT or *bbs-9(gk471)* background. * = *p* < 0.05, ** = *p < 0*.*01*. ANOVA, Tukey multiple comparison. **(B)** Locomotory responses to switches between 7% and 1% O_2_ in *bbs-9(gk471)* mutants lacking various receptor guanylate cyclases. **(C and D)** Locomotory responses to switches between 7% and 1% O_2_ of WT animals overexpressing *gcy-35* (C) or *gcy-15* (D) guanylate cyclases. NS = not significant, Mann–Whitney U test. **(E)** Representative images of the genetically encoded cGMP sensor FlincG3 expressed together with a mCherry marker in AFD neurons using the *gcy-8* promoter and imaged in WT and *bbs-9(gk471)* mutants. Arrows point to the neuronal cell bodies. **(F)** Quantification of FlincG3 fluorescent signal intensity in AFD neurons normalized to mCherry fluorescence intensity. **** = *p* < 0.0001, *t* test. **(G)** Representative images of the genetically encoded cGMP sensor FlincG3 expressed together with a mCherry marker in AIA neurons using the *gcy-28*.*d* promoter and imaged in WT and *bbs-9(gk471)* mutants. Arrows point to the neuronal cell bodies. **(H)** Quantification of FlincG3 fluorescent signal intensity in AIA neurons normalized to mCherry fluorescence intensity. ** = *p* < 0.01, *t* test. The underlying data can be found in S1 Data, and the source code can be found at https://github.com/wormtracker/zentracker. O_2_, oxygen; WT, wild-type.
(TIF)

S6 Fig cGMP production by GCY-28 in multiple neurons contributes to inhibition of acute responses to hypoxia. **(A)** Fluorescence expression of a *gcy-28*.*c*::*gfp* construct driven from its endogenous promoter (top panel). The dashed circles indicate the expected locations of ADL and ASH neurons highlighted by dye-filling with DiI (middle panel), seen overlaid with *gcy-28*.*c*::*gfp* expression (bottom panel). **(B)** Locomotory responses to hypoxia of transgenic animals expressing *gcy-28*.*c* cDNA in neurons (*Prab-3*), intestine (*Pges-1*), muscle (*Pmyo-3*), and hypodermis (*Pdpy-7*). NS = not significant, **** = *p* < 0.0001. ANOVA, Tukey multiple comparison. **(C–I)** Locomotory responses to hypoxia of transgenic animals overexpressing *gcy-28*.*c* in different subsets of neurons. The detailed expression pattern of each promoter is listed in the table. NS = not significant, * = *p* < 0.05, **** = *p* < 0.0001. Mann–Whitney U test (C), and ANOVA, Tukey multiple comparison (D–I). The source code underlying behavioral data can be found at https://github.com/wormtracker/zentracker.
(TIF)

S7 Fig Modulation of acute hypoxia responses by optogenetic manipulation. **(A and B)** Locomotory responses to hypoxia of WT animals expressing BeCyclOp from the *gcy-28*.*c* promoter (A), or the *gpa-3* promoter (B), without ATR treatment but with blue light exposure. NS = not significant, Mann–Whitney U test. **(C)** WT animals expressing BeCyclOp from the *gpa-11* promoter in the presence of ATR do not show light-dependent inhibition of hypoxia–evoked locomotory responses. NS = not significant. ANOVA, Tukey multiple comparison. The source code underlying behavioral data can be found at https://github.com/wormtracker/zentracker. WT, wild-type.
(TIF)

S8 Fig GCY-28 expression and BBSome function. **(A)** Representative images (left panels) and quantification (right panel) of GFP fluorescence from expression of a single copy GCY-28.C-GFP inserted using MosSCI. ** = *p* < 0.01, *t* test. **(B)** Representative images (left panels) and quantification (right panel) of GFP fluorescence from expression of an extrachromosomal array of GCY-28.D-GFP in AIA neurons. ** = *p* < 0.01, *t* test. **(C)** Locomotory responses to 7% to 1% O_2_ stimuli of animals treated with 10 mM histamine: WT, and WT expressing HisCl1 from *sra-6* (ASH), *srh-220* (ADL) and *gpa-11* (ASH and ADL) promoters. NS = not significant. ANOVA, Tukey multiple comparison. **(D)** Locomotory responses to 7% to 1% O_2_ stimuli of animals treated with 2 mW/mm^2^ blue light for 5 minutes: WT, and WT expressing PH-miniSOG from the *sra-6* (ASH), *srh-220* (ADL) and *gpa-11* (ASH and ADL) promoters. NS = not significant. ANOVA, Tukey multiple comparison. **(E)** Representative images (left panels) and quantification (right panel) of ODR-3::GFP in AWA cilia. Arrowheads point to AWA cilia. The GFP intensity in WT was arbitrarily set to 1, and the GFP signal in *bbs-9(gk471)* mutants was normalized to WT. ** = *p* < 0.01, *t* test. The underlying data can be found in S1 Data, and the source code can be found at https://github.com/wormtracker/zentracker. O_2_, oxygen; WT, wild-type.
(TIF)

S9 Fig Perturbation of mitochondrial function attenuates acute responses to hypoxia. **(A)** Locomotory responses to 7% to 1% O_2_ stimuli of animals of indicated genotypes: WT, *isp-1*(*qm150*), and *isp-1*(*qm150*) expressing 2 *isp-1* containing fosmids. **** = *p* < 0.0001, *** = *p* < 0.001, NS = not significant. ANOVA, Tukey multiple comparison. **(B)** Locomotory responses to 7% to 1% O_2_ stimuli of animals of indicated genotypes: WT, *isp-1*(*qm150*), and CRISPR repaired *isp-1*(*qm150*), in which serine at position 225 was replaced by proline as in WT. **** = *p* < 0.0001. ANOVA, Tukey multiple comparison. **(C and D)** Locomotory responses to 7% to 1% O_2_ stimuli of animals of indicated genotypes: WT, and WT expressing *isp-1* RNAi constructs under *dpy-7* (hypodermis) and *vha-6* (intestine) promoters (C); WT, and WT expressing *isp-1* RNAi constructs under *col-12* (epithelium) and *myo-3* (muscle) promoters (D). * = *p* < 0.05, NS = not significant. ANOVA, Tukey multiple comparison. **(E)** Locomotory responses to indicated changes in O_2_ concentration of *npr-1(ad609)* and *npr-1(ad609)* expressing *isp-1* RNAi constructs pan-neuronally. NS = not significant (21% O_2_), **** = *p* < 0.0001 (1% O_2_). Mann–Whitney U test. **(F)** Representative images of DHE staining of various genotypes. **(G)** Quantification of DHE staining. ** = *p* < 0.01, **** = *p* < 0.0001, NS = not significant. ANOVA, Tukey multiple comparison. **(H)** Hypoxia-evoked locomotory responses of WT, *sod-2(ok1030)*, and *sod-2(ok1030); sod-5 (tm1146) sod-1(tm783); sod-4(gk101); sod-3(tm760)* mutants. ** = *p < 0*.*01*, **** = *p* < 0.0001. ANOVA, Tukey multiple comparison. **(I)** Animals were exposed to 1 mM juglone for 2 minutes and immediately assayed for their locomotory responses to 21% and 1% O_2_. ** = *p < 0*.*01*, **** = *p* < 0.0001. Mann–Whitney U test. The underlying data can be found in S1 Data, and the source code can be found at https://github.com/wormtracker/zentracker. DHE, dihydroethidium; O_2_, oxygen; RNAi, RNA interference; WT, wild-type.
(TIF)

S10 Fig ROS robustly stimulates cGMP production. **(A)** Total cGMP in worm lysates determined by cGMP enzyme immunoassay of indicated genotypes (*n* = 4): WT, *gas-1(fc21)* and *isp-1*(*qm150*). **** = *p* < 0.0001, and ** = *p* < 0.01. ANOVA, Tukey multiple comparison. **(B)** Staged L4 animals were exposed to 0.4 mM, 4 mM or 50 mM paraquat for different time periods, and their total cGMP levels determined by ELISA (*n* = 3). **(C)** Total cGMP levels in worm lysates of indicated genotypes treated with or without 0.4 mM paraquat and 2 mM NAC. cGMP levels were measured using a cGMP enzyme immunoassay. **** = *p* < 0.0001, NS = not significant. ANOVA, Tukey multiple comparison. **(D)** Locomotory responses to indicated changes in O_2_ concentration of *npr-1(ad609)* and *npr-1(ad609)* expressing *isp-1* RNAi constructs from the *gpa-3* promoter. NS = not significant (21% O_2_), **** = *p* < 0.0001 (1% O_2_), Mann–Whitney U test. The underlying data can be found in S1 Data, and the source code can be found at https://github.com/wormtracker/zentracker. NAC, N-acetylcysteine; RNAi, RNA interference; ROS, reactive oxygen species; O_2_, oxygen; WT, wild-type.
(TIF)

S1 Table The candidate screen for mutants that were defective in acute response to hypoxia. The response of each mutant was compared to that of WT. (−) indicates 0% to 10% of WT responses; (+) indicates 10% to 50% of WT responses; (++) indicates 50% to 80% of WT responses; and (+++) indicates 80% to 100% of WT responses. (*) in the table indicates that the strain CHS10032 has a unique response to acute hypoxia. WT, wild-type.
(PDF)

S2 Table The list of strains used in this study. (DOCX)

S1 Data Underlying data for figures. (XLSX)

## Abbreviations

ANP: atrial natriuretic peptide
ATR: all trans-retinal
BBS: Bardet–Biedel syndrome
CNG: cyclic nucleotide gated
DHE: dihydroethidium
MCI: mitochondrial complex I
MCIII: mitochondrial complex III
NAC: N-acetyl cysteine
O_2_: oxygen
PDE: phosphodiesterase
RNAi: RNA interference
ROS: reactive oxygen species
SOD: superoxide dismutase
ssODN: single-strand DNA oligo
