# Supplementary figures and images for: ROS and cGMP signaling modulate persistent escape from hypoxia in Caenorhabditis elegans
Source: PLoS Biol. 2022 Jun 21;20(6):e3001684. doi: 10.1371/journal.pbio.3001684 (PMC9249223; doi:10.1371/journal.pbio.3001684)

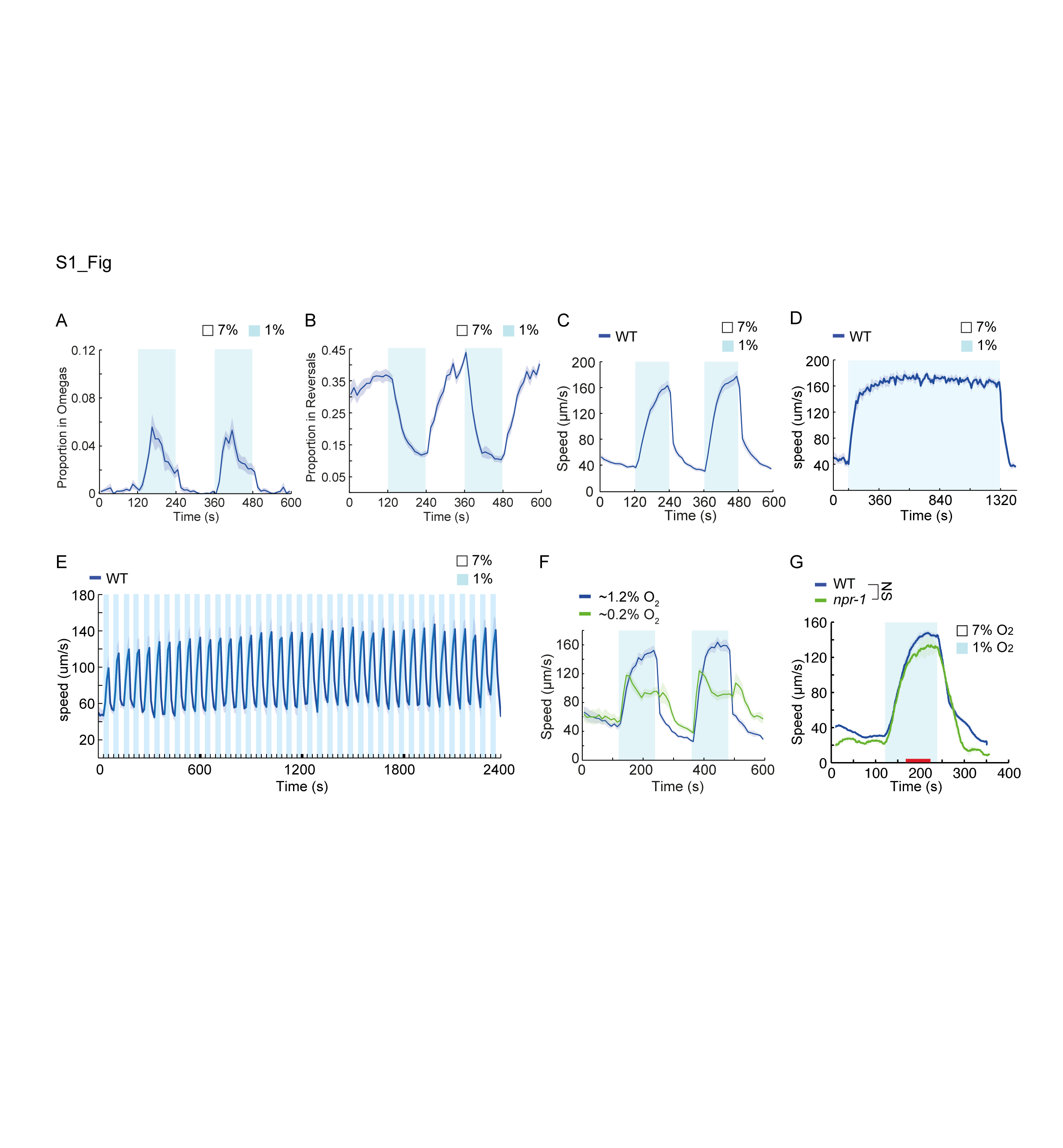

Supplement: S1 Fig — (A) Reorientation movements (omega turns) of WT animals to 7% to 1% O2 stimuli. (B) Changes in reversal frequency in WT animals experiencing a 7% to 1% O2 switch. (C) Changes in the locomotory activity of WT animals evoked by a switch from 7% to 1% O2. (D) Locomotory activity of WT was recorded for 2 minutes at 7% O2 and 20 minutes at 1% O2 followed by 2 additional minutes at 7% O2. (E) Locomotory responses of WT animals exposed to repeated switches between 7% and 1% O2. (F) Locomotory responses of WT animals to switches from 7% to approximately 0.2% O2 and to switches from 7% to approximately 1% O2. (G) Hypoxia-evoked locomotory responses of WT (N2) and npr-1 mutant animals. NS = not significant, Mann–Whitney U test. The source code underlying behavioral data can be found at https://github.com/wormtracker/zentracker. O2, oxygen; WT, wild-type. (TIF) [file pbio.3001684.s001.tif]

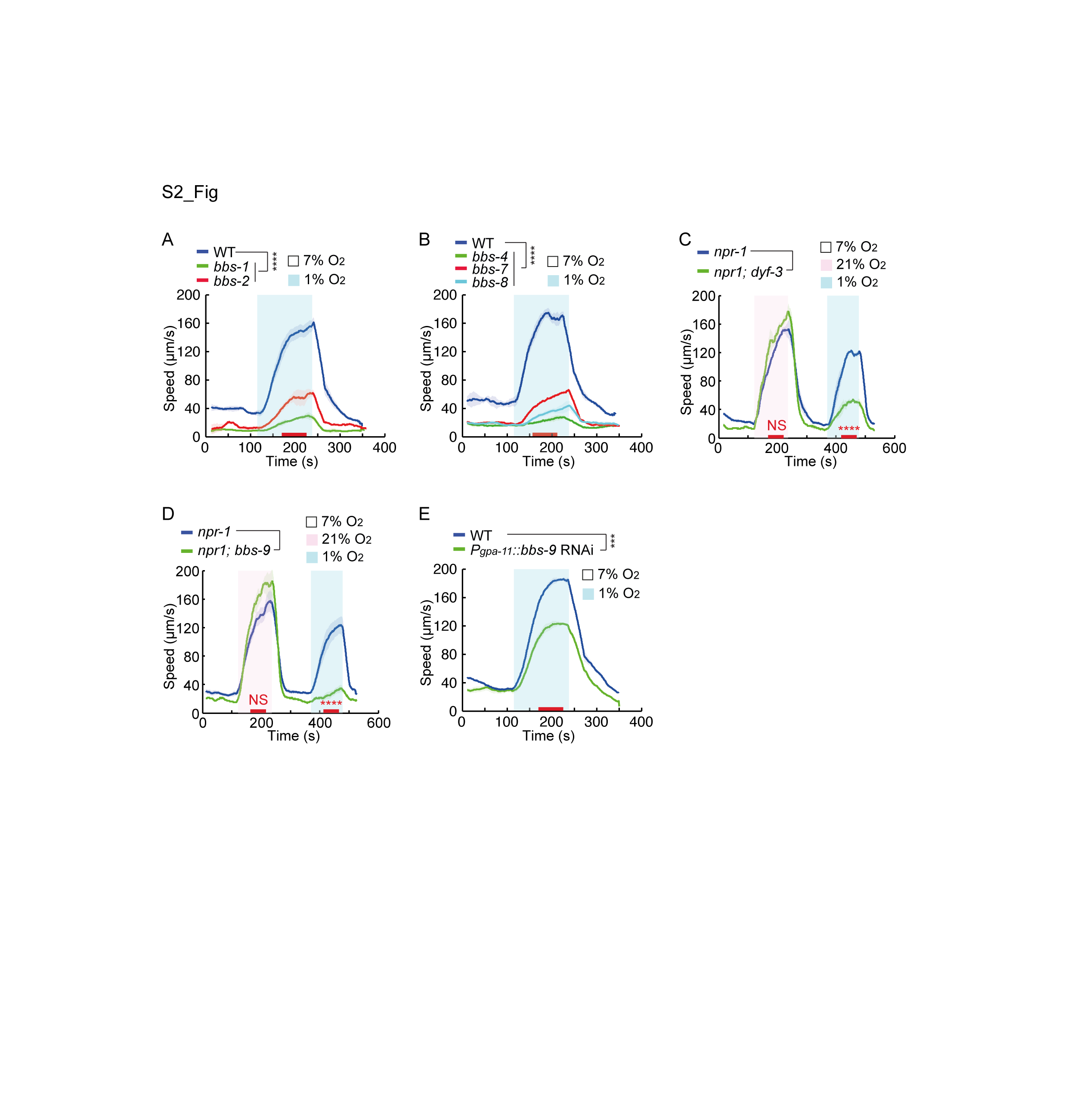

Supplement: S2 Fig — (A and B) Locomotory responses to 7% to 1% O2 stimuli of WT, bbs-1(ok1111), bbs-2(ok2053), bbs-4(yum64), bbs-7(ok1351), and bbs-8(nx77) animals. **** = p < 0.0001. ANOVA, Tukey multiple comparison. (C and D) Locomotory responses to indicated changes in O2 concentration of npr-1(ad609) and npr-1(ad609); dyf-3(m185) animals (C), and npr-1(ad609) and npr-1(ad609); bbs-9(gk471) animals (D). NS = not significant (21% O2), **** = p < 0.0001 (1% O2), Mann–Whitney U test. (E) Locomotory responses to 7% to 1% O2 stimuli of animals of indicated genotypes: WT, and WT expressing bbs-9 RNAi constructs in ADL and ASH neurons from the gpa-11 promoter. **** = p < 0.0001. Mann–Whitney U test. The source code underlying behavioral data can be found at https://github.com/wormtracker/zentracker. RNAi, RNA interference; O2, oxygen; WT, wild-type. (TIF) [file pbio.3001684.s002.tif]

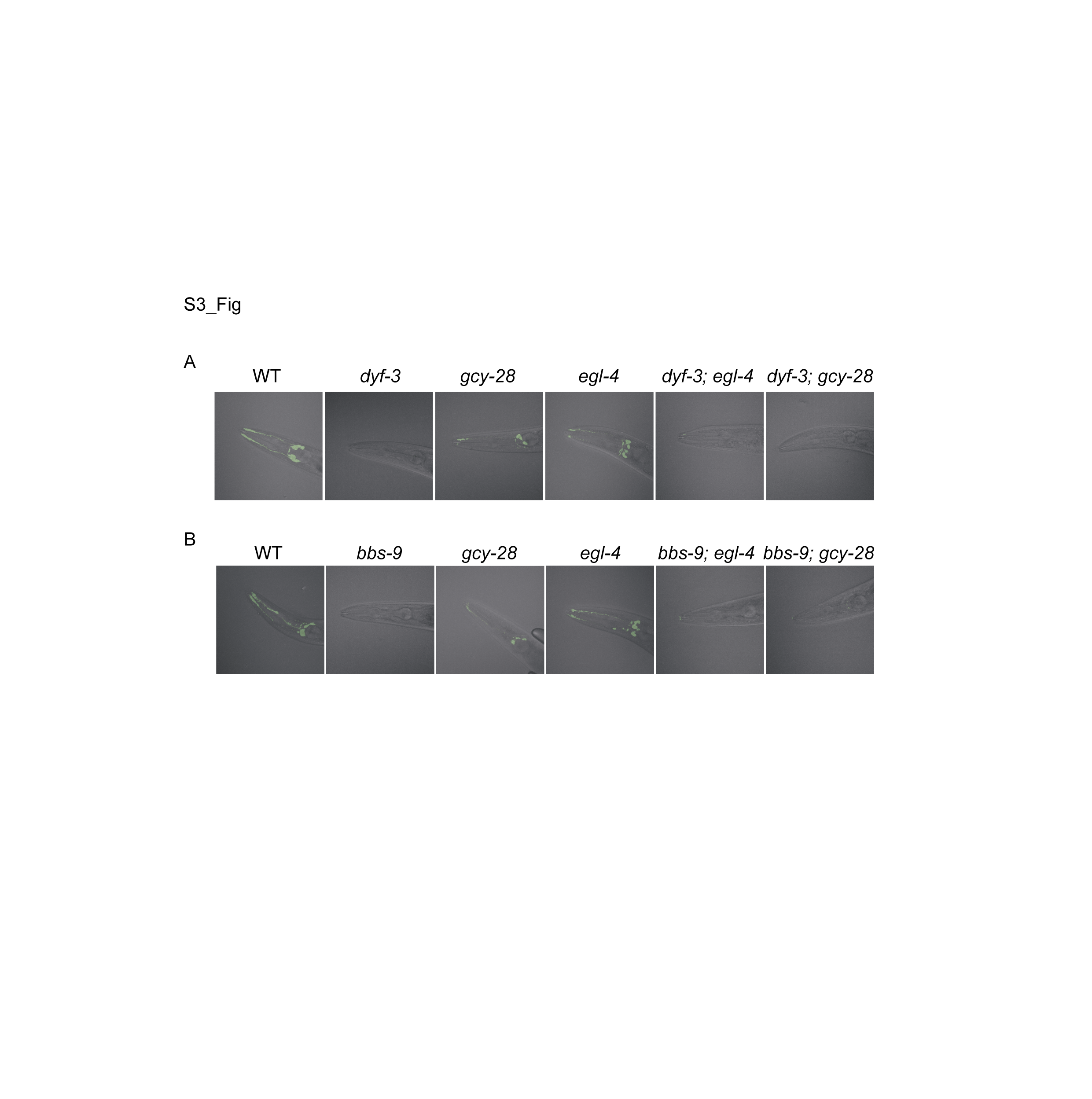

Supplement: S3 Fig — (A and B) DiO dye-filling of WT, dyf-3(m185), egl-4(n478), gcy-28(yum32), bbs-9(gk471) animals, and indicated double mutant combinations of these alleles. WT, wild-type. (TIF) [file pbio.3001684.s003.tif]

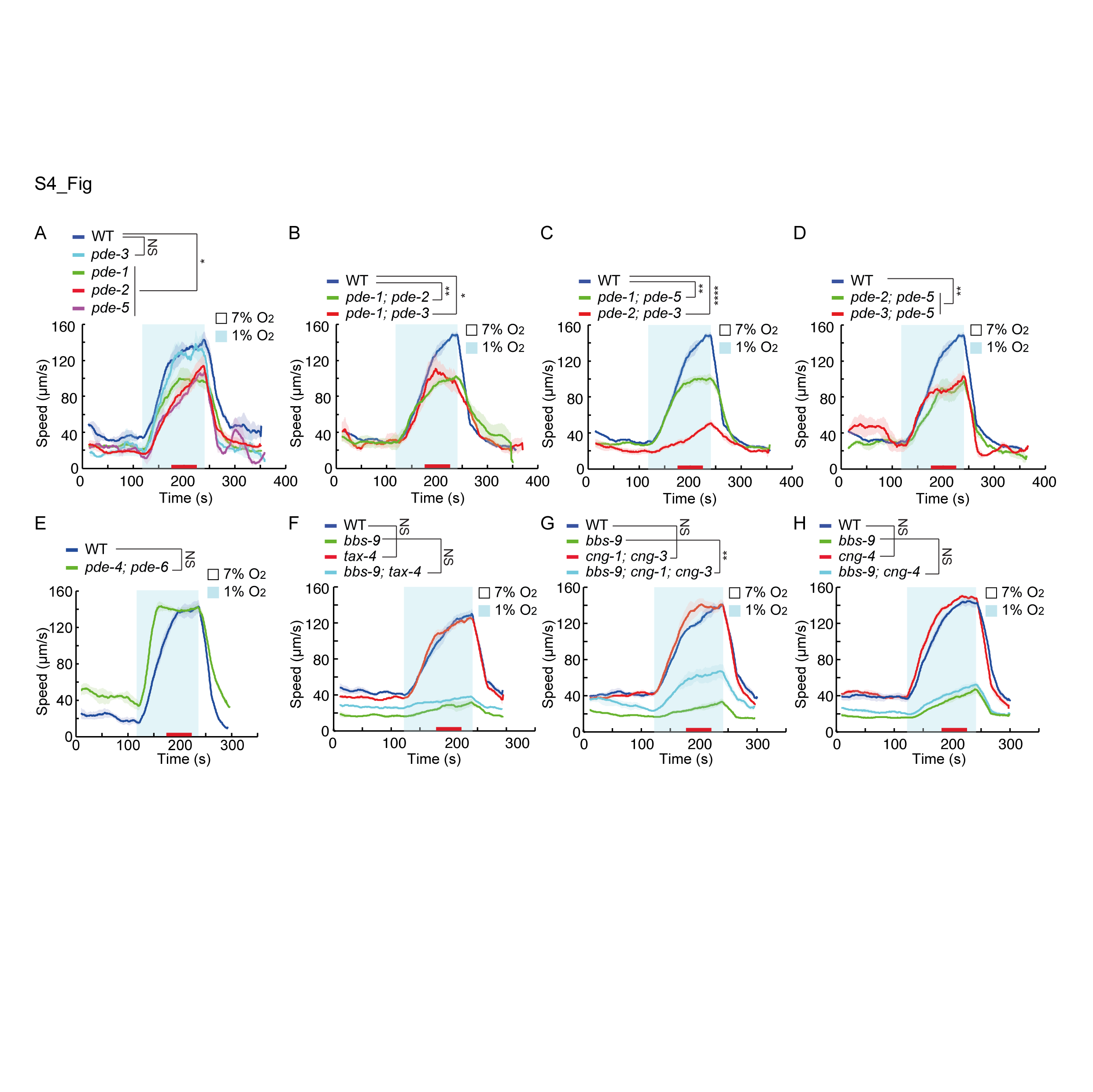

Supplement: S4 Fig — (A) Locomotory responses of pde single mutants to switches between 7% and 1% O2. NS = not significant, * = p < 0.05. ANOVA, Tukey multiple comparison. (B–E) Locomotory responses of pde double mutants to 1% O2 stimulation: pde-1(tm3765); pde-2(tm3098) and pde-1(tm3765); pde-3(Q104stop) (B); pde-1(tm3765); pde-5(ok3102) and pde-2(tm3098); pde-3(Q104stop) (C); pde-2(tm3098); pde-5(ok3102) and pde-3(Q104stop); pde-5(ok3102) (D); pde-4(ok1290); pde-6(ok3410) (E). NS = not significant, * = p < 0.05, ** = p < 0.01, **** = p < 0.0001. ANOVA, Tukey multiple comparison (B–D), and Mann–Whitney U test (E). (F–H) Locomotory responses to switches between 7% and 1% O2 of indicated genotypes: WT, bbs-9(gk471), tax-4(p678), and bbs-9(gk471); tax-4(p678) double mutants (F); WT, bbs-9(yum38), cng-1(jh113); cng-3(jh111) double, and bbs-9(yum38); cng-1(jh113); cng-3(jh111) triple mutants (G); WT, bbs-9(gk471), cng-4(e1126), and bbs-9(gk471); cng-4(e1126) double mutants (H). ** = p < 0.01, NS = not significant. ANOVA, Tukey multiple comparison. The source code underlying behavioral data can be found at https://github.com/wormtracker/zentracker. O2, oxygen; WT, wild-type. (TIF) [file pbio.3001684.s004.tif]

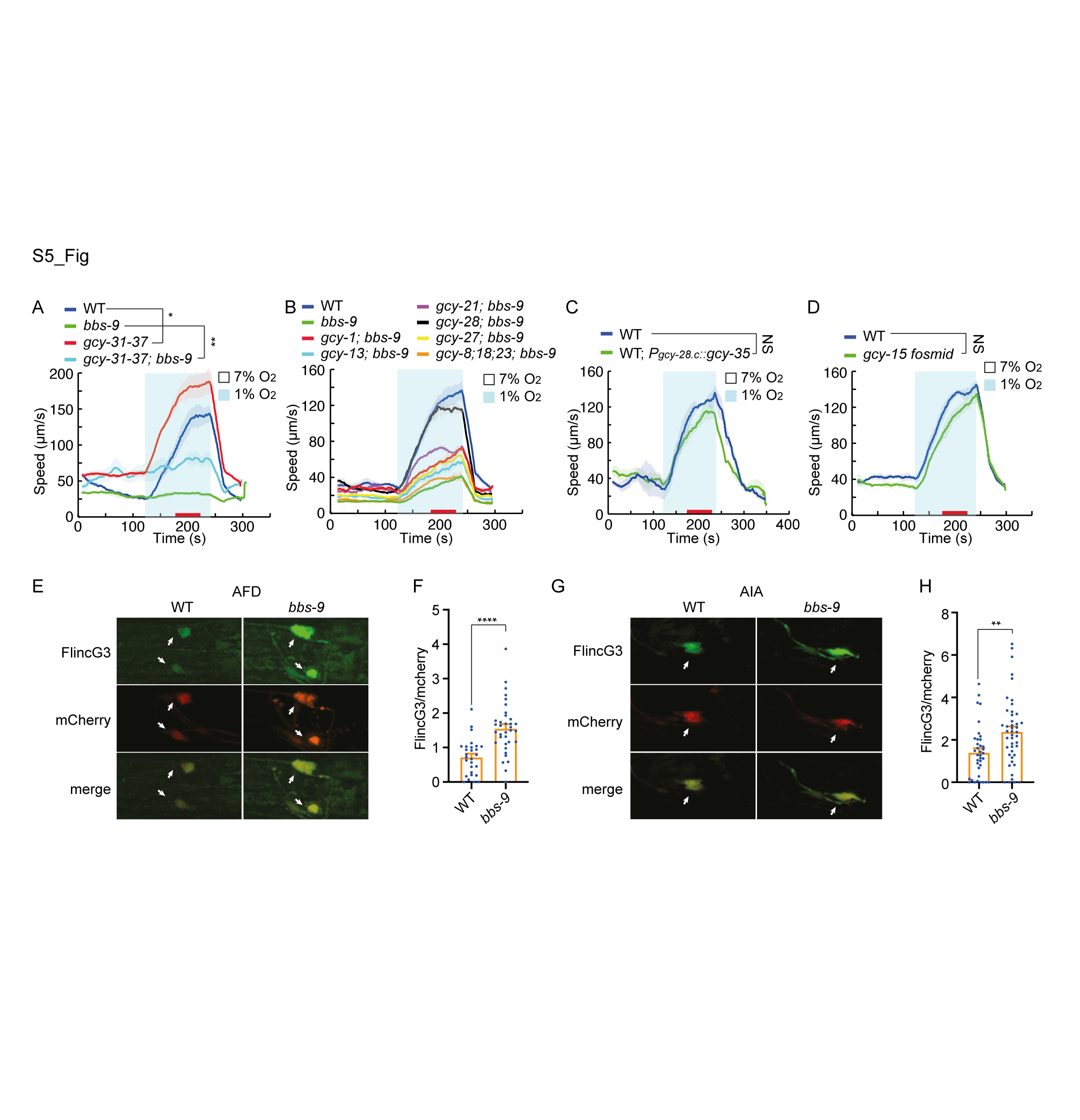

Supplement: S5 Fig — (A) Locomotory responses to 1% O2 of the mutants defective in all soluble guanylate cyclase genes in either a WT or bbs-9(gk471) background. * = p < 0.05, ** = p < 0.01. ANOVA, Tukey multiple comparison. (B) Locomotory responses to switches between 7% and 1% O2 in bbs-9(gk471) mutants lacking various receptor guanylate cyclases. (C and D) Locomotory responses to switches between 7% and 1% O2 of WT animals overexpressing gcy-35 (C) or gcy-15 (D) guanylate cyclases. NS = not significant, Mann–Whitney U test. (E) Representative images of the genetically encoded cGMP sensor FlincG3 expressed together with a mCherry marker in AFD neurons using the gcy-8 promoter and imaged in WT and bbs-9(gk471) mutants. Arrows point to the neuronal cell bodies. (F) Quantification of FlincG3 fluorescent signal intensity in AFD neurons normalized to mCherry fluorescence intensity. **** = p < 0.0001, t test. (G) Representative images of the genetically encoded cGMP sensor FlincG3 expressed together with a mCherry marker in AIA neurons using the gcy-28.d promoter and imaged in WT and bbs-9(gk471) mutants. Arrows point to the neuronal cell bodies. (H) Quantification of FlincG3 fluorescent signal intensity in AIA neurons normalized to mCherry fluorescence intensity. ** = p < 0.01, t test. The underlying data can be found in S1 Data, and the source code can be found at https://github.com/wormtracker/zentracker. O2, oxygen; WT, wild-type. (TIF) [file pbio.3001684.s005.tif]

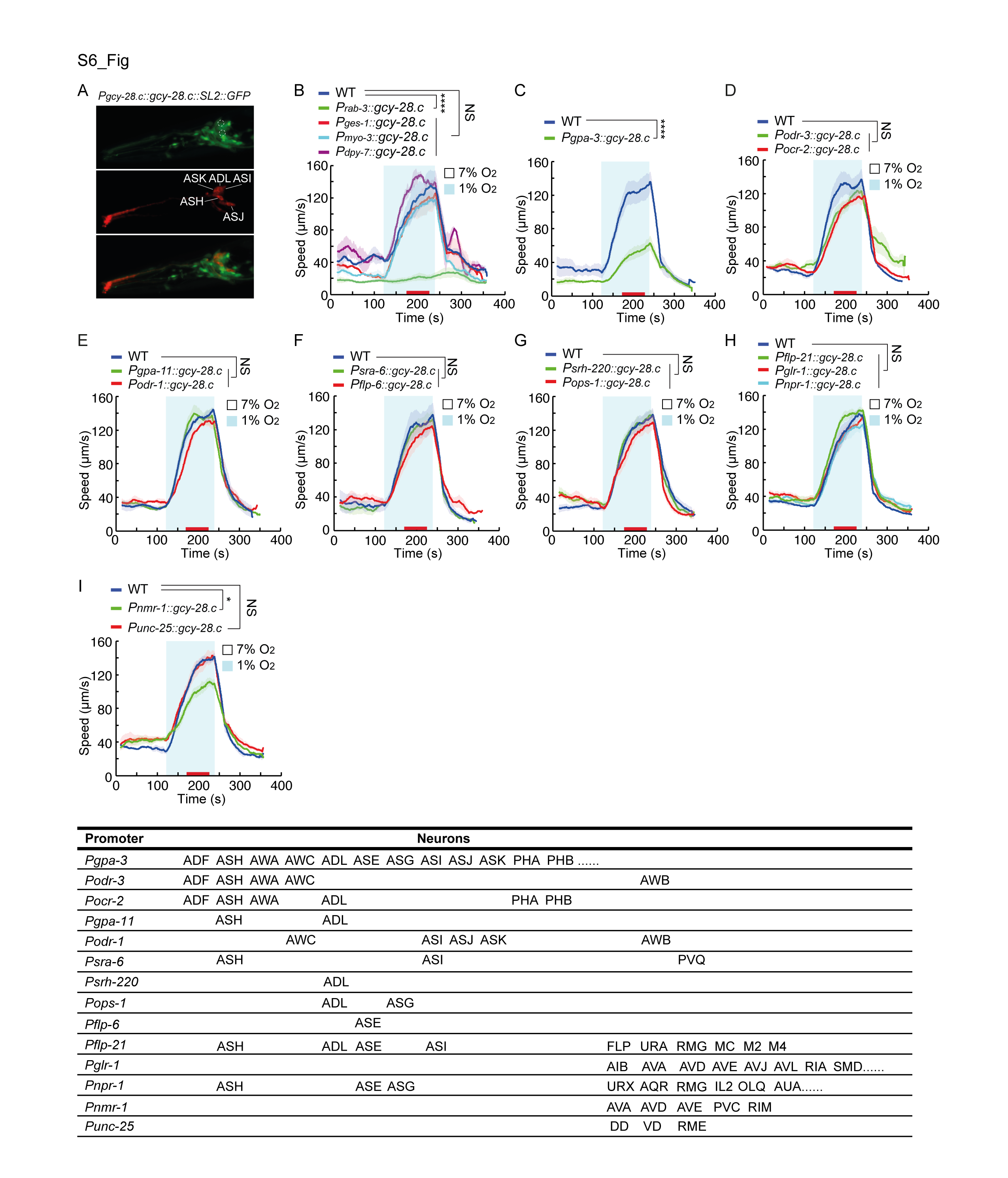

Supplement: S6 Fig — (A) Fluorescence expression of a gcy-28.c::gfp construct driven from its endogenous promoter (top panel). The dashed circles indicate the expected locations of ADL and ASH neurons highlighted by dye-filling with DiI (middle panel), seen overlaid with gcy-28.c::gfp expression (bottom panel). (B) Locomotory responses to hypoxia of transgenic animals expressing gcy-28.c cDNA in neurons (Prab-3), intestine (Pges-1), muscle (Pmyo-3), and hypodermis (Pdpy-7). NS = not significant, **** = p < 0.0001. ANOVA, Tukey multiple comparison. (C–I) Locomotory responses to hypoxia of transgenic animals overexpressing gcy-28.c in different subsets of neurons. The detailed expression pattern of each promoter is listed in the table. NS = not significant, * = p < 0.05, **** = p < 0.0001. Mann–Whitney U test (C), and ANOVA, Tukey multiple comparison (D–I). The source code underlying behavioral data can be found at https://github.com/wormtracker/zentracker. (TIF) [file pbio.3001684.s006.tif]

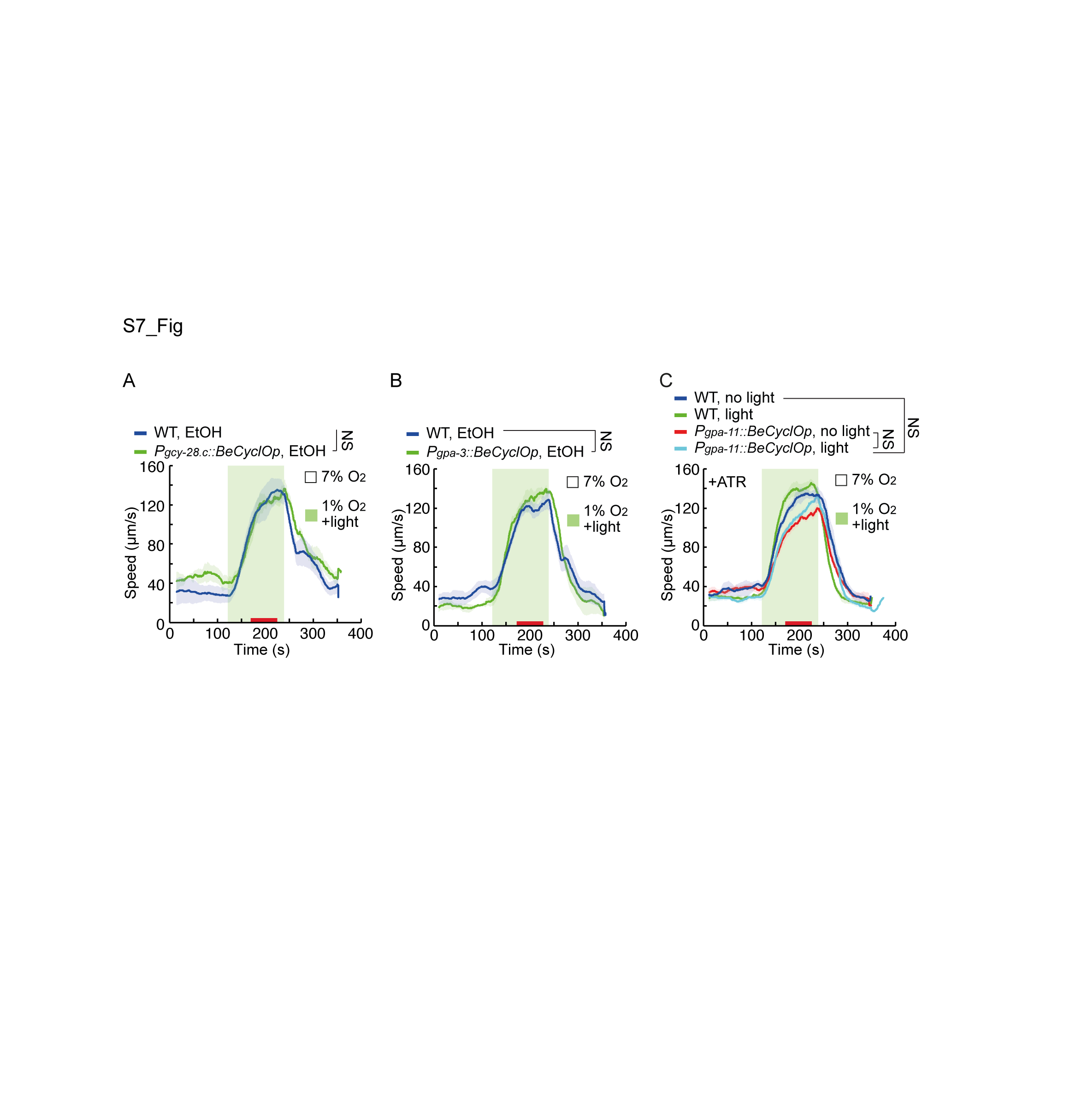

Supplement: S7 Fig — (A and B) Locomotory responses to hypoxia of WT animals expressing BeCyclOp from the gcy-28.c promoter (A), or the gpa-3 promoter (B), without ATR treatment but with blue light exposure. NS = not significant, Mann–Whitney U test. (C) WT animals expressing BeCyclOp from the gpa-11 promoter in the presence of ATR do not show light-dependent inhibition of hypoxia–evoked locomotory responses. NS = not significant. ANOVA, Tukey multiple comparison. The source code underlying behavioral data can be found at https://github.com/wormtracker/zentracker. WT, wild-type. (TIF) [file pbio.3001684.s007.tif]

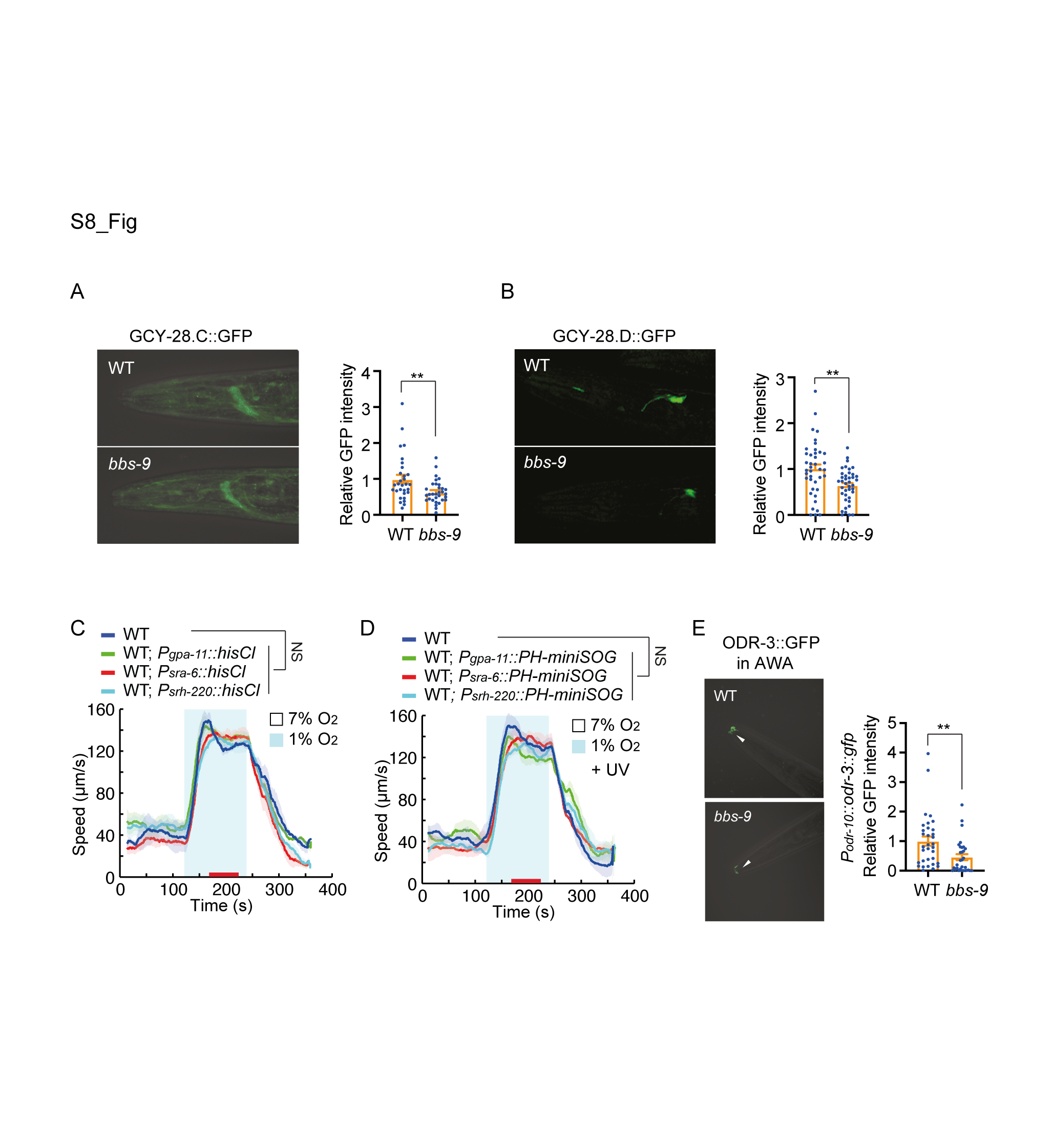

Supplement: S8 Fig — (A) Representative images (left panels) and quantification (right panel) of GFP fluorescence from expression of a single copy GCY-28.C-GFP inserted using MosSCI. ** = p < 0.01, t test. (B) Representative images (left panels) and quantification (right panel) of GFP fluorescence from expression of an extrachromosomal array of GCY-28.D-GFP in AIA neurons. ** = p < 0.01, t test. (C) Locomotory responses to 7% to 1% O2 stimuli of animals treated with 10 mM histamine: WT, and WT expressing HisCl1 from sra-6 (ASH), srh-220 (ADL) and gpa-11 (ASH and ADL) promoters. NS = not significant. ANOVA, Tukey multiple comparison. (D) Locomotory responses to 7% to 1% O2 stimuli of animals treated with 2 mW/mm2 blue light for 5 minutes: WT, and WT expressing PH-miniSOG from the sra-6 (ASH), srh-220 (ADL) and gpa-11 (ASH and ADL) promoters. NS = not significant. ANOVA, Tukey multiple comparison. (E) Representative images (left panels) and quantification (right panel) of ODR-3::GFP in AWA cilia. Arrowheads point to AWA cilia. The GFP intensity in WT was arbitrarily set to 1, and the GFP signal in bbs-9(gk471) mutants was normalized to WT. ** = p < 0.01, t test. The underlying data can be found in S1 Data, and the source code can be found at https://github.com/wormtracker/zentracker. O2, oxygen; WT, wild-type. (TIF) [file pbio.3001684.s008.tif]

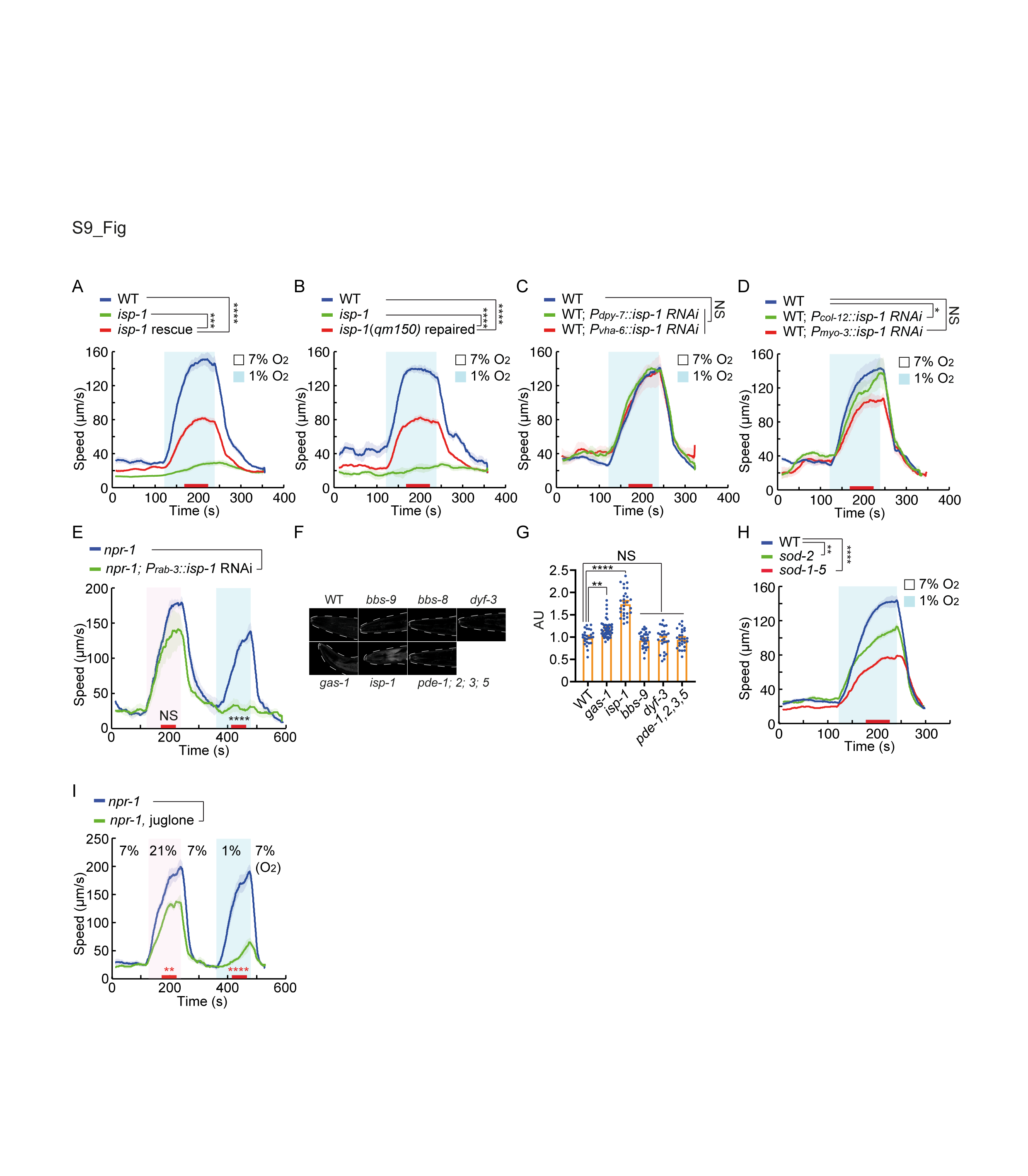

Supplement: S9 Fig — (A) Locomotory responses to 7% to 1% O2 stimuli of animals of indicated genotypes: WT, isp-1(qm150), and isp-1(qm150) expressing 2 isp-1 containing fosmids. **** = p < 0.0001, *** = p < 0.001, NS = not significant. ANOVA, Tukey multiple comparison. (B) Locomotory responses to 7% to 1% O2 stimuli of animals of indicated genotypes: WT, isp-1(qm150), and CRISPR repaired isp-1(qm150), in which serine at position 225 was replaced by proline as in WT. **** = p < 0.0001. ANOVA, Tukey multiple comparison. (C and D) Locomotory responses to 7% to 1% O2 stimuli of animals of indicated genotypes: WT, and WT expressing isp-1 RNAi constructs under dpy-7 (hypodermis) and vha-6 (intestine) promoters (C); WT, and WT expressing isp-1 RNAi constructs under col-12 (epithelium) and myo-3 (muscle) promoters (D). * = p < 0.05, NS = not significant. ANOVA, Tukey multiple comparison. (E) Locomotory responses to indicated changes in O2 concentration of npr-1(ad609) and npr-1(ad609) expressing isp-1 RNAi constructs pan-neuronally. NS = not significant (21% O2), **** = p < 0.0001 (1% O2). Mann–Whitney U test. (F) Representative images of DHE staining of various genotypes. (G) Quantification of DHE staining. ** = p < 0.01, **** = p < 0.0001, NS = not significant. ANOVA, Tukey multiple comparison. (H) Hypoxia-evoked locomotory responses of WT, sod-2(ok1030), and sod-2(ok1030); sod-5 (tm1146) sod-1(tm783); sod-4(gk101); sod-3(tm760) mutants. ** = p < 0.01, **** = p < 0.0001. ANOVA, Tukey multiple comparison. (I) Animals were exposed to 1 mM juglone for 2 minutes and immediately assayed for their locomotory responses to 21% and 1% O2. ** = p < 0.01, **** = p < 0.0001. Mann–Whitney U test. The underlying data can be found in S1 Data, and the source code can be found at https://github.com/wormtracker/zentracker. DHE, dihydroethidium; O2, oxygen; RNAi, RNA interference; WT, wild-type. (TIF) [file pbio.3001684.s009.tif]

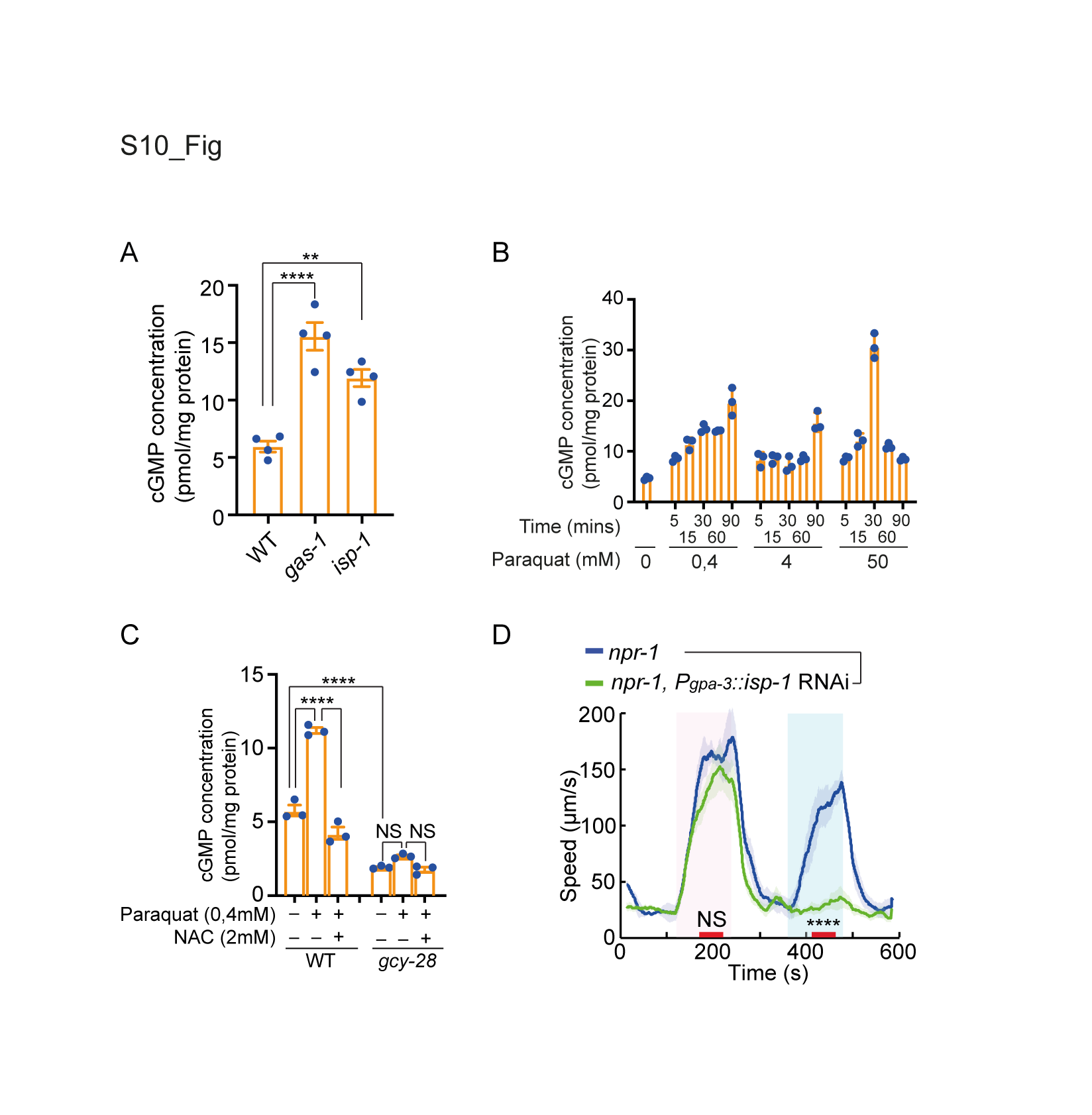

Supplement: S10 Fig — (A) Total cGMP in worm lysates determined by cGMP enzyme immunoassay of indicated genotypes (n = 4): WT, gas-1(fc21) and isp-1(qm150). **** = p < 0.0001, and ** = p < 0.01. ANOVA, Tukey multiple comparison. (B) Staged L4 animals were exposed to 0.4 mM, 4 mM or 50 mM paraquat for different time periods, and their total cGMP levels determined by ELISA (n = 3). (C) Total cGMP levels in worm lysates of indicated genotypes treated with or without 0.4 mM paraquat and 2 mM NAC. cGMP levels were measured using a cGMP enzyme immunoassay. **** = p < 0.0001, NS = not significant. ANOVA, Tukey multiple comparison. (D) Locomotory responses to indicated changes in O2 concentration of npr-1(ad609) and npr-1(ad609) expressing isp-1 RNAi constructs from the gpa-3 promoter. NS = not significant (21% O2), **** = p < 0.0001 (1% O2), Mann–Whitney U test. The underlying data can be found in S1 Data, and the source code can be found at https://github.com/wormtracker/zentracker. NAC, N-acetylcysteine; RNAi, RNA interference; ROS, reactive oxygen species; O2, oxygen; WT, wild-type. (TIF) [file pbio.3001684.s010.tif]
