# Supplementary material for: ROS and cGMP signaling modulate persistent escape from hypoxia in Caenorhabditis elegans
Source: PLoS Biol. 2022 Jun 21;20(6):e3001684. doi: 10.1371/journal.pbio.3001684 (PMC9249223; doi:10.1371/journal.pbio.3001684)
Supplement: S1 Table — The response of each mutant was compared to that of WT. (−) indicates 0% to 10% of WT responses; (+) indicates 10% to 50% of WT responses; (++) indicates 50% to 80% of WT responses; and (+++) indicates 80% to 100% of WT responses. (*) in the table indicates that the strain CHS10032 has a unique response to acute hypoxia. WT, wild-type. (PDF) [file pbio.3001684.s011.pdf]

Groups	*Strain name*	*Genes*	*Acute hypoxia response*
Neuropeptide	CHS10118	*ins-30;ins-20;daf-28;ins-24;ins-33*	+++
	CHS10056	*ins-14;ins-31;ins-39;ins-1*	+++
	CHS10046	*ins-3;ins-21;ins-22;ins-23*	+++
	CHS10025	*ins-26;ins-32;ins-9;ins-13*	++
	CHS10057	*ins-25;ins-28;ins-5;ins-29;ins-27*	+++
	CHS10080	*ins-4;ins-6;ins-7;ins-8*	+++
	CHS10010	*ins-10;ins-19;ins-35;ins-36a,b*	+++
	CHS10073	*ins-34;ins-2;ins-15;ins-12;ins-11*	++
	CHS10062	*ins-17;ins-18;ins-16;ins-37*	+++
	CHS10032*	*flp-1;flp-23;flp-14;flp-25*	++
	CHS10085	*flp-34;flp-10;flp-27;flp-11*	+++
	CHS10034	*flp-3;flp-6;flp-18;flp-13*	+++
	CHS10033	*flp-4;flp-15;flp-2;flp-16*	++
	CHS10007	*flp-17;flp-22;flp-7;flp-9*	+++
	CHS10063	*flp-20;flp-32;flp-19;flp-33;flp-26*	++
	CHS10009	*flp-12;flp-21;flp-24;flp-5;flp-28*	+++
	CHS10011	*nlp-19;nlp-62;ntc-1;nlp-64*	++
	CHS10148	*nlp-66;nlp-11;nlp-54;nlp-79*	+++
	CHS10013	*nlp-16;nlp-55;nlp-61;nlp-8*	++
	CHS10084	*nlp-20;nlp-43;msrp-7;lury-1*	+++
	CHS10103	*nlp-5;nlp-10;nlp-2;nlp-50*	+++
	CHS10040	*nlp-4;nlp-80;nlp-42;nlp-18*	+++
	CHS10149	*nlp-12;nlp-39;capa-1;nlp-6*	+++
	CHS10111	*nlp-56;nlp-57;nlp-63;nlp-53*	+++
	CHS10065	*nlp-1;nlp-38;nlp-3;nlp-13*	++
	CHS10110	*nlp-41a,b;nlp-45;nlp-17;nlp-77*	+++
	CHS10088	*nlp-60;nlp-68;nlp-67*	+++
	CHS10089	*nlp-70;nlp-76;nlp-36*	+++
	CHS10119	*nlp-58;nlp-14;nlp-15;nlp-47*	+++
	CHS10091	*nlp-48;nlp-52;nlp-78;nlp-40a,b*	++
	CHS10092	*nlp-23;nlp-59;nlp-35;pdf-2*	++
	CHS10066	*nlp-21;nlp-69;nlp-73;pdf-1a,b*	++
	CHS10102	*nlp-9;nlp-32;nlp-26;nlp-24*	+++
	CHS10093	*nlp-34;nlp-33;nlp-27;nlp-25*	+++
	CHS10183	*nlp-30;nlp-29;nlp-31;nlp-28*	+++
	CHS10095	*nlp-49;nlp-51;nlp-22;nlp-46;nlp-71*	+++
	RB2269	*flp-1(ok3071)*	++
	RB2126	*flp-1(ok2811)*	++
	VC2591	*flp-2(ok3351)*	+
	VC2497	*flp-3(ok3265)*	+
	VC1957	*flp-14(gk1055);sfxn-1.2(gk3039)*	++
	RB2592	*flp-17(ok3614)*	++
	VC2016	*flp-18(gk3063)*	++
	RB892	*flp-21(ok889)*	+++
	PT501	*flp-8(pk360)*	++
	VB1409	*ins-1 (tm1888)*	++
	VB1468	*ins-7(tm1907)*	++
	VC1218	*ins-18 (ok1672)*	+

Globin	CHS506	*glb-1(yum12)*	+++
	CHS507	*glb-2(yum13)*	+++
	CHS539	*glb-3(yum29)*	+++
	CHS525	*glb-4(yum22)*	+++
		glb-5(tm5440)	+++
		glb-6(tm3795)	+++
	CHS535	*glb-7(yum27)*	+++
	CHS541	*glb-8(yum30)*	+++
	CHS519	*glb-9(yum19)*	+++
		glb-10(tm5198)	+++
		glb-10(tm5744)	+++
	CHS509	*glb-11(yum14)*	+++
	CHS543	*glb-12(yum31)*	+++
		glb-13(tm2825)	++
		glb-13(tm7824)	++
	CHS2204	*glb-14(yum594)*	++
	CHS511	*glb-15(yum15)*	+++
		glb-16(tm5264)	+++
	CHS513	*glb-17(yum16)*	++
		glb-18(tm5798)	+++
		glb-18(tm6017)	+++
		glb-19(tm6923)	++
		glb-19(tm6965)	++
		glb-20(tm2286)	+++
		glb-21(tm8033)	+++
	CHS529	*glb-22(yum24)*	+++
	CHS515	*glb-23(yum17)*	+++
	CHS517	*glb-24(yum18)*	+++
	CHS531	*glb-25(yum25)*	+++
		glb-26(tm4837)	++
	CHS521	*glb-27(yum20)*	+++
		glb-28(tm6910)	++
		glb-28(tm7157)	+++
	CHS527	*glb-29(yum23)*	+++
	CHS537	*glb-30(yum28)*	+++
	CHS523	*glb-31(yum21)*	+++
	CHS533	*glb-32(yum26)*	+++
		glb-33(tm3656)	++
G protein signaling	DG1856	*goa-1(sa734)*	+++
	NL332	*gpa-1(pk15)*	++
	NL334	*gpa-2(pk16)*	+++
	NL335	*gpa-3(pk35)*	++
	NL2105	*gpa-3(pk35);odr-3(n1605)*	-
	NL348	*gpa-2(pk16);gpa-3(pk35)*	+
	NL790	*gpa-4(pk381)*	++
	NL1137	*gpa-5(pk376)*	+++
	NL1146	*gpa-6(pk480)*	++
	NL795	*gpa-7(pk610)*	+++
	NL1142	*gpa-8(pk345)*	++
	NL793	*gpa-9(pk438)*	+++
	NL1147	*gpa-10(pk362)*	++

	NL787	*gpa-11(pk349)*	++
	NL594	*gpa-12(pk322)*	++
	NL2330	*gpa-13(pk1270)*	+++
	NL788	*gpa-14(pk347)*	++
	NL797	*gpa-15(pk477)*	++
	RB1816	*gpa-16(ok2349)*	+++
	RB1800	*gpa-17(ok2334)*	+++
	CX3222	*odr-3(n1605)*	++
	KG524	*gsa-1(ce94)*	+++
	AX504	*npr-1(ad609);egl-30(n686)*	-
	RB1780	*rgs-1(ok2288)*	++
Guanylate cyclase		*gcy-1(tm2669)*	+++
	VC3024	*gcy-2(ok3721)*	++
	VC2796	*gcy-3(gk1154)*	+++
		gcy-4(tm1653)	++
	RB1010	*gcy-5(ok930)*	+++
	OH4844	*gcy-5(tm897)*	+++
		gcy-6(tm1449)	+++
		gcy-7(tm901)	++
	IK800	*gcy-8(oy44)*	+++
		gcy-9(tm7632)	+++
		gcy-11(tm8150)	+++
		gcy-12(yum589); gcy-13(yum590)	+++
	JN1194	*gcy-14(pe1102)*	++
	VC2675	*gcy-15(gk1102)*	+++
	VC2450	*gcy-17(gk1155)*	+++
	IK597	*gcy-18(nj38);gcy-8(oy44);gcy-23(nj37)*	+++
	RB1909	*gcy-19(ok2472)*	+++
	RB1935	*gcy-20(ok2538)*	++
		gcy-21(tm11147)	+++
		gcy-22(tm2364)	+
	CHS2205	*gcy-22(yum596)*	+++
	RB924	*gcy-23(ok797)*	+++
	IK427	*gcy-23(nj37)*	+++
		gcy-25(tm4300)	+++
		gcy-27(tm11825)	++
		gcy-28(yum32)	+++
	VC1951	*gcy-29(ok2475)*	+++
	DR84	*daf-11(m84)*	+++
	CX2065	*odr-1(n1963)*	+++
	CHS42	*npr-1(ad609);gcy-31syb852);gcy-32;gcy-34;gcy-35;gcy-36;gcy-379(ok384)*	+++
	RB1048	*gcy-32(ok995)*	+++
	AX2362	*gcy-33(ok232);npr-1(ad609)*	+++
	AX1295	*gcy-35(ok769)*	+++
	AX1296	*gcy-36(db42)*	+++
	CHS5086	*shk-1; shl-1*	+++
	CHS5067	*exp-2; kvs-1; kvs-2; kvs-3; kvs-4; kvs-5*	++
	CHS5055	*shw-1; egl-36; shw-3*	+++
	CHS5047	*kqt-1; kqt-2; kqt-3*	+++

Potassium channel	CHS5073	*egl-2; unc-103*	+++
	CHS5100	*slo-1; slo-2*	+++
	CHS5057	*kcnl-1; kcnl-2; kcnl-3; kcnl-4*	+++
	CHS5115	*egl-23; twk-9*	+++
	CHS5105	*sup-9; twk-20*	+++
	CHS5069	*unc-58; unc-110*	++
	CHS5100	*twk-1; twk-2*	++
	CHS5121	*twk-3; twk-10*	+++
	CHS5061	*twk-4; twk-5*	+++
	CHS5085	*twk-6*	+++
	CHS5049	*twk-7; twk-8; twk-40*	++
	CHS5053	*twk-11; twk-12; twk-13*	+++
	CHS5014	*twk-14; twk-16; twk-17*	+++
	CHS5031	*twk-21; twk-22; twk-23*	+++
	CHS5006	*twk-24; twk-26; twk-45*	+++
	CHS5043	*twk-25; twk-33; twk-34; twk-36*	+++
	CHS5038	*twk-28; twk-29; twk-30*	+++
	CHS5025	*twk-31; twk-32; twk-35*	+++
	CHS5081	*twk-37; twk-39; twk-48*	++
	CHS5045	*twk-42; twk-43; twk-44*	+++
	CHS5019	*twk-46;twk-47;twk-49*	+++
		irk-1;irk-2;irk-3	++
		kvs-1(tm2034)	++
	NM1968	*slo-1(js379)*	++
	CX3933	*slo-1(ky389);kyls140*	+
Mitochondrial related genes	CW152	*gas-1(fc21)*	-
	MQ887	*isp-1(qm150)*	-
	TK22	*mev-1(kn1)*	++
	VC1083	*fis-2(gk414)*	+++
	CU5991	*fzo-1(tm1133)*	+
	QC134	*nduf-7(et19)*	+
	PH13	*rad-8(mn163)*	++
	VC2360	*ucr-2.3(ok3073)*	+++
		miro-2(tm2933)	++
		kat-1(tm1037)	++
	KP2048	*ric-7(nu447)*	+
	MT6924	*ric-7(n2657)*	+
	CU6372	*drp-1(tm1108)*	+
	VC2142	*gpd-3(ok2870)*	++
	MQ1333	*nuo-6(qm200)*	++
	RB2434	*asg-2(ok3344)*	+++
	MQ130	*clk-1(qm30)*	++
	CZ19982	*mcu-1(ju1154)*	+++
	RB2547	*pink-1(ok3538)*	+++
		trak-1(tm1572)	+++
	SP1603	*dyf-3(m185)*	-
	VC837	*bbs-1(ok1111)*	-
	VC1168	*bbs-2(gk544)*	+
	CHS928	*bbs-4(yum64)*	-
	RB1268	*bbs-7(ok1351)*	+
	MX52	*bbs-8(nx77)*	-

Cilia mutants	VC1062	*bbs-9(gk471)*	-
	CHS2051	*che-1(p680);npr-1(ad609)*	+++
	CB1033	*che-2(e1033)*	+
	AX1052	*che-3(e1124) npr-1(ad609)*	+
	CB1126	*che-6(e1126)*	+++
	CB3329	*che-10(e1809)*	++
	CB3330	*che-11(e1810)*	++
	CB3323	*che-13(e1805)*	++
	CB3678	*che-14(e1960)*	++
	SP1205	*dyf-1(mn335)*	+
	SP1234	*dyf-2(m160)*	++
	SP1237	*dyf-4(m158)*	+++
	SP1712	*dyf-6(m175)*	+
	RB1146	*dyf-5(ok1170)*	+
	RB1148	*dyf-5(ok1177)*	+
	SP1196	*dyf-7(m539)*	-
	MT3559	*dyf-9(n1513)*	+
	SP1709	*dyf-10(e1383)*	+
	SP1713	*dyf-11(mn392)*	++
	SP1678	*dyf-13(mn396)*	+
	CB1377	*daf-6(e1377)*	++
	VC1641	*daf-10(gk795)*	++
	CHS2039	*daf-19(yum101); daf-12(yum102)*	+
	MX124	*ifta-1(nx61)*	+
	RB743	*nphp-1(ok500)*	++
	PT709	*nphp-4(tm925); him-5(e1490)*	+
	DG2179	*tub-1 (nr2044)*	+
	JT11069	*xbx-1(ok279)*	+
	PT442	*klp-6(sy511);him-5(e1490)*	++
	PT1194	*klp-6(my8); him-5(e1490)*	++
	RP811	*osm-6(p811)*	++
	VC265	*osm-5(ok4451)*	+++
		osm-3(e1806)	+++
	PR808	*osm-1(P808)*	+++
	RB2574	*mks-5 (ok3582)*	+++
	VC1466	*mks-6(gk674);xpa-1*	+++
	RB849	*kap-1(ok676)*	+++
	VC2449	*klp-20(ok2914)*	+++
	AX5364	*arl-3(tm1703);npr-1(ad609)*	+++
	RB2509	*arl-6(ok3472)*	+++
	VC2343	*xbx-10(gk1232)*	+
	VC3240	*dct-14(gk3188)*	++
	OEB800	*tmem107(oq100)*	++
	QC134	*nduf-7(et19)*	+
	CHS398	*daf-25(m98);npr-1(ad609)*	+++
Ion channel	PR691	*tax-2(p691)*	+++
	AX3177	*tax-4(p678);npr-1(ad609)*	+++
	KJ5560	*cng-1(jh111); cng-3(jh113)*	+++
	CB1126	*cng-4(e1126)*	+++
	CX4533	*ocr-1(ok132)*	+++
	VC1233	*ocr-2(ok1711)*	+++

	LX950	*ocr-4(vs137)*	+++
		osm-9(n1601)	+++
	VC160	*trp-1(ok323)*	++
	VC602	*trp-2(gk298)*	++
	VC1141	*trp-4(ok1605)*	+++
	TQ233	*trpa-1(ok999)*	++
	VC2435	*trpa-2(ok3189)*	+++
	EJ26	*gon-2(362)*	+++
	DA1116	*eat-2(ad1116)*	++
	VC244	*gtl-1(ok375)*	++
	CZ9957	*gtl-2 (n2618)*	+++
Biogenic amines	GRB21	*tph-1(mg280)*	+
		cat-2(tm2261)	++
	RB993	*tdc-1(ok914)*	+++
	LC33	*bas-1(tm351)*	++
	RB1161	*tbh-1(ok1196)*	+++
	MT9668	*mod-1(ok103)*	++
others	TJ1052	*age-1(hx546)*	+
	GR1307	*daf-16(mgdf50)*	++
	CB1372	*daf-7(e1372)*	+++
	SET68	*nhr-49 (nr2149)*	++
	BX24	*fat-1(wa9)*	++
	RB969	*fat-2 (ok873)*	+++
	VC788	*fat-3 (ok1126)*	+
	BX30	*fat-3(wa22)*	+
	RB1031	*fat-4 (ok958)*	++
	BX17	*fat-4 (wa17)*	++
	VC127	*pkc-2(ok328)*	+++
	VC1151	*prdx-3(gk529)*	+++
	GA416	*sod-4(gk101)*	+++
	FX1146	*sod-5(tm1146)*	+++
	VZ1	*trx-1(ok1449)*	+++
	VZ13	*trx-2(tm2720)*	+++
	VZ68	*trx-3(tm2820)*	+++
	VC1844	*pde-1(gk906)*	++
	KG744	*pde-4(ce268)*	+++
	RB1231	*pde-4(ok1290)*	+
	RB 2279	*pde-5(tm3617)*	+++
	RB 2470	*pde-6(ok3410)*	+++
	TQ1828	*pde-1(nj57);pde-2(tm3098);pde-3(nj59);pde-5(nj49)*	-
	DA521	*egl-4(ad450)*	+
	MT1073	*egl-4 (n478)*	+++
	KG518	*acy-1(ce2)*	+++
	KG522	*acy-1(md1756)*	+++
	KP1182	*acy-1(nu329)*	+++
	VC2393	*acy-2(ok3003)*	+++
	VC20501	*acy-3*	++
	VC1331	*acy-4(ok1806)*	+
		C36E8.4(tm4502)	++
	PS3551	*hsf-1(sy441)*	-

RE666	*ire-1(v33)*	+
RB1920	*mig-10(ok2499)*	++
BW315	*mig-10 (ct4)*	++
MT1083	*egl-8(n488)*	++
MT1443	*egl-10(n692)*	+
	catp-5(ok3303)	++
JT6130	*daf-21(p673)*	+++
MH2230	*dph-3(ku432)*	++
JT722	*hid-1(sa722)*	++
VC1268	*cank-26(gk567)*	++
RB2070	*pfkp-1.1 (ok2733)*	++
RB2510	*catp-6 (ok3473)*	++
VC816	*ric-4(gk322)*	++
RM956	*ric-4 (md1088)*	++
SJ17	*xbp-1(zc12)*	+++
CHS2049	*aldo-1(yum48)*	++
ZG31	*hif-1 (ia4)*	+++
CB5602	*vhl-1(ok161)*	++
JT307	*egl-9(sa307)*	+++
NM1278	*rbf-1(js232)*	+++
JT24	*aex-6(sa24)*	+++
FX17843	*ador-1(tm3971)*	++
IK130	*pkc-1(ni3)*	+++
VC743	*cyp-35A2(gk326)*	+
VC710	*cyp-35A2(gk317)*	++
RB1498	*hyl-2 (ok1766)*	++

- 0 to 10% of wild type response to acute hypoxia
+ 10 to 50% of wild type response to acute hypoxia
++ 50 to 80% of wild type response to acute hypoxia
+++ 80 to 100% of wild type response to acute hypoxia
* CHS10032 has a high locomotory speed at 7% O_2 and no clear decrease of speed when O_2 is switched back from 1% to 7%.
