# Supplementary material for: ROS and cGMP signaling modulate persistent escape from hypoxia in Caenorhabditis elegans
Source: PLoS Biol. 2022 Jun 21;20(6):e3001684. doi: 10.1371/journal.pbio.3001684 (PMC9249223; doi:10.1371/journal.pbio.3001684)
Supplement: S2 Table — (DOCX) [file pbio.3001684.s012.docx]

**S2 Table: Strain list**

| **strain name** | **genotype** |
| --- | --- |
|  | N2, *C. elegans* wild type |
| DA609 | *npr-1(ad609)* |
| AX1197 | *npr-1(ad609);gcy-35(ok769)I* |
| AX3177 | *npr-1(ad609);tax-4(p678)III* |
| ZG31 | *hif-1(ia4)* |
| JT307 | *egl-9(sa307)* |
| SP1603 | *dyf-3(m185)* |
| CHS2054 | *dyf-3(m185);yumEx[pdyf-3::dyf-3::sl2mcherry]* |
| VC1062 | *bbs-9(gk471)* |
| CHS2057 | *bbs-9(gk471); yumEx[posm-6::bbs-9::sl2gfp]* |
| PR811 | *osm-6(p811)* |
| CHS976 | *osm-6(p811);bbs-9(gk471)* |
| MT1073 | *egl-4(n478)* |
| CHS700 | *dyf-3(yum41);egl-4(n478)* |
| CHS333 | *bbs-9(gk471);egl-4(n478)* |
| CHS549 | *egl-4(yum34[T276A])* |
| CHS499 | *bbs-9(gk471);egl-4(yum34[T276A])* |
| TQ1828 | *pde-1(nj57);pde-5(nj49);pde-3(nj59);pde-2(tm3098)* |
| CW152 | *gas-1(fc21)* |
| MQ887 | *isp-1(qm150)* |
| NL2105 | *gpa-3(pk35);odr-3(n1605)* |
| CHS502 | *gcy-28(yum32)* |
| CHS498 | *bbs-9(gk471);gcy-28(yum32)* |
| CHS645 | *N2; yumEx[Pgcy-28c::gcy-28c::sl2gfp]* |
| CHS616 | *N2; yumEx[Pgcy-28c::gcy-28d::sl2gfp]* |
| CHS821 | *N2; yumEx[Psra-9::FlincG3+Psra-9::mcherry]* |
| CHS940 | *bbs-9(gk471); yumEx[Psra-9::FlincG3+Psra-9::mcherry]* |
| NL335 | *gpa-3(pk35)* |
| CX3222 | *odr-3(n1605)* |
| CHS351 | *gpa-3(pk35);odr-3(n1605);egl-4(n478)* |
| CHS701 | *gpa-3(pk35);odr-3(n1605);gcy-28(yum32)* |
| CHS815 | *bbs-9(gk471); yumEx[Pgpa-3::gpa-3::sl2gfp+Pgpa-3::odr-3::sl2gfp]* |
| CHS214 | *N2; yumEx[Pgpa-11::odr-3::GFP]* |
| CHS265 | *bbs-9(gk471); yumEx[Pgpa-11::odr-3::GFP]* |
| CHS250 | *N2; yumEx[Pgpa-11::gpa-3::GFP]* |
| CHS225 | *bbs-9(gk471); yumEx[Pgpa-11::gpa-3::GFP]* |
| CX3260 | *N2; kyIs37[Podr-10::odr-10::GFP; lin-15(+)]* |
| CHS779 | *bbs-9(gk471); kyIs37[Podr-10::odr-10::GFP; lin-15(+)]* |
| JT24 | *aex-6(sa24)* |
| CHS301 | *aex-6(sa24);bbs-9(gk471)* |
| NM1278 | *rbf-1(js232)* |
| CHS235 | *bbs-9(gk471); rbf-1(js232)* |
| KQ1254 | *N2; ftls25[psrh-220::daf-28::mcherry; pmyo-2::gfp; punc-122::gfp]* |
| CHS823 | *bbs-9(gk471);ftls25[psrh-220::daf-28::mcherry; pmyo-2::gfp; punc-122::gfp]* |
| CHS825 | *gcy-28(yum32);ftls25[psrh-220::daf-28::mcherry; pmyo-2::gfp; punc-122::gfp]* |
| CHS826 | *bbs-9(gk471);gcy-28(yum32);ftls25[psrh-220::daf-28::mcherry; pmyo-2::gfp; punc-122::gfp]* |
| CHS977 | *rbf-1(js232);yumEx[Pgcy-28c::gcy-28c::sl2gfp]* |
| CC44 | *dyf-3(m185);ccEx9[psra-6::dyf-3::sl2mcherry]* |
| CC42 | *dyf-3(m185);ccEx8[psrh-220::dyf-3::sl2mcherry]* |
| CHS2055 | *dyf-3(m185);yumEx[pgpa-11::dyf-3::sl2mcherry]* |
| CHS117 | *bbs-9(gk471);yumEx[psra-6::bbs-9::sl2gfp]* |
| CHS115 | *bbs-9(gk471);yumEx[psrh-220::bbs-9::sl2gfp]* |
| CHS109 | *bbs-9(gk471);yumEx[pgpa-11::bbs-9::sl2gfp]* |
| CHS717 | *N2; yumEx[prab-3::gcy-28.c::sl2gfp]* |
| CHS2065 | *N2; yumEx[prab-3::gcy-28.d::sl2gfp]* |
| CHS641 | *N2; yumEx[pgcy-28.d::gcy-28.c::sl2gfp]* |
| CHS613 | *N2; yumEx[pgcy-28.d::gcy-28.d::gfp]* |
| CHS714 | *N2; yumEx[pgcy-28.c::BeCyclOp::sl2gfp]* |
| CHS985 | *N2; yumEx[pgpa-3::BeCyclOp::sl2gfp]* |
| CHS859 | *N2; yumEx[pgcy-28.d::BeCyclOp::sl2gfp]* |
| AX6078 | *N2; dbEx[pRMG::YC2.60::unc-54 3'UTR]* |
| AX3931 | *N2; dbEx651[psra-9::YC3.60::unc-54 3'UTR]* |
| CHS876 | *N2; yumEx[pgcy-8::YC3.60::unc-54 3'UTR]* |
| CHS874 | *N2; yumEx[pgcy-28d::YC3.60::unc-54 3'UTR]* |
| CHS10 | *gas-1(fc21);yumEx[gas-1::gas-1::sl2gfp]* |
| MQ989 | *isp-1(qm150);ctb-1(qm189)* |
| CHS2064 | *gas-1(fc21); gcy-28(yum32)* |
| VC837 | *bbs-1(ok1111)* |
| VC1569 | *bbs-2(ok2053)* |
| CHS928 | *bbs-4(yum64)* |
| RB1268 | *osm-12/bbs-7(ok1351)* |
| MX52 | *bbs-8(nx77)* |
| CC59 | *npr-1(ad609);dyf-3(m185)* |
| CHS134 | *npr-1(ad609);bbs-9(gk471)* |
| DA521 | *egl-4(ad450)* |
| CHS700 | *dyf-3(yum41);egl-4(n478)* |
| CHS699 | *dyf-3(yum40);gcy-28(yum32)* |
| FX03765 | *pde-1(tm3765)* |
| FX03098 | *pde-2(tm3098)* |
| VC20675 | *pde-3(gk369949)* |
| RB2279 | *pde-5(ok3102)* |
| CHS403 | *pde-1(tm3765);pde-2(tm3098)* |
| CHS404 | *pde-1(tm3765);pde-3(gk369949)* |
| CHS400 | *pde-1(tm3765);pde-5(ok3102)* |
| CHS401 | *pde-2(tm3098);pde-3(gk369949)* |
| CHS407 | *pde-2(tm3098);pde-5(ok3102)* |
| CHS405 | *pde-3(gk369949);pde-5(ok3102)* |
| CHS785 | *pde-4(ok1290);pde-6(ok3410)* |
| PR678 | *tax-4(p678)* |
| CHS338 | *bbs-9(gk471);tax-4(p678)* |
| KJ5560 | *cng-1(jh111);cng-3(jh113)* |
| CHS603 | *cng-1(jh111);cng-3(jh113);bbs-9(yum38)* |
| CB1126 | *cng-4(e1126)* |
| CHS2063 | *bbs-9(gk471);cng-4(e1126)* |
| CHS56 | *npr-1(ad609);gcy-31(syb852);gcy-32(ok995);gcy-33(syb842);gcy-34(ok1012);gcy-35;gcy-36(db42);gcy-37* |
| CHS395 | *npr-1(ad609);gcy-31(syb852);gcy-32(ok995);gcy-33(syb842);gcy-34(ok1012);gcy-35;gcy-36(db42);gcy-37;bbs-9(yum10)* |
| CHS495 | *bbs-9(gk471);gcy-1(yum78)* |
| CHS497 | *bbs-9(gk471);gcy-13(yum85)* |
| CHS496 | *bbs-9(gk471);gcy-21(yum79)* |
| CHS494 | *bbs-9(gk471);gcy-27(yum77)* |
| CHS493 | *bbs-9(yum76);gcy-23(nj37);gcy-8(oy44);gcy-18(nj38)* |
| CHS657 | *N2; yumEx[pgcy-28c::gcy-35::sl2gfp]* |
| CHS753 | *N2; yumEx[gcy-15 fosmid]* |
| CHS208 | *N2; yumEX[podr-10::odr-3::gfp]* |
| CHS724 | *bbs-9(gk471);yumEx[podr-10::odr-3::gfp]* |
| CHS2056 | *dyf-3(m185); yumEx[posm-6::dyf-3::sl2mcherry]* |
| CHS107 | *bbs-9(gk471); yumEx[posm-6::bbs-6::sl2gfp]* |
| NL2105 | *gpa-3(pk35);odr-3(n1605)* |
| CHS142 | *gpa-3(pk35);odr-3(n1605);yumEx[pgpa-11::gpa-3::sl2gfp]* |
| CHS137 | *gpa-3(pk35);odr-3(n1605);yumEx[psra-6::gpa-3::sl2gfp]* |
| CHS2058 | *gpa-3(pk35);odr-3(n1605); yumEx[psrh-220::gpa-3::sl2gfp]* |
| CHS619 | *N2; yumEx[pgcy-28c::gcy-28.c::SL2::GFP]* |
| CHS717 | *N2; yumEx[prab-3::gcy-28.c::sl2gfp]* |
| CHS2059 | *N2; yumEx[pges-1::gcy-28.c::sl2gfp]* |
| CHS2060 | *N2; yumEx[pmyo-3::gcy-28.c::sl2gfp]* |
| CHS2061 | *N2; yumEx[pdpy-7::gcy-28.c::sl2gfp]* |
| CHS643 | *N2; yumEx[pgpa-3::gcy-28.c::sl2gfp]* |
| CHS672 | *N2; yumEx[podr-3::gcy-28.c::sl2gfp]* |
| CHS642 | *N2; yumEx[pocr-2::gcy-28.c::sl2gfp]* |
| CHS644 | *N2; yumEx[pgpa-11::gcy-28.c::sl2gfp]* |
| CHS669 | *N2; yumEx[podr-1::gcy-28.c::sl2gfp]* |
| CHS646 | *N2; yumEx[psra-6::gcy-28.c::sl2gfp]* |
| CHS666 | *N2; yumEx[pflp-6::gcy-28.c::sl2gfp]* |
| CHS647 | *N2; yumEx[psrh-220::gcy-28.c::sl2gfp]* |
| CHS663 | *N2; yumEx[pops-1::gcy-28.c::sl2gfp]* |
| CHS1003 | *N2; yumEx[pflp-21::gcy-28.c::sl2mcherry]* |
| CHS997 | *N2; yumEx[pglr-1::gcy-28.c::sl2mcherry]* |
| CHS1012 | *N2; yumEx[pnpr-1::gcy-28.c::sl2mcherry]* |
| CHS1018 | *N2; yumEx[pnmr-1::gcy-28.c::sl2mcherry]* |
| CHS2062 | *N2; yumEx[punc-25::gcy-28.c::sl2mcherry]* |
| CHS714 | *N2; yumEx[pgcy-28c::BeCyclOp::sl2gfp]* |
| CHS985 | *N2; yumEx[pgpa-3::BeCyclOp::sl2gfp]* |
| CHS386 | *N2; yumEx[pgpa-11::BeCyclOp::sl2gfp]* |
| TQ1828 | *pde-1(nj57);pde-5(nj49)I;pde-3(nj59);pde-2(tm3098)* |
| CHS690 | *egl-4(n478);pde-1(nj57);pde-5(nj49);pde-3(nj59);pde-2(tm3098); gcy-28(yum39)* |
| RB1072 | *sod-2(ok1030)* |
| MQ1766 | *sod-2(ok1030);sod-5(tm1146);sod-1(tm783);sod-4(gk101);sod-3(tm760)* |
| KQ1787 | *N2; Ex[psrh-220::flp-21::mCherry; punc-122::gfp]* |
| CHS2159 | *bbs-9(gk471);yumEX[psrh-220::flp-21::mCherry; punc-122::gfp]* |
| CHS2160 | *gcy-28(yum32); yumEX[psrh-220::flp-21::mCherry; punc-122::gfp]* |
| CHS2161 | *bbs-9(gk471);gcy-28(yum32); yumEX[psrh-220::flp-21::mCherry; punc-122::gfp]* |
| CHS2227 | *N2; yumEX[pgpa-11::PH-miniSOG(Q103L)::sl2gfp]* |
| CHS2228 | *N2; yumEX[psrh-220::PH-miniSOG(Q103L)::sl2gfp]* |
| CHS2229 | *N2; yumEX[psra-6::PH-miniSOG(Q103L)::sl2gfp]* |
| CHS2170 | *N2; yumEx[Pcol-12::isp-1 dsRNA]* |
| CHS2164 | *N2; yumEx[Pmyo-3::isp-1 dsRNA]* |
| CHS2174 | *N2; yumEx[Pgpa-3::isp-1 dsRNA]* |
| CHS2176 | *N2; yumEx[Pgpa-11::isp-1 dsRNA]* |
| CHS2171 | *N2; yumEx[Pgcy-28c::isp-1 dsRNA]* |
| CHS2173 | *N2; yumEx[Pgcy-28d::isp-1 dsRNA]* |
| CHS2169 | *N2; yumEx[Pdpy-7::isp-1 dsRNA]* |
| CHS2165 | *N2; yumEx[Pvha-6::isp-1 dsRNA]* |
| CHS2162 | *N2; yumEx[Prab-3::isp-1 dsRNA]* |
| CHS2230 | *N2; yum[pgpa-11::bbs-9 dsRNA ]* |
| CHS2139 | *N2; yumEx[Pflp-18::FlincG3+Pflp-18::mcherry]* |
| CHS2140 | *bbs-9(gk471);yumEx[Pflp-18::FlincG3+Pflp-18::mcherry]* |
| CHS2141 | *N2; yumEx[Psra-6::FlincG3+Psra-6::mcherry]* |
| CHS2142 | *bbs-9(gk471);yumEx[Psra-6::FlincG3+Psra-6::mcherry]* |
| CHS613 | *N2; yumEx[Pgcy-28d::gcy-28.d::GFP]* |
| CHS680 | *bbs-9(gk471);yumEx[Pgcy-28d::gcy-28.d::GFP]* |
| PHX2236 | *gcy-28(syb2236)::GFP* |
| CHS2197 | *bbs-9(gk471);gcy-28(syb2236)::GFP* |
| CHS2205 | *npr-1(ad609); yumEx[ [prab-3::isp-1 dsRNA ::unc54 3'utr ]* |
| CHS2225 | *isp-1(yum593),ox6 repair* |
| CHS2226 | *isp-1(qm150); Ex[WRM063aA07fosmid+WRM064cD07fosmid ]* |
| CHS497 | *bbs-9(gk471); gcy-13(yum85)* |
| CHS495 | *bbs-9(gk471); gcy-1(yum78)* |
| CHS496 | *bbs-9(gk471); gcy-21(yum79)* |
| CHS494 | *bbs-9(gk471); gcy-27(yum77)* |
| CHS493 | *bbs-9(yum76); gcy-23(nj37); gcy-8(oy44); gcy-18(nj38)* |
